# Dual-interface stabilization of low-iridium anodes for durable proton exchange membrane water electrolysis

**DOI:** 10.1038/s41467-026-75113-6

**Published:** 2026-07-04

**Authors:** Eui Tae Kim, Sangwoo Kim, Sung-Eun Park, Pumsuk Park, Eunbyeol Ko, Jemee Joe, Ho Yeon Son, Juyeon Kang, Julie Junesoo Kim, Kibeom Cheon, Kyungin Kim, Soree Kim, Geunsung Lee, Jaehak Jeong, Manki Cho, Noma Kim, Jai Hyun Koh, Kihwan Kim

**Affiliations:** 1 Platform Technology Research Center, LG Chem, Seoul, Republic of Korea; 2 Analytical Sciences Center, LG Chem, Seoul, Republic of Korea; 3 Clean Energy Research Center, Korea Institute of Science and Technology (KIST), Seoul, Republic of Korea

**Keywords:** Devices for energy harvesting, Electrocatalysis, Chemical engineering, Electrocatalysis, Electrocatalysis

## Abstract

The durability and cost of iridium-based anodes remain key challenges for high-current density operation of proton exchange membrane water electrolyzers. Here, we report a dual-interface stabilization strategy based on atomic layer deposition of TiO_2_ onto IrO_2_ catalysts. The resulting anodes sustain operation at 3.0 A cm^−2^ for 2600 h with near-zero voltage degradation at an iridium loading of 0.4 mg cm^−2^, whereas bare IrO_2_ exhibits continuous voltage decay over 1000 h at a rate of 31.5 mV kh^−1^. Combined experimental characterization and theoretical calculations reveal that interfacial Ti-O-Ir coupling suppresses Ir over-oxidation and dissolution, while the TiO_2_-coated surface strengthens ionomer-catalyst interactions and preserves mass-transport pathways during prolonged operation. This strategy is fully compatible with roll-to-roll manufacturing, enabling industrially scalable implementation. This interfacial engineering approach offers a generalizable design principle for extending electrolyzer lifetime and reducing precious-metal loading across diverse electrochemical energy conversion technologies.

## Abstract (web summary)

Iridium-based anodes for proton exchange membrane water electrolyzers degrade through dissolution and pore collapse at industrial current densities. Here, the authors report that a titania surface modification stabilizes the catalyst and the electrode, enabling durable operation with less iridium

## Introduction

The green hydrogen market has grown rapidly in response to global decarbonization initiatives and has accelerated the deployment of water electrolysis technologies alongside increased industrial research, development, and capital investment^1–5^. Among competing platforms, proton exchange membrane water electrolyzers (PEMWEs) are projected to secure a dominant market position owing to their high energy conversion efficiency, rapid response to load fluctuations, and direct production of high-purity hydrogen at elevated pressures^6,7^. Despite these advantages, commercialization of PEMWEs remains constrained by limited durability at industrially relevant current densities and by the high cost of iridium (Ir)-based anode catalysts. Ir-based materials represent the benchmark catalysts for the oxygen evolution reaction (OER) because of their favorable balance between activity and stability in acidic media, yet Ir scarcity and limited global supply have substantially elevated system-level costs. Furthermore, the inherently slow OER kinetics under highly oxidative anode conditions accelerate catalyst degradation and drive progressive performance loss^8,9^. Accordingly, strategies that enhance durability while minimizing Ir loading are essential for scalable deployment^10–12^.

Reducing Ir loading, however, renders the anode architecture more susceptible to heterogeneity arising from fabrication and prolonged operation. Under highly anodic potentials, a limited Ir reservoir exacerbates degradation pathways, including oxidative dissolution and dynamic surface reconstruction that progressively increase structural non-uniformity over time^13–19^. This structural evolution destabilizes the catalyst-ionomer interface and drives particle agglomeration and pore constriction within the catalyst layer. The loss of electrochemically accessible surface area, combined with the accumulation of oxygen bubbles that induce mass-transport limitations, accelerates performance degradation^20–22^. Consequently, the concurrent stabilization of both the catalyst surface and the catalyst-ionomer interface is required to suppress Ir dissolution-reconstruction cycles while preserving a homogeneous pore architecture^23–25^.

**Fig. 1:**
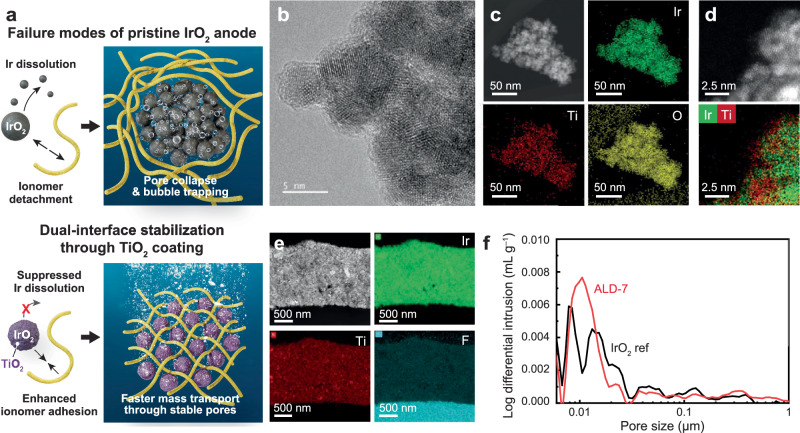
TiO_2_-coated IrO_2_ catalyst and anode architecture enabling durable PEMWEs. **a** Schematic comparisons of degradation pathways in pristine IrO_2_ anodes and the dual-interface stabilization mechanism enabled by TiO_2_ ALD. TiO_2_ coating suppresses Ir dissolution and enhances ionomer adhesion, thereby stabilizing pore structure and facilitating efficient mass transport under high-current operation. Gray, yellow, and purple features correspond to the IrO_2_, ionomer, and TiO_2_-coated IrO_2_, respectively. **b** Representative high-resolution TEM image of the TiO_2_-coated IrO_2_ catalyst prepared with seven ALD cycles (ALD-7). **c**, **d** STEM-EDS elemental maps of an ALD-7 catalyst particle confirming homogeneous spatial distribution of Ir (green), Ti (red), and O (yellow) throughout the catalyst structure. **e** Cross-sectional STEM-EDS elemental maps of an anode fabricated with the ALD-7 catalyst, showing uniform through-thickness distributions of Ir, Ti, and F within the catalyst layer. **f** Pore size distributions measured by mercury intrusion porosimetry for reference IrO_2_ and ALD-7 anodes, showing a more uniform distribution and increased sub-micrometer pore volume for the ALD-7 anode.

Herein, we address these degradation pathways in low-Ir-loaded anodes by developing a TiO_2_-coated IrO_2_ catalyst and integrating it into membrane electrode assemblies (MEAs). This approach employs a dual-interface stabilization strategy that simultaneously modulates catalyst surface chemistry and ionomer-catalyst interactions (Fig. 1a). The formation of an interfacial Ti-O-Ir species at the IrO_2_ surface suppresses excessive Ir oxidation under highly anodic potentials, thereby mitigating Ir dissolution and preserving the catalytic framework^26–29^. The outer TiO_2_-modified surface further enhances interfacial compatibility with the ionomer, promotes a more uniform pore structure, and resists pore collapse during operation. As a result, the optimized anode with an Ir loading of 0.4 mg cm^−2^ sustains operation at 3.0 A cm^−2^ for 2600 h with a near-zero voltage degradation rate. This durability at low Ir loading compares favorably with recently reported PEMWE systems and satisfies the U.S. Department of Energy (DOE) 2026 durability target (≤ 2.3 mV kh^−1^). The catalyst design is also fully compatible with roll-to-roll electrode fabrication and enables the production of 100 cm^2^ MEAs with homogeneous Ir loading across the entire electrode area. Collectively, this approach establishes a scalable pathway toward the commercial deployment of durable PEMWEs operating at high current densities.

## Results

### Characterization of TiO_2_-coated IrO_2_ catalysts and anodes

The dual-interface stabilization strategy was implemented by depositing TiO_2_ onto commercial IrO_2_ nanoparticles via atomic layer deposition (ALD, 3–30 cycles), followed by thermal annealing at 400 ^∘^C for 1 h. These samples were denoted ALD-*n*, where *n* represents the number of ALD cycles. Transmission electron microscopy (TEM) and scanning transmission electron microscopy-energy-dispersive X-ray spectroscopy (STEM-EDS) analyses indicate that ALD-7 exhibits a uniform Ti distribution at the IrO_2_ surface without the formation of a clearly resolved continuous layer (Fig. 1b–d), whereas samples prepared with higher ALD cycle numbers display distinct core–shell structures (i.e., IrO_2_@TiO_2_, Supplementary Fig. 1). X-ray photoelectron spectroscopy (XPS) analysis further confirms that the dispersed Ti species exhibit Ti 2*p*_3/2_ binding energies of 458.5–459.0 eV, characteristic of Ti^4+^ in TiO_2_-like oxide environments (Supplementary Fig. 2). ALD-7 shows a 23% decrease in specific surface area (46.6 m^2^ g^−1^) relative to bare IrO_2_ and preserves the rutile IrO_2_ phase after annealing (Supplementary Figs. 3–5, Supplementary Table 1). Inductively coupled plasma optical emission spectrometry (ICP-OES) and XPS measurements demonstrate a linear increase in both the Ti content and the surface Ti/Ir atomic ratio with increasing ALD cycles, with ALD-7 containing 3.35 wt% Ti (Supplementary Fig. 6). Coffee-ring analyses and contact angle measurements on model films further show that increasing the ALD cycle number progressively enhances surface hydrophilicity up to a saturation plateau beyond 5–7 cycles (Supplementary Figs. 7 and 8). These observations indicate that moderate ALD cycle numbers predominantly yield a TiO_2_-like surface and enable systematic tuning of surface properties to balance intrinsic IrO_2_ activity with TiO_2_-induced stabilization.

At the electrode level, the TiO_2_ modification correlates with measurable changes in pore structure and wettability of the catalyst layer. Cross-sectional STEM-EDS mapping of the ALD-7 anode confirms a uniform distribution of catalyst and ionomer across the entire electrode matrix (Fig. 1e). Mercury intrusion porosimetry shows a slightly narrower pore size distribution along with a higher porosity (50.8%) and a larger total pore area (0.914 m^2^ g^−1^) than those of the reference IrO_2_ anode (Fig. 1f, Supplementary Table 2 and Supplementary Fig. 9). Atomic force microscopy (AFM) height profiles and adhesion energy maps reveal a uniform ionomer distribution across the catalyst surface, and the adhesion energy increases with ALD cycle number, indicating progressively greater ionomer coverage on the catalyst (Supplementary Fig. 10 and Supplementary Table 3)^30,31^. Drop shape analysis further confirms that the ALD-7 anode exhibits modestly higher hydrophilicity and greater aerophobicity than the reference IrO_2_ anode (Supplementary Fig. 11). Although the difference in electrode-level contact angle is small, such wettability changes are consistent with altered adsorption and organization of ionomer sulfonate groups at the catalyst–ionomer interface induced by the underlying surface modification^32–34^. This ionomer reorganization, rather than the macroscopic wettability change alone, contributes to preserving a uniform pore architecture and a favorable interfacial environment during prolonged operation.

### Electrochemical performance validation at 3.0 A cm^−2^

The electrochemical performance of the TiO_2_-modified catalysts was assessed at two complementary levels through rotating disk electrode (RDE) measurements for intrinsic activity and PEMWE single-cell testing at 80 ^∘^C with an Ir loading of 0.4 mg cm^−2^ for electrode-level performance under industrially relevant conditions. RDE measurements revealed that the electrochemically active surface area (ECSA) decreased progressively with increasing ALD cycles, consistent with a partial loss of accessible surface area upon TiO_2_ coating. Nevertheless, the intrinsic catalytic activity (*j*_ECSA_) of ALD-3 to ALD-7 remained comparable to that of state-of-the-art Ir-based catalysts, whereas ALD-10 exhibited inferior intrinsic activity due to excessive surface coverage at higher cycle numbers (Supplementary Fig. 12 and Supplementary Tables 4 and 5).

**Fig. 2:**
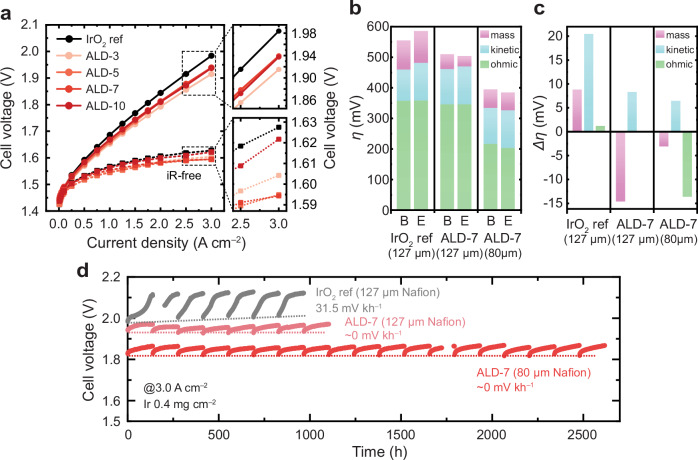
Electrochemical performance and durability of TiO_2_-coated IrO_2_ anodes under high-current PEMWE operation. **a** Polarization curves of PEMWE cells equipped with pristine IrO_2_ and TiO_2_-coated IrO_2_ anodes at 80^∘^C with an Ir loading of 0.4 mg cm^−2^. The insets display magnified voltages in the range of 2.5–3.0 A cm^−2^, highlighting the reduced cell voltage of TiO_2_-coated IrO_2_ anodes. The ohmic resistances of reference IrO_2_, ALD-3, ALD-5, ALD-7, and ALD-10 anodes are 29.7, 25.9, 28.7, 28.8, and 26.3 mΩ, respectively. **b** Quantitative decomposition of the total overpotential at the beginning of test (BoT, B) and end of test (EoT, E) into mass-transport (*η*_mt_), kinetic (*η*_kin_), and ohmic (*η*_ohm_) contributions for reference and ALD-7 anodes. **c** Changes in individual overpotential components (*Δη*) between BoT and EoT, showing suppression of mass-transport and kinetic loss growth for the TiO_2_-modified anodes relative to the reference IrO_2_ anode. **d** Cell-voltage evolution during galvanostatic operation at 3.0 A cm^−2^ for reference IrO_2_ and ALD-7 anodes paired with Nafion membranes of different thicknesses. The ALD-7 anodes exhibit near-zero voltage degradation over 2600 h, whereas the reference IrO_2_ anode shows continuous voltage decay.

Under polarization testing in PEMWE single-cell measurements, all TiO_2_-modified anodes (3, 5, and 7 cycles), except for ALD-10, exhibited an approximately 20–30 mV reduction in iR-free cell voltage at 3.0 A cm^−2^ compared with the reference IrO_2_ anode (Fig. 2a). The observed voltage reduction was not driven by an increase in intrinsic catalytic activity, but rather by enhanced mass transport arising from improved pore structure and wettability. The slightly higher iR-free voltage of ALD-3 reflects incomplete interfacial optimization at insufficient TiO_2_ coverage, whereas the inferior performance of ALD-10 reflects compromised active-site accessibility at excessive coverage. Based on the trend in iR-free voltage (ALD-5  ~ ALD-7  < ALD-3  < ALD-10  < IrO_2_ reference), ALD-7 was selected as the representative anode in this study, as it provides a favorable balance between performance and durability.

PEMWE cells were subsequently evaluated for long-term stability under galvanostatic operation at 3.0 A cm^−2^ that represents a stringent durability condition. When paired with a 127 μm membrane, a thickness commonly used in PEMWE research, the reference IrO_2_ anode exhibits continuous degradation over 1000 h with a decay rate of 31.5 mV kh^−1^. In contrast, all TiO_2_-modified anodes exhibit negligible degradation over 1100–1500 h with the same membrane (Fig. 2d and Supplementary Fig. 13). The reversible voltage increase is substantially suppressed upon TiO_2_ coating (~30 mV) compared with that of the reference IrO_2_ anode (~100 mV). This behavior is consistent with the mitigation of mass-transport limitations associated with oxygen bubble accumulation within the catalyst layer, which obstructs reactant supply and hinders efficient gas removal under high-current-density operation^35^. The threefold reduction in voltage oscillation amplitude provides in situ functional evidence of suppressed bubble-induced transport limitations during 3.0 A cm^−2^ operation, complementing the enhanced aerophobicity observed in ex situ wettability measurements (Supplementary Fig. 11). When paired with an 80 μm membrane, representative of low-resistance industrial operation, the ALD-7 anode operates stably for 2600 h, further underscoring the durability enhancement under harsh operating conditions.

Durability benchmarking against recent PEMWE reports was performed by quantifying the cumulative charge density as the product of current density and time (*j* ⋅ *t*, A h cm^−2^), a metric that captures the total electrochemical severity experienced during prolonged operation (Supplementary Note 1 and Supplementary Table 6). The ALD-7 anode paired with an 80 μm membrane reaches 7800 A h cm^−2^ with near-zero voltage degradation, thereby achieving both the highest cumulative charge and one of the lowest degradation rates among the surveyed systems. Normalization by Ir loading yielded the highest charge density per unit Ir at 3.0 A cm^−2^, indicating highly efficient Ir utilization. These comparisons demonstrate that the present anode architecture effectively decouples high-current operation from accelerated degradation, enabling prolonged PEMWE operation under industrially relevant conditions without reliance on excessive Ir loading.

The origin of this enhanced durability was clarified through mechanistic overpotential analysis. For all TiO_2_-modified anodes (3–7 cycles), a gradual reduction in overall cell voltage was observed during the initial 400–600 h of operation, whereas the reference IrO_2_ anode exhibited a monotonic voltage increase from the onset of testing (Supplementary Fig. 13). This contrast suggests that TiO_2_-induced improvements in wettability and pore integrity facilitate progressive interfacial wetting, thereby mitigating transport-related losses prior to the establishment of steady-state degradation. To quantify this effect, the total overpotential (*η*_total_) at 3.0 A cm^−2^ was deconvoluted into mass-transport (*η*_mt_), kinetic (*η*_kin_), and ohmic (*η*_ohm_) contributions at both the beginning-of-test (BoT) and the end-of-test (EoT) states (Fig. 2b, c, Supplementary Fig. 14 and Supplementary Table 7). At BoT, the ALD-7 anode exhibited a significantly lower *η*_mt_ (48.6 mV) compared to the reference IrO_2_ anode (94.9 mV), which can be attributed to its improved wettability and more open pore structure (Supplementary Table 8). For the reference IrO_2_ anode, 1000 h of operation led to pronounced increases in all overpotential components, with *Δη*_mt _= 8.8 mV, *Δη*_kin _= 20.4 mV, and *Δη*_ohm _= 1.2 mV, indicating degradation of both catalyst-layer structure and intrinsic catalytic activity. In contrast, for the ALD-7 anodes, *η*_mt_ decreases by 14.6 mV for the 127 μm membrane and by 3.1 mV for the 80 μm membrane, respectively. This negative *Δη*_mt_ value corroborates the early-stage voltage reduction and is consistent with alleviated diffusion limitations within the catalyst layer. The increase in *η*_kin_, on the other hand, remains modest relative to that of the reference IrO_2_ anode, suggesting that Ir active sites are preserved. For the 80 μm membrane, *η*_ohm_ decreases significantly by 13.6 mV mainly due to membrane thinning that shortens proton conduction pathways^36^. The stabilized transport environment in the ALD-7 anode also mitigates dehydration at the membrane–electrode interface and further limits additional ohmic losses^37,38^. Overall, the reductions in *η*_mt_ and *η*_ohm_ outweigh the smaller increases in *η*_kin_ and lead to a net decrease in *η*_total_. This synergistic mitigation of both kinetic- and transport-related degradation pathways accounts for enhanced durability.

**Fig. 3:**
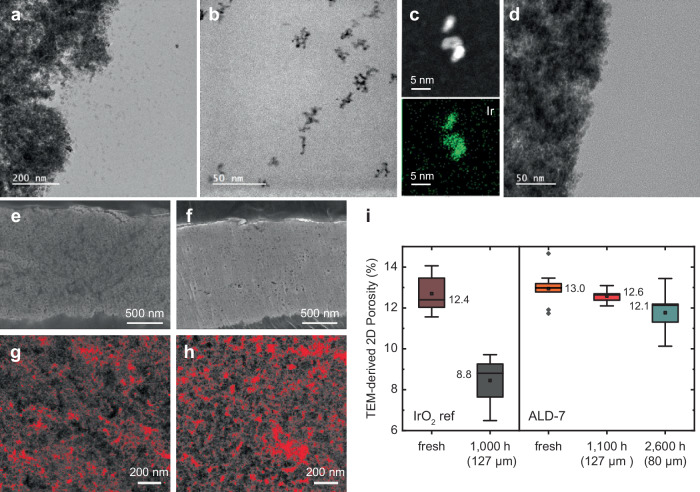
Post-mortem structural analysis of reference and TiO_2_-coated IrO_2_ anodes after long-term PEMWE operation. Samples were assembled with a 127 μm membrane unless otherwise indicated. **a**, **b** Cross-sectional TEM images of the reference IrO_2_ anode after 1000 h of operation at 3.0 A cm^−2^, showing pronounced migration and accumulation of dissolved Ir species within the membrane region. **c** STEM-EDS Ir elemental map confirming the presence of Ir species (green) in the membrane following long-term operation of the reference IrO_2_ anode. **d** Cross-sectional TEM image of the ALD-7 anode after 1100 h of operation, showing no detectable Ir migration into the membrane region. **e**, **f** Cross-sectional FE-SEM images of the reference IrO_2_ anode after 1000 h (**e**) and the ALD-7 anode after 1100 h (**f**). The ALD-7 anode retains an open and interconnected porous morphology, whereas the reference IrO_2_ anode exhibits substantial pore compaction. **g**, **h** Cross-sectional TEM images used for digital porosity analysis of the reference IrO_2_ anode after 1000 h (**g**) and the ALD-7 anode after 1100 h (**h**). Red-highlighted regions indicate pore areas identified by image segmentation. The ALD-7 anode exhibits a larger pore-area fraction than the reference, indicating that the porous structure is preserved after prolonged operation. **i** Statistical quantification of catalyst-layer porosity (%) derived from TEM-based 2D digital image analysis for reference IrO_2_ (127 μm membrane) and ALD-7 anodes (127 and 80 μm membranes) before and after operation, showing pronounced porosity loss for the reference anode and preservation of pore volume for the TiO_2_-modified anode even after 2600 h of operation with the 80 μm membrane.

Post-mortem microscopy provided direct structural evidence linking enhanced durability to the proposed stabilization mechanisms. Cross-sectional TEM images reveal pronounced Ir migration into the membrane region for the reference IrO_2_ anode after 1000 h at 3.0 A cm^−2^, originating from dissolution of Ir species under highly anodic conditions (Fig. 3a–c). In contrast, the ALD-7 anode shows no detectable Ir migration after 1100 h of operation (Fig. 3d). Inductively coupled plasma mass spectrometry (ICP-MS) analysis of the anode outlet water revealed substantially lower Ir dissolution for the ALD-7 anode than for the reference IrO_2_ anode, with the dissolved Ir concentration remaining near the practical detection limit throughout operation (Supplementary Fig. 15). Independent ICP-OES analysis from three-electrode stability tests further confirms that the TiO_2_ coating effectively suppressed Ir dissolution (Supplementary Figs. 16–19). Cross-sectional field-emission scanning electron microscopy (FE-SEM) and TEM images reveal substantial pore collapse in the reference IrO_2_ anode, whereas the ALD-7 anode preserved a well-defined porous structure (Fig. 3e–h and Supplementary Figs. 20, 21). Quantitative porosity analysis derived from TEM images also shows a decrease in porosity of the reference IrO_2_ anode from 12.4% to 8.8% after operation with a 127 μm membrane, while the porosity of the ALD-7 anode changed marginally from 13.0% to 12.6% under the same conditions, and remained largely preserved at 12.1% even after extended operation for 2600 h with an 80 μm membrane (Fig. 3i). This structural evolution directly correlates with the change in mass-transport overpotential, where the reference anode exhibits an increase in *η*_mt_ due to pore collapse, whereas the ALD-7 anode shows a decrease, consistent with preserved transport pathways (Supplementary Table 9 and Supplementary Fig. 22). Together, these observations demonstrate that the reference IrO_2_ anode degraded through simultaneous Ir dissolution and pore collapse, whereas the TiO_2_-modified anode effectively suppressed both degradation pathways through the dual-interface stabilization mechanism.

### Electronic modulation of IrO_2_ by TiO_2_ coatings

**Fig. 4:**
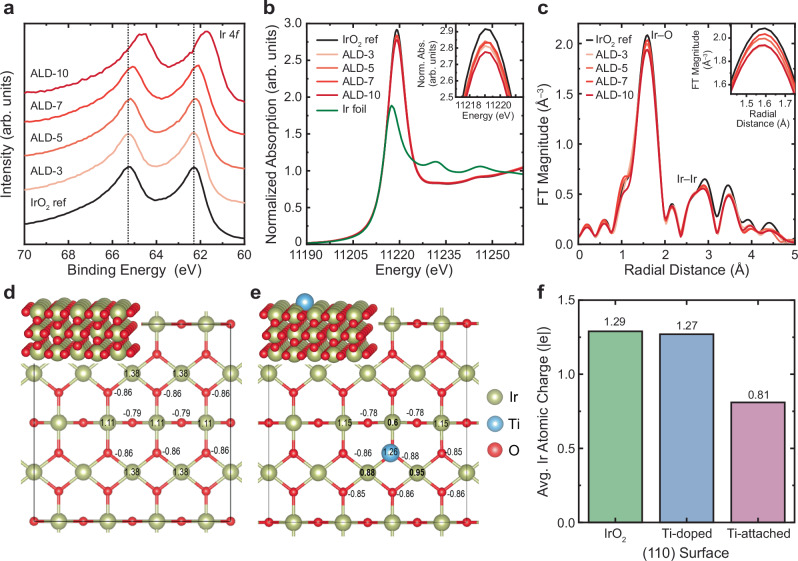
Electronic modulation of IrO_2_ induced by TiO_2_ coating suppresses Ir over-oxidation. **a** XPS Ir 4*f* narrow scans for pristine IrO_2_ and TiO_2_-coated IrO_2_ catalysts as a function of ALD cycle number, showing systematic shifts toward lower binding energy relative to the reference IrO_2_. **b** Ir L_3_-edge XANES spectra showing a progressive decrease in white-line intensity with increasing TiO_2_-ALD cycle number (inset: magnified white-line region), indicating a reduced average Ir oxidation state. **c** Fourier-transformed EXAFS spectra at the Ir edge showing a gradual reduction in Ir-O coordination with increasing ALD cycle number (inset: magnified Ir-O peak region). **d** DFT-calculated atomic model of the pristine IrO_2_(110) surface shown in top and side views, illustrating a relatively uniform distribution of Ir atomic charges. **e** DFT-calculated atomic model of the Ti-attached IrO_2_(110) surface shown in top and side views, revealing pronounced local charge redistribution and reduced Ir atomic charges in the vicinity of the Ti moiety. **f** Comparison of average Ir atomic charges for pristine IrO_2_, Ti-doped, and Ti-attached configurations, showing a significant decrease in average Ir atomic charge for the Ti-attached structure. This charge reduction indicates Ti-induced electronic modulation that stabilizes Ir against over-oxidation under anodic PEMWE operating conditions.

We subsequently investigated the electronic origin of the dual-interface stabilization mechanism through combined spectroscopic and computational analyses. XPS Ir 4*f* spectra show a progressive shift toward lower binding energies with increasing TiO_2_-ALD cycle number, indicating a gradual reduction of the Ir oxidation state (Fig. 4a and Supplementary Fig. 23)^28,39^. Ir L_3_-edge X-ray absorption near edge structure (XANES) reveals a systematic decrease in white-line intensity accompanied by a negative edge shift, while extended X-ray absorption fine structure (EXAFS) shows weakened scattering amplitude with increasing ALD cycles (Fig. 4b, c and Supplementary Fig. 24). These spectroscopic trends demonstrate that interfacial Ti-O-Ir coupling, rather than passive coverage by a bulk TiO_2_ phase, lowers the Ir valence state^40–43^.

To model the Ti-O-Ir interfacial interaction, we employed both Ti-attached and Ti-substituted IrO_2_(110) surface models in density functional theory (DFT) calculations. These configurations represent simplified limiting cases of local coordination environments at the interface, enabling systematic evaluation of the electronic modulation of Ir sites induced by Ti species. While the “Ti-attached” model reflects surface-bound Ti species observed by TEM, the Ti-substituted model ("Ti-doped”) represents a more strongly coupled configuration that may arise from interfacial mixing during post-annealing (Supplementary Fig. 25). Accordingly, these models are intended to capture local electronic interactions rather than to reproduce the exact atomic structure of the catalyst under OER conditions, and therefore, the results should be interpreted as qualitative mechanistic insights. Based on these models, we first examined the electronic perturbation induced by Ti at the IrO_2_ surface. Attachment of a Ti atom to the IrO_2_(110) surface results in local charge redistribution around neighboring Ir sites (Fig. 4e). Bader charge analysis reveals that Ir atoms adjacent to Ti exhibit a substantially reduced charge (0.81 *e*) compared with both pristine IrO_2_ (Fig. 4d) and Ti-doped IrO_2_ models (Supplementary Fig. 26a), consistent with electron transfer across Ir-O-Ti linkages and an electronic downshift of Ir states (Fig. 4f)^41^. We further evaluated the thermodynamic implications of Ti incorporation in the model structures. Increasing Ti content in the IrO_2_ lattice raises the oxygen-vacancy formation energy and broadens the thermodynamically stable potential-pH window (Supplementary Fig. 26b, c). These findings indicate that interfacial Ti-O-Ir coupling stabilizes the oxygen sublattice, consistent with a reduced tendency toward lattice-oxygen participation during OER^26,28,29,44^. We note that the present XPS and oxygen-vacancy formation energy analyses primarily reflect enhanced lattice stability, and that a direct confirmation of suppressed lattice-oxygen participation would require isotope-labeling studies. Beyond this lattice stabilization, the lower baseline Ir valence state increases the potential required to access highly oxidized Ir species (Ir^5+^/Ir^6+^), which are associated with transient dissolution under strongly anodic conditions^13,14,22^. This electronic stabilization is corroborated by the absence of detectable Ir migration in post-mortem TEM analysis (Fig. 3d), lower dissolved Ir concentrations measured by ICP-MS and ICP-OES (Supplementary Figs. 15–19), and the minimal change in the Ir 4*f* binding energy observed by post-operation XPS (Supplementary Fig. 27). Overall, the Ti-induced electronic stabilization of Ir mitigates over-oxidation-driven dissolution under acidic OER conditions and provides the mechanistic foundation for the enhanced durability observed in the TiO_2_-modified anodes.

### Interfacial stabilization via enhanced ionomer-catalyst interactions

**Fig. 5:**
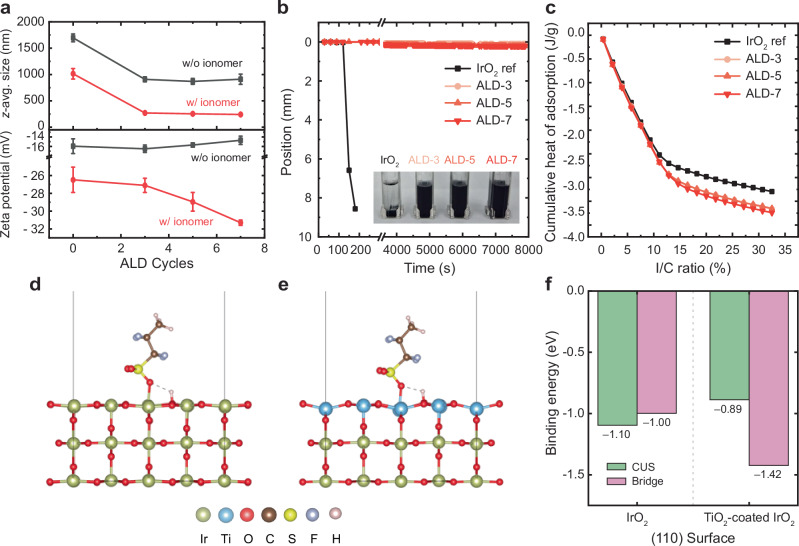
Colloidal stabilization and enhanced ionomer-catalyst interactions enabled by TiO_2_ coating. **a***z*-average hydrodynamic diameter (top) and zeta potential (bottom) of reference IrO_2_ and TiO_2_-coated IrO_2_ catalysts as a function of ALD cycle number, measured in the absence and presence of ionomer. TiO_2_ coating reduces aggregate size and yields more negative zeta potentials in ionomer-containing inks relative to bare IrO_2_, indicating enhanced electrostatic repulsion and dispersion stability. **b** Accelerated sedimentation profiles of catalyst inks measured at 4000 rpm. Reference IrO_2_ inks sedimented rapidly, whereas TiO_2_-coated IrO_2_ inks remained stable over extended timescales (inset: photographs taken after 8000 s). **c** Cumulative heat of ionomer adsorption on catalyst surfaces as a function of I/C ratio measured by ITC, showing higher adsorption heat for TiO_2_-coated IrO_2_ catalysts than for bare IrO_2_. **d**, **e** DFT-optimized structures (side view) of a -SO_3_H functional group of a PFSA ionomer adsorbed on the pristine IrO_2_(110) surface (**d**) and the TiO_2_-monolayer-coated IrO_2_(110) surface (**e**). **f** Calculated ionomer binding energies at CUS and bridge sites on pristine and TiO_2_-monolayer-coated IrO_2_(110) surfaces, showing a pronounced enhancement in binding strength at the bridge site upon TiO_2_ coating.

Having established the electronic basis for Ir stabilization, we next examined how TiO_2_ modification alters catalyst-ionomer interactions through colloidal characterization of catalyst inks. Dynamic light scattering (DLS) measurements show that the hydrodynamic diameter of bare IrO_2_ ink decreases sharply from 1697 to 908 nm only after three ALD cycles (Fig. 5a). A similar trend is observed in the presence of ionomer at an ionomer-to-catalyst (I/C) ratio of 0.2, where the average particle size decreases markedly from 1014 nm for bare IrO_2_ to 270 nm for ALD-3. This size reduction, even in the absence of ionomer, originates from TiO_2_-induced surface hydrophilicity that improves dispersion and demonstrates that only a few ALD cycles substantially modify surface properties. In contrast to the size evolution, the zeta potential changed marginally from –16.0 to  −14.7 mV in the absence of ionomer. Upon ionomer addition, however, the zeta potential becomes progressively more negative from  −26.5 to  −31.3 mV with increasing ALD cycle number, indicating enhanced adsorption of anionic sulfonate groups of the ionomer on the TiO_2_-modified surface. Accelerated sedimentation tests further show that the reference ink precipitated within 3 min with a sedimentation rate of 42 μm s^−1^, whereas all TiO_2_-modified inks remained stable for more than 2 h with sedimentation rates below 0.1 μm s^−1^ (Fig. 5b and Supplementary Fig. 28). These results confirm the markedly enhanced colloidal stability imparted by the TiO_2_ coating.

The thermodynamics of ionomer adsorption were quantified by isothermal titration calorimetry (ITC), which monitored the cumulative heat released during ionomer adsorption onto TiO_2_-coated catalysts (Fig. 5c and Supplementary Fig. 29). For the reference catalyst, the cumulative heat curve exhibits a clear inflection at an I/C ratio of  ~10%, beyond which the incremental heat release markedly diminishes, indicating early saturation of available adsorption sites. In contrast, all TiO_2_-coated catalysts sustained a near-linear decrease in cumulative heat up to an I/C ratio of  ~15%, demonstrating an extended adsorption regime prior to saturation. At I/C = 30%, the cumulative heat for TiO_2_-coated catalysts was approximately 0.4 J g^−1^ higher than that for bare IrO_2_, suggesting stronger interactions with the ionomer^45–47^. Complementary ^19^F nuclear magnetic resonance (NMR) analysis validated an increase in the adsorbed ionomer fraction from 25% to 45% for ALD-7 at I/C = 0.2 (Supplementary Fig. 30). These combined calorimetric and spectroscopic measurements establish that the TiO_2_ coating systematically enhances ionomer adsorption capacity and binding strength.

The origin of these enhanced interactions was rationalized through DFT calculations on the pristine IrO_2_(110) (Fig. 5d) and IrO_2_(110) coated with a monolayer of TiO_2_ (Fig. 5e) as simplified model surfaces. Sulfonate groups, which serve as anchoring moieties in perfluorosulfonic acid (PFSA) ionomers, exhibit moderate binding on pristine IrO_2_, with adsorption energies of  −1.10 eV at coordinatively unsaturated Ir sites (CUS) and  −1.00 eV at bridge sites. The TiO_2_ modification slightly weakens adsorption at CUS (−0.89 eV) but markedly strengthens binding at bridge sites (−1.42 eV) (Fig. 5f), resulting in a higher average binding energy than that of pristine IrO_2_(110). This binding enhancement originates from charge redistribution across Ti-O-Ir linkages, which creates electron-deficient Ti sites and lowers the Bader charge of adjacent Ir atoms. These electronic effects strengthen electrostatic stabilization of sulfonate groups, particularly at bridge sites (Supplementary Fig. 31 and Supplementary Data 1). By comparison, pristine IrO_2_ screens adsorbate-induced charge perturbations, and pristine TiO_2_(110) exhibits unfavorable sulfonate adsorption, indicating that the strong binding arises from interfacial electronic coupling with IrO_2_ rather than from TiO_2_ alone (Supplementary Fig. 32).

Collectively, the Ti-induced electronic modulation via interfacial Ti-O-Ir coupling not only suppresses Ir over-oxidation but also strengthens the electrostatic interaction with the ionomer, thereby stabilizing both the catalyst surface and catalyst-layer architecture and improving PEMWE durability. Previous studies have shown that strong sulfonate adsorption can block active sites on metallic Pt catalysts^48^. In contrast, in the present oxide-based system, ionomer interaction occurs primarily at the TiO_2_-modified interface and contributes to catalyst-layer stabilization, rather than direct active-site blocking. Although this study focuses on stabilization of rutile IrO_2_ catalysts, all experiments were also performed on amorphous IrO_x_ catalysts as a proof of concept, and the same stabilization strategy proved comparably effective (Supplementary Note 2, Supplementary Figs. 33–45 and Supplementary Tables 10, 11).

### Roll-to-roll compatibility and large-area processability

Finally, the enhanced colloidal stability of the TiO_2_-coated catalyst enabled direct translation of the materials design to industrially relevant roll-to-roll processing, yielding uniform, defect-free coatings with precise thickness control. This process compatibility is further supported by the fact that the TiO_2_ ALD step is applied to the catalyst powder prior to ink formulation, rendering it fully decoupled from the continuous coating process. In addition, the availability of ALD reactors with kilogram-scale powder throughput further underscores the scalability of the approach^49^.

**Fig. 6:**
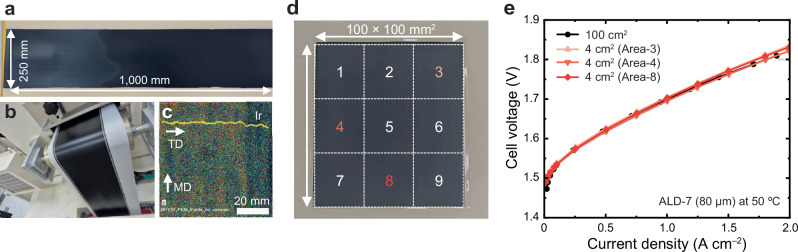
Meter-scale roll-to-roll manufacturing and large-area processability of TiO_2_ coating-based PEMWE systems. **a**, **b** Photographs of the continuous roll-to-roll coating process validating the scalability of the TiO_2_-coated catalyst architecture toward industrial manufacturing. **c** XRF spectral mapping of the Ir-L*α* line across a 100 cm^2^ MEA fabricated with ALD-7 by roll-to-roll processing. The map illustrates the spatial distribution of Ir loading along the MD and TD. **d** Photographic image of a 100  × 100 mm^2^ (100 cm^2^) MEA with the ALD-7 anode. The overlaid grid defines nine sub-regions (Areas 1–9) used for large-area spatial evaluation of catalyst loading and performance uniformity. **e** Polarization curves of PEMWE cells assembled with the full 100 cm^2^ MEA and representative 4 cm^2^ MEAs extracted from Areas 3, 4, and 8, obtained at 50 ^∘^C with an Ir loading of 0.4 mg cm^−2^. The nearly identical electrochemical responses confirm robust large-area scalability of the TiO_2_-ALD-based anode architecture.

A continuous web with a width of 250 mm and a length of  ~5 m was successfully produced (Fig. 6a, b), and used to fabricate a 100 cm^2^ MEA (100 mm × 100 mm) paired with an 80 μm membrane. X-ray fluorescence (XRF) mapping confirmed highly uniform Ir loading, with relative standard deviations of 1.12% along the machine direction (MD) and 2.74% along the transverse direction (TD) over the full 100 cm^2^ MEA (Fig. 6c and Supplementary Table 12). Spatially resolved analysis across nine sub-regions (Areas 1–9) further confirms highly uniform Ir loading (0.40  ± 0.04 mg cm^−2^) over the entire MEA (Fig. 6d and Supplementary Table 13). Polarization curves obtained from PEMWE cells at 50 ^∘^C assembled with the full 100 cm^2^ MEA and representative 4 cm^2^ MEAs extracted from Areas 3, 4, and 8 exhibit nearly identical electrochemical responses (Fig. 6e). These results demonstrate robust large-area reproducibility and uniform electrochemical performance across the electrode area, confirming the scalability of the TiO_2_-ALD-based anode architecture. Accordingly, the laboratory-scale stabilization concept translates directly to meter-scale production and is amenable to industrial adoption.

## Discussion

This work establishes a dual-interface stabilization strategy for PEMWE anodes through TiO_2_-ALD modification of IrO_2_ catalysts. At moderate ALD cycle numbers, the deposited Ti exists as interfacial Ti-O-Ir species that simultaneously modulate the electronic structure of the catalyst surface and the ionomer-catalyst interactions. This modulation suppresses Ir over-oxidation and dissolution while preserving the pore architecture of the catalyst layer and mass-transport pathways during high-current operation. The optimized anode sustained operation at 3.0 A cm^−2^ for 2600 h with a near-zero degradation rate at an Ir loading of 0.4 mg cm^−2^, thereby decoupling high-current operation from accelerated degradation without reliance on excessive noble-metal loading. Compatibility with roll-to-roll manufacturing further enabled translation of this material stabilization into system-level benefits. More broadly, this interfacial engineering strategy provides a generalizable design principle for durable, techno-economically viable PEMWE systems operating under industrially relevant conditions.

## Methods

### Preparation of TiO_2_-coated IrO_2_ catalysts

Commercial IrO_2_ powder (TEC77110, Tanaka Kikinzoku Kogyo) was used as the base catalyst for ALD of TiO_2_. TiO_2_ was deposited using tetrakis(dimethylamino)titanium (TDMAT, 99.9%, EG Chem) as the titanium precursor and deionized H_2_O (99.9%) as the oxidizing counter-reactant. The IrO_2_ powder loading ranged from 1.8 to 5.0 g per batch. The deposition was carried out in a commercial rotary ALD reactor (Atomic Shell, CN1) at 120 ^∘^C. Each ALD cycle consisted of a TDMAT exposure step (360–480 s), followed by an Ar purge (800–920 s), an H_2_O exposure step (2–8 s), and a second Ar purge (540–1080 s). The exposure and purge durations were adjusted depending on the IrO_2_ powder loading. During deposition, the chamber pressure was maintained at approximately 1.6 Torr with a total Ar carrier-gas flow rate of 150 sccm. Uniform precursor distribution and conformal coating were achieved by operating the reactor at a rotation speed between 5 and 30 rpm. After ALD deposition, TiO_2_-coated IrO_2_ catalysts were annealed in air at 400 ^∘^C for 1 h.

### Electrode coating and MEA fabrication

The catalyst ink was prepared by dispersing the TiO_2_-coated IrO_2_ powder in a mixed solvent system. Deionized water (18.2 MΩ cm, Milli-Q) and *n*-propyl alcohol (>99.5%, Daejung) were used as solvents, and a PFSA ionomer dispersion (D72-25BS, equivalent weight of 720 g mol^−1^, Aquivion) was employed. The ink composition was adjusted to a water-to-alcohol mass ratio of 50:50, a total solid content of 15 wt%, and an I/C ratio in the range of 0.10–0.20. Homogeneous dispersion was achieved by initial ultrasonication for 10 min, followed by ball milling at 230 rpm for 72 h using 3 mm zirconia balls.

Large-area electrodes were fabricated using a roll-to-roll slot-die coating system (Multicoater, HIRANO TECSEED). A precision slot-die head equipped with a 150 μm-thick shim plate was employed to define the fluid geometry. The effective coating width was set to 250 mm to deposit catalyst layers onto a polytetrafluoroethylene (PTFE) substrate (SANG-A FRONTEC). Uniform deposition was obtained by maintaining a coating speed of 1 m min^−1^ and an ink flow rate of 10 mL min^−1^. The wet electrode films were subsequently dried in a heating furnace maintained at 120 ^∘^C until complete evaporation of the solvent mixture was achieved.

The MEAs were fabricated by a hot-press decal transfer method using either a Nafion N115 membrane (127 μm, Chemours) or an M275.80 membrane (80 μm, Gore), both of which were used as received without any additional pretreatment. Catalyst-coated decals were transferred to the membrane by hot pressing at 140 ^∘^C under an applied force of 0.5 metric tons, corresponding to approximately 12.3 MPa.

The spatial distribution of Ir loading was characterized by micro-XRF (M4 TORNADO PLUS, Bruker). Measurements were conducted at an X-ray tube voltage of 50 kV and a current of 600 μA, with the detector dead time maintained below 60%. Elemental mapping was performed using a 20 μm spot size, 150 μm step interval, and 10 ms dwell time per pixel. Statistical metrics, including the mean, standard deviation, and relative standard deviation (RSD), were evaluated separately along the MD and TD based on the Ir-L*α* signal intensity. The RSD of catalyst loading across different positions was maintained below 5%, confirming homogeneous coating across the electrode area. Quantitative Ir loading values were obtained by converting the Ir-L*α* signal using a calibration curve. The resulting loadings correspond to 0.40 ± 0.04 mg_Ir_ cm^−2^ for the anode and 0.20 ± 0.02 mg_Pt_ cm^−2^ for the cathode.

### Characterization of TiO_2_-coated IrO_2_ catalysts and anodes

Coffee-ring analysis was performed by dispersing 0.01 g of catalyst in 10 mL of *n*-propanol (99.5%, Daejung). Subsequently, a 5 μL aliquot of the dispersion was deposited onto preheated glass substrates maintained at 80 ^∘^C and allowed to dry, forming a characteristic coffee-ring pattern. The resulting structures were characterized using a confocal laser scanning microscope (KEYENCE VK-200 series). Ring width and height were measured at a magnification of 100×. For each ring, measurements were taken at five randomly selected positions, and the reported values represent the average of three independent rings.

Surface and cross-sectional morphologies of electrodes were examined using a FE-SEM (JSM-7610F Plus, JEOL) operated at accelerating voltages in the range of 5–15 kV. Cross-sectional specimens were prepared by argon ion milling (ArBlade 5000, HITACHI). Milling was conducted at an Ar gas flow rate of 0.15 sccm, with an acceleration voltage of 6 kV and a discharge voltage of 1.5 kV.

TEM and STEM were performed using a JEM-ARM200F (JEOL) and a Talos F200X (Thermo Fisher Scientific) equipped with an EDS detector (JED-2300T Dual) operated at 200 kV. For cross-sectional TEM analysis of MEAs, samples were impregnated with epoxy resin at 25 ^∘^C and subsequently sectioned using an ultramicrotome (Leica, Germany).

The digital image-based 2D porosity of the MEA anodes was quantified from cross-sectional TEM images in accordance with ASTM E2109,^50^ using automated image analysis in ImageJ. TEM images acquired at 15,000× magnification were analyzed with up to ten representative images selected per sample. Electrode regions were cropped, converted to 8-bit grayscale, and thresholded to distinguish catalyst (dark) and pore (bright) regions. The pore area fraction was automatically calculated and reported as the mean value with the corresponding standard deviation.

The pore size distributions of the catalyst layers were determined by mercury intrusion porosimetry using an Autopore V 9600 (Micromeritics). Pore diameters were calculated from the mercury intrusion volume as a function of applied pressure based on the Washburn equation. Measurements were performed over a pressure range of 180.86–361,727.08 psia, corresponding to pore diameters below 1 μm.

### RDE-based evaluation of ECSA and intrinsic activity

Catalyst inks were prepared from 10 mg of catalyst, 300 μL of deionized water, 900 μL of *n*-propanol (99.5%, Daejung), and 5.2 μL of 20 wt% Nafion (D2020, Chemours). The inks were ultrasonicated for 20 min, and 5.5 μL was drop-cast onto a glassy carbon electrode with a diameter of 5 mm, corresponding to an Ir loading of 0.2 mg cm^−2^. Electrochemical measurements were performed using a potentiostat (ZIVE MP2, WonATech) coupled with an RDE system (RRDE-3A, ALS) at a rotation rate of 1600 rpm with an Ag/AgCl (3 M KCl) reference electrode and a Pt counter electrode in 80 mL of 0.5 M H_2_SO_4_ (Daejung). The reference electrode was used without additional calibration, and potentials were converted to the reversible hydrogen electrode (RHE) scale by adding 0.2271 V, calculated from *E*_RHE_ = *E*_Ag/AgCl_ + 0.210 + 0.0591 × pH, using an electrolyte pH of 0.29. The double-layer capacitance (*C*_dl_) was obtained from cyclic voltammetry (CV, 0.2–1.2 V, 5–100 mV s^−1^) with *Δj* extracted at 1.1 V, and *C*_dl_ was determined from the slope of *Δj* as a function of scan rate. ECSA was calculated according to ECSA = *C*_dl_/*C*_s_, using a specific capacitance (*C*_s_) of 40 μF cm^−2^. Electrochemical impedance spectroscopy (EIS, 1.4 V, 100 kHz–100 Hz) and linear sweep voltammetry (LSV, 1.1–1.6 V, 5 mV s^−1^) were performed once to evaluate iR-corrected potentials.

### Three-electrode durability evaluation and Ir dissolution analysis

Catalyst inks were prepared by dispersing 20 mg of TiO_2_-coated IrO_2_ catalyst in a mixed solvent of deionized water and isopropyl alcohol (99.5%, Daejung) (1:1 v/v) with 80 μL of 5 wt% Nafion (D2020, Chemours), followed by ball milling for 3 days. The inks were drop-cast (48 μL) onto microporous-layer-coated carbon paper (SIGRACET 36BB) over a defined area of 1 cm  × 1 cm, dried, and thermally treated at 80 ^∘^C for 5 min, yielding an Ir loading of 0.4 mg cm^−2^. Electrochemical measurements were performed using a potentiostat (CS310M, CORRTEST) in a three-electrode configuration with an Ag/AgCl (3 M KCl) reference electrode, a Pt counter electrode, and 80 mL of 0.5 M H_2_SO_4_ electrolyte (Daejung) under continuous stirring. The reference electrode was used without additional calibration, and potentials were converted to the RHE scale by adding 0.2271 V, using an electrolyte pH of 0.29. The accelerated stress test (AST) protocol included conditioning CV (1.0 to 1.6 V, 100 mV s^−1^, 3 cycles), EIS (1.3 V, 100 kHz to 100 mHz), and LSV measurements (1.0 to 2.0 V, 50 mV s^−1^), followed by accelerated cycling between 1.3 and 1.6 V at 200 mV s^−1^ for 40,000 cycles. The iR-corrected potential at 100 mA cm^−2^ was extracted from periodic LSV measurements using the ohmic resistance obtained from EIS. Electrolyte samples were collected at selected cycle intervals and analyzed by ICP-OES to quantify the cumulative dissolution of Ir. For chronopotentiometry (CP) durability tests, galvanostatic operation at 10 mA cm^−2^ was applied up to 48 h with intermittent electrochemical diagnostics and electrolyte sampling using the same procedure.

### PEMWE single-cell assembly and electrochemical measurements

The electrochemical performance of the fabricated MEAs was evaluated using a single-cell PEMWE with an active area of 4 cm^2^. A Pt-coated titanium porous transport layer (2GDL10N-026 BS02PT, Bekaert) was used at the anode, while a carbon fiber gas diffusion layer (E35H, Freudenberg) was employed at the cathode. The cell was assembled using titanium bipolar plates incorporating single-serpentine flow fields. Glass-fiber-reinforced PTFE gaskets ensured uniform contact pressure and sealing, and the cell was tightened to a torque of 4.9 N m.

During operation, deionized water (18.2 MΩ cm) was preheated and supplied to the anode at a flow rate of 30 mL min^−1^, whereas no water was fed to the cathode. The cell temperature was maintained at 80 ^∘^C throughout the experiments and was monitored in real time using a thermocouple. As an exception, the reproducibility test of the 100 cm^2^ MEA was conducted at 50 ^∘^C using a Ti-based porous transport layer.

All electrochemical measurements were performed using a potentiostat (SHP1003, WonATech). Before performance evaluation, the catalyst layers were activated using a pre-conditioning protocol that consisted of CV for at least 10 cycles between 0.05 and 1.6 V, followed by a galvanostatic hold at 1.5 A cm^−2^ for 10 min. After conditioning, polarization curves were obtained using a stepwise galvanostatic protocol. In this protocol, the current density was increased in discrete increments and was held for 5 min at each step to achieve steady-state conditions. EIS measurements were subsequently conducted over a frequency range from 10 kHz to 100 mHz, and the resulting high-frequency resistance (HFR) was used to obtain iR-free cell voltages from the polarization data.

Long-term durability tests were conducted using a cyclic protocol consisting of galvanostatic operation at 3.0 A cm^−2^ and a cell temperature of 80 ^∘^C for 138 h, followed by a 24 h rest period and subsequent performance evaluation for approximately 6 h using stepwise polarization. Each cycle required approximately one week and was repeated multiple times. Comprehensive electrochemical characterization, including polarization curves and EIS measurements, was performed once at both the BoT and the EoT. Intermittent polarization curves were additionally recorded approximately once per week during operation. The degradation rate was determined from the voltage change at 3.0 A cm^−2^ extracted from these polarization measurements.

### Polarization curve decomposition and overpotential analysis

Polarization curves were decomposed into kinetic (*η*_kin_), ohmic (*η*_ohm_), and mass-transport (*η*_mt_) components. Although the theoretical reversible potential is approximately 1.17 V at 80 ^∘^C, the measured onset potential (*E*_onset_ ~ 1.43 V) was used as the reference for evaluation of practical overpotential contributions. Accordingly, the operating cell voltage (*E*_cell_) can be expressed as:

1 $${E}_{{{{\rm{cell}}}}}={E}_{{{{\rm{onset}}}}}+{\eta }_{{{{\rm{kin}}}}}+{\eta }_{{{{\rm{ohm}}}}}+{\eta }_{{{{\rm{mt}}}}}$$

The ohmic overpotential (*η*_ohm_) was calculated from the HFR (*R*_HFR_) obtained from EIS measurements according to *η*_ohm_ = *j* × *R*_HFR_, where *j* denotes the current density. The kinetic overpotential (*η*_kin_) was determined by fitting the iR-free polarization data to the Tafel equation, ${\eta }_{{{{\rm{kin}}}}}=a+b\log (j)$. The fitting procedure was applied in the low-current-density region of 0.01–0.1 A cm^−2^, where cell behavior is predominantly governed by activation-controlled kinetics rather than transport limitations. The mass-transport overpotential (*η*_mt_) was determined by subtracting the onset potential and the calculated kinetic and ohmic overpotentials from the total measured cell voltage according to *η*_mt_ = *E*_cell_ − *E*_onset_ − *η*_kin_ − *η*_ohm_.

### Electronic structure characterization of TiO_2_-modified IrO_2_ catalysts

XPS measurements were performed using a K-Alpha+ spectrometer (Thermo Fisher Scientific) with a monochromatic Al K*α* X-ray source (1486.6 eV). Binding energies were calibrated using the −CF_2_ reference peak. Quantitative analysis was conducted using Avantage software (v5.992) with a Smart (Shirley-type) background and mixed Gaussian-Lorentzian line shapes. The Ir 4*f* doublets were modeled with a fixed spin-orbit splitting of  ~3.0 eV and a 4:3 area ratio. Spectral overlap between the Ir 4*f* and Ti 3*s* regions was addressed by peak deconvolution, and the Ti 3*s* contribution was estimated from the Ti 2*p* region to enable accurate quantification of the Ir 4*f* signal.

Ir L_3_-edge XANES and EXAFS measurements were performed at the 8C beamline of the Pohang Light Source-II (PLS-II) at the Pohang Accelerator Laboratory. Spectra were collected using stepwise energy scanning, with finer energy steps around the absorption edge and larger steps in the extended EXAFS region. XANES and Fourier-transformed EXAFS data were analyzed using the Athena software package following standard normalization and background subtraction procedures.

### Characterization of ionomer-catalyst interactions

Hydrodynamic particle size and zeta potential measurements were performed using a Zetasizer (Malvern Panalytical). Catalyst inks were diluted in deionized water containing 10 mM NaCl (99%, Daejung) to obtain a catalyst concentration of 0.2–0.3 mg mL^−1^. For each sample, 20 measurements were collected per run, and the measurements were repeated three times. The reported values represent the averages with the corresponding standard deviations.

Accelerated sedimentation tests were carried out using an analytical centrifuge (LUMiSizer 6512-17, LUM GmbH) equipped with an 865 nm light source. Samples were loaded into rectangular synthetic cells (LUM 10 mm PA) and measured at 25 ^∘^C with a rotation speed of 4000 rpm (2160 ×g). Transmission profiles were recorded every 30 s over a total duration of 8000 s. Data processing included normalization, dynamic baseline correction, and moving-average smoothing (9 points).

ITC measurements were performed using a MicroCal PEAQ (Malvern Panalytical). The catalyst dispersion (1 mg mL^−1^) was prepared in deionized water and loaded into the sample cell (280 μL), while the ionomer solution (2.5 mg mL^−1^) in deionized water was placed in the syringe. The titration protocol consisted of 19 injections, including an initial injection volume of 0.4 μL followed by 18 injections of 2 μL each. Injections were applied with a spacing of 120 s and a duration of 4.0 s under continuous stirring at 750 rpm, with a reference power of 5.0 μcal s^−1^. The raw thermograms were analyzed by curve fitting using a one-site binding model.

A quantitative analysis of ionomer adsorption was conducted using ^19^F NMR spectroscopy ^51,52^. A calibration curve was first established by preparing ionomer solutions with concentrations ranging from 0.25 to 8 mM and measuring the corresponding ^19^F NMR spectra. Quantification was performed using the characteristic  −CF_3_ resonance at approximately –80 ppm. Based on this calibration, catalyst dispersions were prepared using bare IrO_2_ reference and ALD-7 catalysts, with ionomer added at different concentrations: 1.0, 1.5, and 2.0 mM. The dispersions were centrifuged at 16,000 rpm (21,500 ×g) for 2 h to fully sediment the catalyst particles along with any adsorbed ionomer. The resulting supernatants were collected and analyzed by ^19^F NMR to quantify the residual, non-adsorbed ionomer. The amount of ionomer adsorbed onto the catalyst was calculated as the difference between the initially added ionomer and the amount detected in the supernatant.

### DFT calculations

All DFT calculations were performed using the Vienna ab initio Simulation Package (VASP)^53–56^ with the projector-augmented wave (PAW) method. The generalized gradient approximation with the Perdew-Burke-Ernzerhof functional was adopted as the exchange-correlation treatment^57^. The electron-ion interactions were described using PAW potentials that explicitly included semi-core electrons for both iridium (5*d*^8^6*s*^1^) and titanium (3*p*^6^3*d*^2^4*s*^2^), while oxygen atoms were treated with their ground-state valence configuration (2*s*^2^2*p*^4^).

A three-layer IrO_2_ slab exposing the (110) surface was constructed. A vacuum region of 20 Å was introduced along the *z*-direction to avoid inter-slab interactions. Geometry optimizations were carried out using the conjugate gradient algorithm. A plane-wave energy cutoff of 500 eV and a *Γ* centered 3 × 3 × 1 **k**-point mesh were employed. The electronic and ionic convergence criteria were set to 10^−6^ eV for total energy and 0.01 eV Å^−1^ for atomic forces, respectively. The Hubbard correction (DFT + *U*) was applied to Ti 3*d* states to account for on-site Coulomb interactions, with a *U* value of 4.2 eV^58^.

Atomic charge states were analyzed using the grid-based Bader charge analysis method^59^. The all-electron charge density was reconstructed by combining valence and core charge densities, and the atomic charge of each element was defined as the difference between the number of electrons in the neutral atom and the integrated Bader charge.

The monomeric unit of the PFSA ionomer consists of a CF_*x*_ backbone and a side chain terminated with a sulfonic acid group. For adsorption energy calculations, the ionomer was represented by a molecular fragment corresponding to its side chain. The adsorption energy (*E*_ads_) was calculated as:

2 $${E}_{{{{\rm{ads}}}}}\,({{{\rm{eV}}}})={E}_{{{{\rm{slab+mol}}}}}-{E}_{{{{\rm{slab}}}}}-{E}_{{{{\rm{mol}}}}}$$

where *E*_slab+mol_ is the total energy of the adsorption system, *E*_slab_ is the energy of the clean (110) surface, and *E*_mol_ is the energy of the isolated neutral adsorbate molecule. A negative *E*_ads_ indicates thermodynamically favorable binding.

The Pourbaix diagrams were constructed using the module implemented in the *pymatgen* library^60–63^. The equilibrium redox reaction was expressed as follows:

3 $$\,{\mbox{Reactants}}\,+{{{{\rm{H}}}}}_{2}{{{\rm{O}}}}\rightleftharpoons \,{\mbox{Products}}\,+m{H}^{+}+n{e}^{-}$$

The reaction Gibbs free energy was related to the applied electrode potential (*E*) through the Nernst equation:

4 $$-nFE=\Delta {G}_{{{{\rm{rxn}}}}}=\Delta {G}_{{{{\rm{rxn}}}}}^{\circ }+2.303RT\log \frac{{a}_{{{{\rm{Products}}}}}}{{a}_{{{{\rm{Reactants}}}}}}-2.303RT\,m\,{{{\rm{pH}}}},$$

where *F* denotes the Faraday constant, *R* denotes the gas constant, *T* denotes the temperature, and *a* denotes the activity. The electrochemical potentials were referenced to the standard hydrogen electrode (SHE)^64^. The thermodynamically stable species at each potential-pH condition were identified by minimizing *ΔG*_rxn_ + *nFE*. The chemical potentials of reference solids under standard conditions were expressed as:

5 $${\mu }^{\circ }={H}^{\circ }-T{S}^{\circ }={E}_{{{{\rm{DFT}}}}}+{E}_{{{{\rm{ZPE}}}}}+\delta H-T{S}^{\circ },$$

where *E*_DFT_ represents the total energy obtained from DFT calculations, *E*_ZPE_ represents the zero-point energy, *δH* represents the integrated heat capacity from 0 to 298.15 K, and (*TS*)^∘^ represents the entropy contribution under standard conditions. For solid-phase species, the entropy contribution was neglected. The *E*_ZPE_ and *δH* terms for both solid and gas species were also neglected based on the assumption that these contributions largely canceled out between different species. Thermodynamic properties for aqueous ionic species were collected from previous studies^65–67^.

## Supplementary information

Supplementary Information

Description of Additional Supplementary Files

Supplementary Data 1

Transparent Peer Review file

## Source data

Source Data

## Data Availability

The data supporting the findings of this study are included in the Article and its Supplementary Information. All other supporting data are available from the corresponding authors upon request. Source data are provided in this paper.
