# Supplementary material for: Dual-interface stabilization of low-iridium anodes for durable proton exchange membrane water electrolysis
Source: Nat Commun. 2026 Jul 4;17:6818. doi: 10.1038/s41467-026-75113-6 (PMC13389522; doi:10.1038/s41467-026-75113-6)
Supplement: Supplementary file 1 — Supplementary Information [file 41467_2026_75113_MOESM1_ESM.pdf]

Supplementary Information for

Dual-interface stabilization of low-iridium anodes for durable proton exchange membrane water electrolysis

Eui Tae Kim,[1] Sangwoo Kim,[1] Sung-Eun Park,[1] Pumsuk Park,[1] Eunbyeol Ko,[1] Jemee Joe,[1] Ho Yeon Son,[1] Juyeon Kang,[1] Julie Junesoo Kim,[2] Kibeom Cheon,[2] Kyungin Kim,[2] Soree Kim,[1] Geunsung Lee,[1] Jaehak Jeong,[1] Manki Cho,[1] Noma Kim,[1] Jai Hyun Koh,[3,*] and Kihwan Kim[1,*]

[1]Platform Technology Research Center, LG Chem, Seoul 07796, Republic of Korea

[2]Analytical Sciences Center, LG Chem, Seoul 07796, Republic of Korea

[3]Clean Energy Research Center, Korea Institute of Science and Technology (KIST), Seoul 02792, Republic of Korea

Corresponding Authors

*Email: jhkoh@kist.re.kr (J.H.K.); kihwankim@lgchem.com (K.K.)

Table of Contents

1. Supplementary Notes

Note 1 | Benchmarking PEMWE durability from recent literature 5
Note 2 | Dual-interface stabilization of amorphous IrO_x by TiO_2-ALD coating.................... 6

2. Supplementary Figures

Fig. 1 | TEM images and STEM–EDS elemental maps of TiO_2-coated IrO_2 7
Fig. 2 | XPS analysis of TiO_2-coated IrO_2 catalysts 8
Fig. 3 | BET surface area analysis of TiO_2-coated IrO_2 8
Fig. 4 | XRD analysis of phase evolution in TiO_2-coated IrO_2 9
Fig. 5 | DSC thermal analysis of TiO_2-coated IrO_2 9
Fig. 6 | Compositional analysis as a function of TiO_2-ALD cycle number 10
Fig. 7 | Coffee-ring analysis of surface properties of TiO_2-coated IrO_2 10
Fig. 8 | Drop shape analysis of TiO_2-coated IrO_2 model films 11
Fig. 9 | Surface SEM analysis of TiO_2-coated IrO_2 anodes (ALD-7) anodes 12
Fig. 10 | AFM measurements of ALD-7 anodes 12
Fig. 11 | Drop shape analysis of ALD-7 anodes 13
Fig. 12 | RDE-derived intrinsic activity with benchmarking against state-of-the-art catalysts .. 14
Fig. 13 | Long-term stability and polarization analysis of TiO_2-coated IrO_2 anodes 15
Fig. 14 | Deconvolution of the total overpotential at the BoT and EoT 16
Fig. 15 | Ir dissolution during PEMWE operation measured by ICP-MS 17
Fig. 16 | Three-electrode AST and Ir dissolution analysis of TiO_2-coated IrO_2 17
Fig. 17 | LSV curves at various cycle intervals during three-electrode AST 18
Fig. 18 | Three-electrode CP evaluation and Ir dissolution analysis 19
Fig. 19 | LSV curves at various time intervals during three-electrode CP 19
Fig. 20 | Post-mortem surface and cross-sectional SEM analysis of ALD-7 anode 20
Fig. 21 | Post-mortem cross-sectional TEM images used for quantitative porosity analysis 21
Fig. 22 | Correlation between 2D porosity and mass-transport overpotential of anodes 22
Fig. 23 | XPS analysis of TiO_2-coated IrO_2 anodes 22
Fig. 24 | XANES analysis of TiO_2-coated IrO_2 catalysts 23
Fig. 25 | Optimized atomic structures of the Ti-attached and Ti-doped IrO_2(110) surface models 23
Fig. 26 | DFT investigation of Ti-incorporated IrO_2 24
Fig. 27 | XPS Ir 4*f* spectra of ALD-7 anodes after operation at 3.0 A cm^{-2} 25
Fig. 28 | Analytical centrifugation assessment of catalyst ink dispersion stability 26
Fig. 29 | ITC analysis of ionomer adsorption on TiO_2-coated IrO_2 27
Fig. 30 | Quantification of ionomer adsorption by ^{19}F NMR spectroscopy 28
Fig. 31 | DFT-calculated interfacial charge distribution upon ionomer adsorption 29
Fig. 32 | DFT investigation of ionomer adsorption on pristine TiO_2 29
Fig. 33 | TEM and STEM-EDS of TiO_2-coated amorphous-IrO_x (a-IrO_x@TiO_2) 30
Fig. 34 | BET surface area analysis of a-IrO_x@TiO_2 30
Fig. 35 | XRD analysis of phase evolution in a-IrO_x@TiO_2 31
Fig. 36 | DSC thermal analysis of a-IrO_x@TiO_2 31
Fig. 37 | Compositional analysis as a function of ALD cycle number for a-IrO_x@TiO_2 32
Fig. 38 | AFM measurements of a-IrO_x@TiO_2 anode 32
Fig. 39 | Drop shape analysis of a-IrO_x@TiO_2 anode 33
Fig. 40 | Long-term stability and polarization analysis of a-IrO_x@TiO_2 anode 33
Fig. 41 | Post-mortem analysis of a-IrO_x@TiO_2 anode 34
Fig. 42 | Three-electrode durability and Ir dissolution analysis of a-IrO_x@TiO_2 35
Fig. 43 | XPS analysis of a-IrO_x@TiO_2 36
Fig. 44 | XANES analysis of a-IrO_x@TiO_2 36
Fig. 45 | Colloidal stability and surface charge of a-IrO_x@TiO_2 37

3. Supplementary Tables

Table 1 | BET surface area and pore analysis of IrO_2 reference and ALD-7 catalysts. 38
Table 2 | Mercury intrusion porosimetry for IrO_2 reference and ALD-7 anodes. 38
Table 3 | AFM measurements for IrO_2 reference and TiO_2-coated IrO_2 anodes. 38
Table 4 | ECSA-normalized activity of TiO_2-coated IrO_2 catalysts. 39
Table 5 | Benchmarking ECSA-normalized activity of Ir-based catalysts. 39
Table 6 | Benchmarking PEMWE durability under record-high cumulative charge. 40
Table 7 | Electrochemical metrics for IrO_2 reference and ALD-7 anodes at 3.0 A cm^{-2}. 41
Table 8 | Quantitative comparison of anode properties and mass-transport overpotential. 41
Table 9 | Correlation between 2D porosity with mass-transport overpotential of anodes. 41
Table 10 | BET surface area and pore analysis of IrO_x reference and a-ALD-3 catalysts. 41
Table 11 | AFM measurements for IrO_x reference and a-ALD-3 anodes. 41
Table 12 | Statistical metrics of Ir loading from X-ray fluorescence (XRF) mapping across the 100 cm^2 membrane electrode assembly (MEA)... 42
Table 13 | XRF-measured Ir loading across nine sub-regions of the 100 cm^2 MEA.............. 42

Supplementary Note 1 | Benchmarking PEMWE durability from recent literature

A literature survey was conducted to benchmark proton-exchange membrane water electrolyzer (PEMWE) anode durability in a consistent and quantitative manner. Recent reports describing durability improvements of Ir-based anodes under galvanostatic operation were surveyed. Only studies that employed constant-current durability tests at current densities $\geq$1 A cm^{-2} and that achieved a cumulative charge density ($j \cdot t$) exceeding 1,000 A h cm^{-2} were considered, thereby excluding low-severity tests not representative of practical PEMWE operation. Studies that lacked a clearly defined degradation rate or that employed non-galvanostatic protocols, including variable-current operation or accelerated stress tests, were excluded. Based on these criteria, 43 literature reports were retained and benchmarked together with the present work (Supplementary Table 6).[1–43]

From each study, four key parameters were extracted: (1) Ir loading (mg cm^{-2}), (2) applied current density (A cm^{-2}), (3) test duration (h), and (4) voltage degradation rate (mV kh^{-1}). Two additional metrics were defined. The cumulative charge density ($j \cdot t$), also referred to as charge throughput, was used to quantify the severity of the durability test conditions, while normalization of this quantity by Ir loading yielded an Ir-normalized charge metric, which reflected Ir utilization efficiency under high-current operation.

The present work uniquely combines the highest cumulative charge with one of the lowest degradation rates among the surveyed studies. Furthermore, among high-current PEMWE reports, the present work achieves the highest Ir-normalized charge, which indicates superior Ir utilization efficiency.

Supplementary Note 2 | Dual-interface stabilization of amorphous IrO_x by TiO_2-ALD coating

The generality of the TiO_2-ALD stabilization strategy beyond crystalline systems was evaluated using amorphous iridium oxide (a-IrO_x) catalysts coated with TiO_2. Amorphous IrO_x is intrinsically less stable than rutile IrO_2 and exhibits a higher propensity for Ir dissolution under anodic potentials due to its disordered structure and abundance of undercoordinated sites.[41,44,45] Despite this intrinsic instability, TiO_2-coated a-IrO_x catalysts displayed markedly improved durability, approaching that of crystalline IrO_2. These results demonstrate the broad applicability of the dual-interface stabilization strategy across both crystalline and amorphous Ir-based oxygen evolution reaction (OER) catalysts.

Transmission electron microscopy (TEM) and scanning transmission electron microscopy–energy-dispersive X-ray spectroscopy (STEM–EDS) analyses confirm the formation of uniform and conformal TiO_2 coatings on the a-IrO_x surface (Supplementary Fig. 33). Braunauer–Emmett–Teller (BET) surface area analysis reveals that deposition of only three ALD cycles leads to a pronounced reduction in specific surface area from 93 to 41 $m^2\ g^{-1}$ (Supplementary Fig. 34 and Supplementary Table 10). X-ray diffraction (XRD) and differential scanning calorimetry (DSC) show that TiO_2-ALD coating and subsequent heat treatment induce partial crystallization of TiO_2 and IrO_2 while preserving the predominantly amorphous structure (Supplementary Figs. 35 and 36). X-ray photoelectron spectroscopy (XPS) and inductively coupled plasma optical emission spectrometry (ICP–OES) analyses verify linear and well-controlled growth of the TiO_2 coating with ALD cycle number (Supplementary Fig. 37). Atomic force microscopy (AFM) adhesion mapping and wettability measurements further reveal enhanced ionomer affinity and a transition toward more hydrophilic and aerophobic surface characteristics upon TiO_2 coating (Supplementary Figs. 38 and 39). These surface characteristics promote improved mass transport under high-current operation, consistent with trends observed for rutile IrO_2.

Electrochemical testing confirms substantially enhanced durability. Under PEMWE operation at 3.0 $A\,cm^{-2}$, the TiO_2-coated a-IrO_x catalyst with 3 ALD cycles (a-ALD-3) maintains stable performance with a near-zero degradation for more than 1,100 h, whereas the a-IrO_x reference shows rapid voltage decay (Supplementary Fig. 40). Post-mortem cross-sectional TEM reveals severe structural degradation and Ir migration into the membrane for the reference electrode, while the a-ALD-3 electrode remains structurally intact with no detectable Ir species after prolonged operation (Supplementary Fig. 41). Three-electrode measurements further confirm effective suppression of Ir dissolution under both cyclic and steady-state conditions (Supplementary Fig. 42).

Spectroscopic analyses indicate systematic electronic modulation of a-IrO_x by TiO_2 coating. XPS, X-ray absorption near edge spectroscopy (XANES), and extended X-ray absorption fine structure (EXAFS) results show reduced Ir 4*f* binding energies, decreased white-line intensity, and lowered Ir−O coordination with increasing ALD cycle number, consistent with suppressed Ir over-oxidation and reduced Ir dissolution (Supplementary Figs. 43 and 44). These trends mirror those observed for rutile IrO_2, with larger modulation amplitudes attributed to the intrinsically less stable surface of a-IrO_x. In addition, *z*-average hydrodynamic diameter and zeta potential measurements demonstrate improved colloidal stability arising from strengthened ionomer–catalyst interactions, consistent with behavior observed for rutile IrO_2 (Supplementary Fig. 45).

Overall, these results confirm that TiO_2 coating provides robust electronic and interfacial stabilization, suppressing Ir dissolution and preserving electrode microstructure not only for crystalline IrO_2 but also for amorphous IrO_x, highlighting the versatility of this strategy for durable PEMWE anode catalysts.

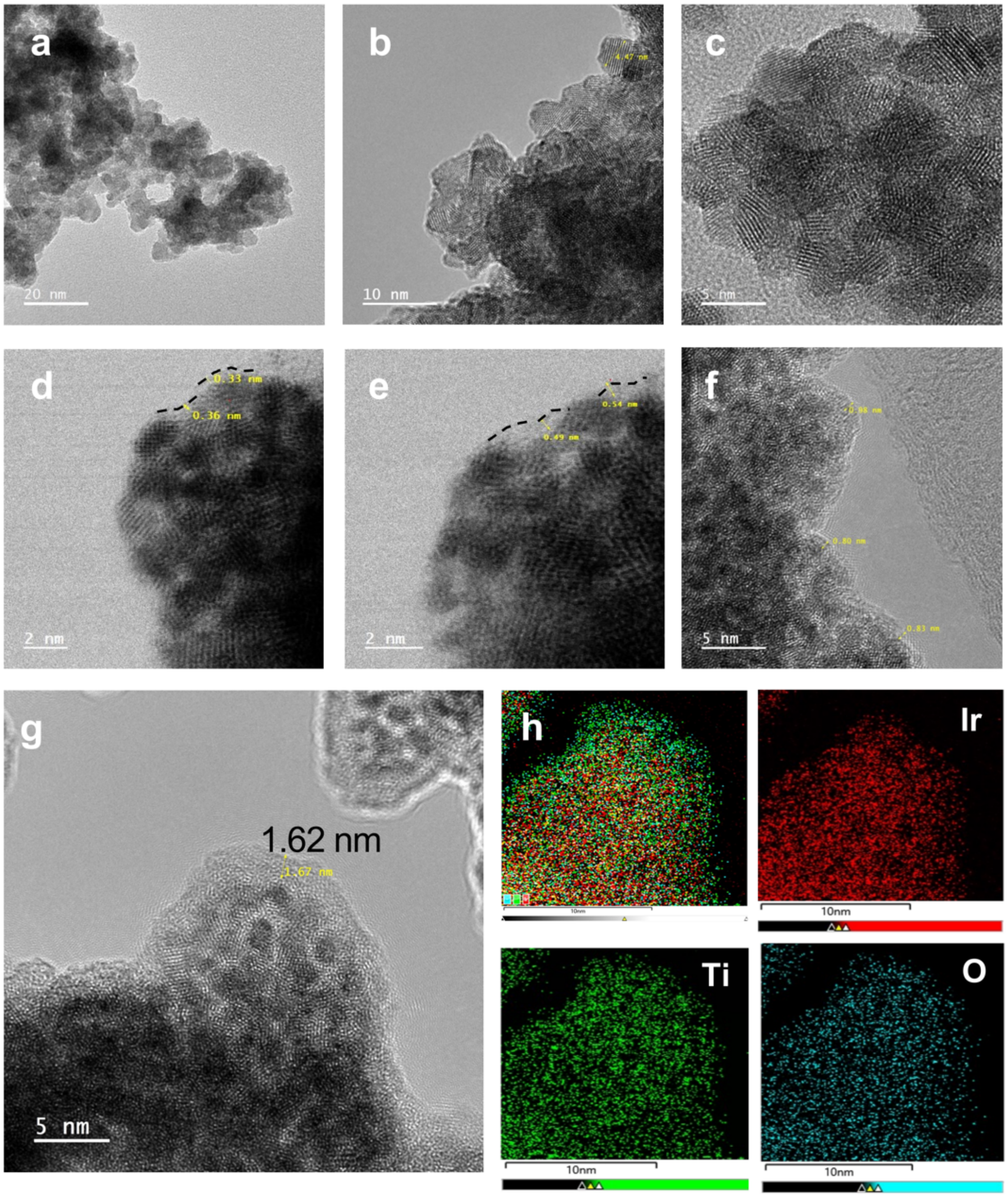

Supplementary Fig. 1 | TEM images and STEM–EDS elemental maps of TiO_2-coated IrO_2. a–c, High-resolution TEM images of the pristine IrO_2 reference catalyst at increasing magnifications. **d–g,** TEM images showing the progressive growth of a TiO_2 coating layer on IrO_2 catalysts with increasing atomic layer deposition (ALD) cycles: 5 cycles (ALD-5) **(d)**; 7 cycles (ALD-7) **(e)**; 15 cycles (ALD-15) **(f)**; and 30 cycles (ALD-30) **(g)**. Dashed lines and yellow annotations denote the measured thickness of the TiO_2 coating, which increases with ALD cycle number and reaches a maximum of approximately 1.62 nm for the ALD-30 sample. **h,** STEM–EDS elemental mapping of the ALD-30 catalyst. Maps of Ir (red), Ti (green), and O (cyan) confirm the conformal and spatially uniform distribution of the TiO_2 coating layer surrounding the IrO_2 catalyst particles.

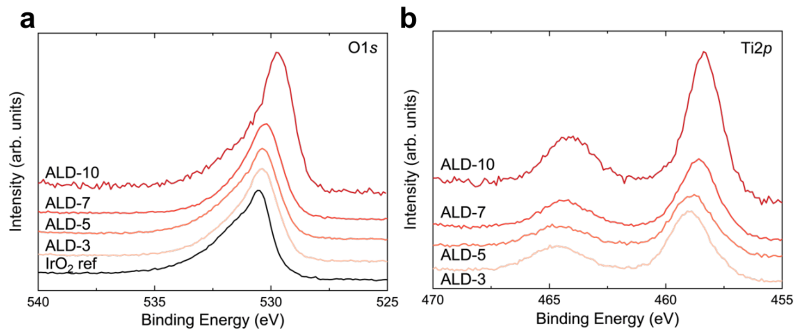

Supplementary Fig. 2 | XPS analysis of TiO_2-coated IrO_2 catalysts. a, b, High-resolution XPS narrow scans of the O 1*s* (**a**) and Ti 2*p* (**b**) regions for the IrO_2 reference and catalysts coated with 3–10 ALD cycles. Ti $2p_{3/2}$ binding energies of 458.5–459.0 eV are characteristic of Ti^{4+} states and consistent with TiO_2-like oxide environments. With increasing ALD cycle number, the O 1*s* lattice oxygen contribution and the Ti 2*p* signal intensity progressively increase, consistent with greater surface coverage by TiO_2.

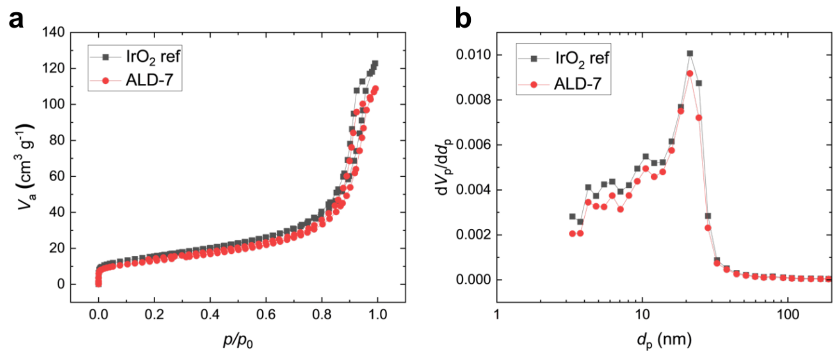

Supplementary Fig. 3 | BET surface area analysis of TiO_2-coated IrO_2. a, N_2 adsorption–desorption isotherms for the IrO_2 reference (black squares) and the ALD-7 catalyst (red circles). **b,** Corresponding Barrett–Joyner–Halenda (BJH) pore size distributions derived from the desorption branches show a modest decrease in adsorbed volume for the ALD-7 catalyst, consistent with partial coverage of the IrO_2 surface and mesopores by the TiO_2 coating rather than pore blockage. Quantitative BET surface areas and pore volumes are summarized in Supplementary Table 1.

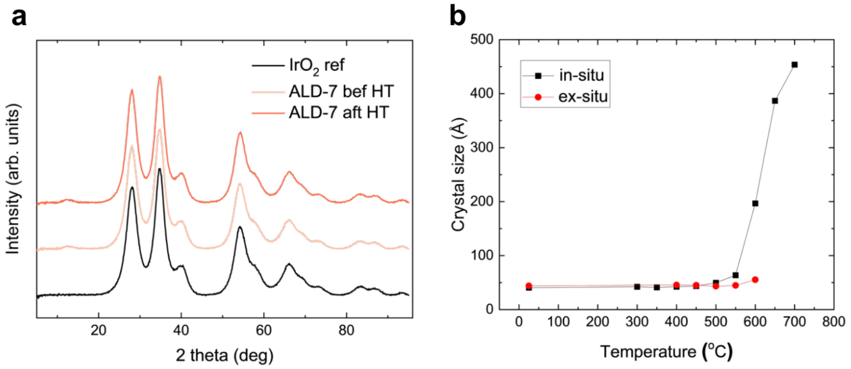

Supplementary Fig. 4 | XRD analysis of phase evolution in TiO_2-coated IrO_2. a, XRD patterns of the IrO_2 reference and the ALD-7 catalyst recorded before and after heat treatment, confirming preservation of the rutile IrO_2 phase without detectable phase transformation upon TiO_2 coating or subsequent thermal treatment. **b,** Evolution of the IrO_2 grain size of the ALD-7 as a function of heat-treatment temperature, comparing in-situ (black squares) and ex-situ (red circles) measurements. Grain sizes were estimated using the Scherrer equation, showing minimal grain growth up to ∼500 ºC followed by rapid coarsening at higher temperatures in the in-situ measurements, whereas ex-situ values remain comparatively stable.

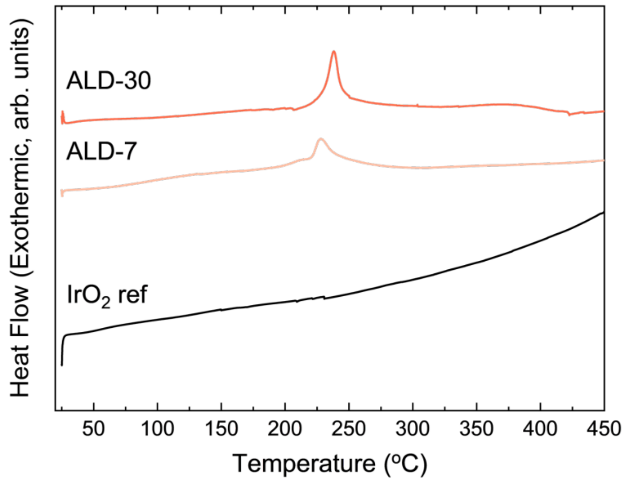

Supplementary Fig. 5 | DSC thermal analysis of TiO_2-coated IrO_2. DSC thermograms of the IrO_2 reference, ALD-7, and ALD-30 catalysts. Distinct exothermic features appearing between ∼200 and 250 ºC for the TiO_2-coated catalysts are attributed to crystallization of the TiO_2 coating layer, whereas the IrO_2 reference shows no corresponding transition over this temperature range.

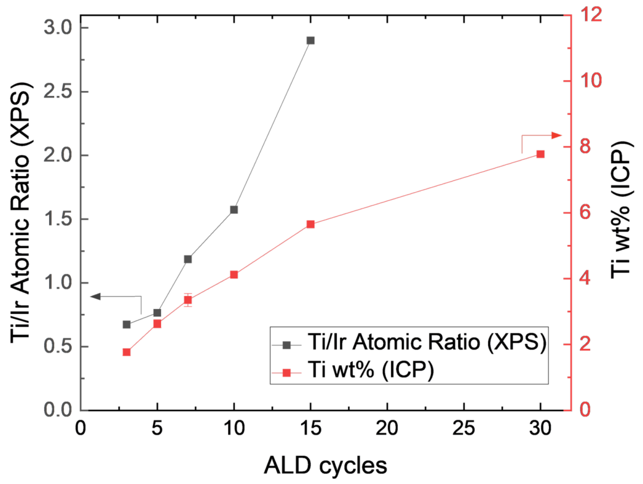

Supplementary Fig. 6 | Compositional analysis as a function of TiO_2-ALD cycle number. Atomic Ti/Ir ratios and Ti weight percentages of TiO_2-coated IrO_2 as a function of ALD cycle number. The Ti/Ir atomic ratios obtained by XPS (left axis) and the bulk Ti weight fractions measured by ICP-OES (right axis) both increase monotonically with ALD cycle number, demonstrating well-controlled and quasi-linear growth of the TiO_2 coating layer across surface-sensitive and bulk-averaged measurements.

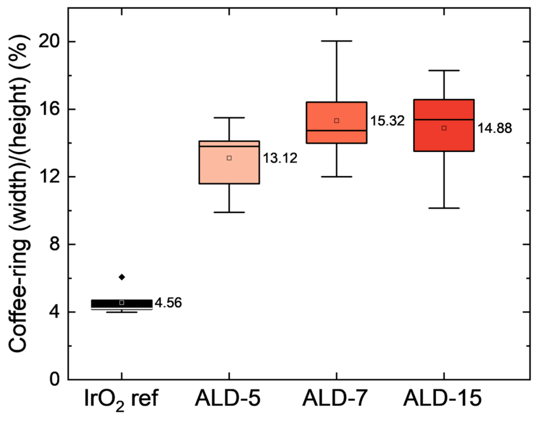

Supplementary Fig. 7 | Coffee-ring analysis of surface properties of TiO_2-coated IrO_2. Box-and-whisker plots of the width-to-height ratio of dried droplets for pristine IrO_2 and TiO_2-coated IrO_2 (5, 7, and 15 ALD cycles). The systematic increase followed by saturation of the ratio with ALD cycle number demonstrates progressively enhanced surface hydrophilicity with increasing TiO_2 coating coverage.

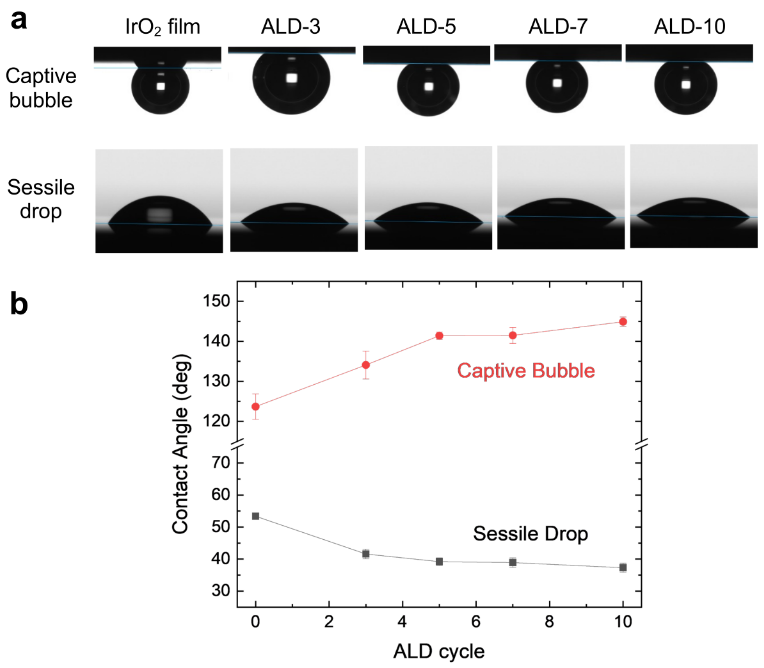

Supplementary Fig. 8 | Drop shape analysis (DSA) of TiO_2-coated IrO_2 model films. a, Representative optical images of air captive bubbles and water sessile drops on pristine IrO_2 model films and TiO_2-coated IrO_2 films (3, 5, 7, and 10 ALD cycles). **b,** Quantitative evolution of contact angles for sessile drops (black squares) and air captive bubbles (red circles) as a function of ALD cycle number. The progressive increase in captive bubble contact angles, accompanied by a decrease in sessile drop angles, indicates a systematic transition toward a more hydrophilic and aerophobic surface state with increasing TiO_2 coating coverage.

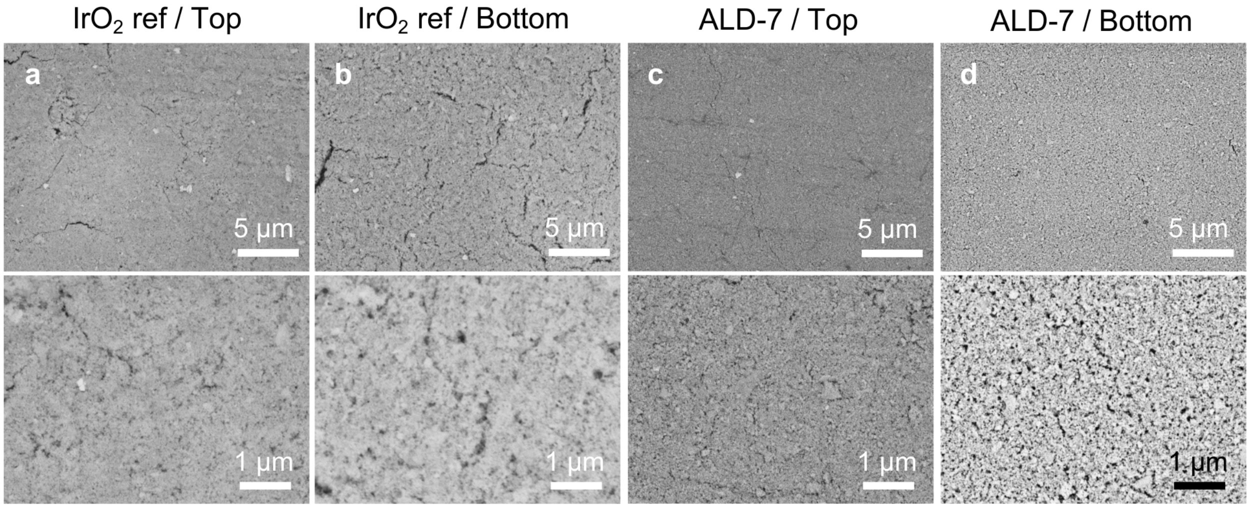

Supplementary Fig. 9 | Surface scanning electron microscopy (SEM) analysis of TiO_2-coated IrO_2 anodes (ALD-7) anodes. SEM images of anode catalyst layers fabricated using the IrO_2 reference and the ALD-7 catalyst. **a, b,** SEM images of the IrO_2 reference anode surface facing the porous transport layer (PTL) (**a**) and membrane (**b**). **c, d,** Corresponding SEM images of the ALD-7 anode surface facing the PTL (**c**) and membrane (**d**). Compared with the IrO_2 reference, the ALD-7 anode exhibits a more uniform pore morphology across both PTL-facing and membrane-facing surfaces, indicating improved structural consistency of the catalyst layer.

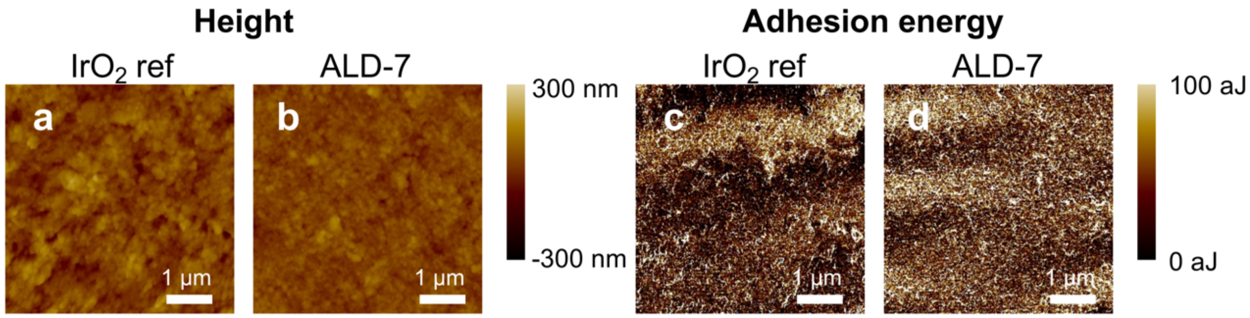

Supplementary Fig. 10 | AFM measurements of ALD-7 anodes. a, b, AFM height topography images of the IrO_2 reference anode (**a**)and the ALD-7 anode (**b**). The color scale indicates height variations from –300 to 300 nm. **c, d,** Corresponding AFM adhesion energy maps of the IrO_2 reference (**c**) and the ALD-7 anode (**d**), with adhesion energies ranging from 0 to 100 aJ. Compared with the IrO_2 reference, the ALD-7 anodes exhibit a more spatially uniform adhesion energy distribution, implying homogeneous ionomer distribution across the catalyst surface induced by TiO_2 coating. Quantitative AFM metrics are summarized in Supplementary Table 3.

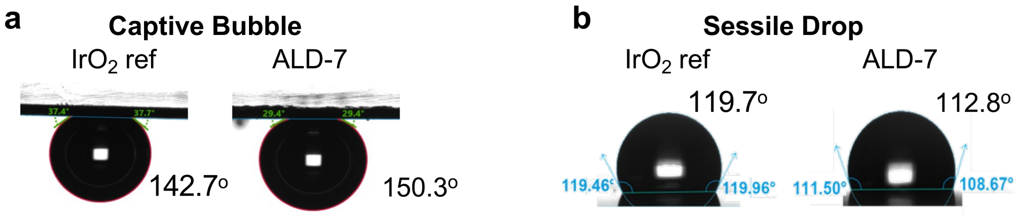

Supplementary Fig. 11 | Drop shape analysis of ALD-7 anodes. a, b, Contact angle measurements using air captive bubbles **(a)** and water sessile drops **(b)** on the IrO_2 reference anode and the ALD-7 anode. The annotated angles show that the ALD-7 anode exhibits a higher captive-bubble contact angle and a lower sessile-drop contact angle than the reference, indicating simultaneously enhanced aerophobicity and hydrophilicity induced by the TiO_2 coating.

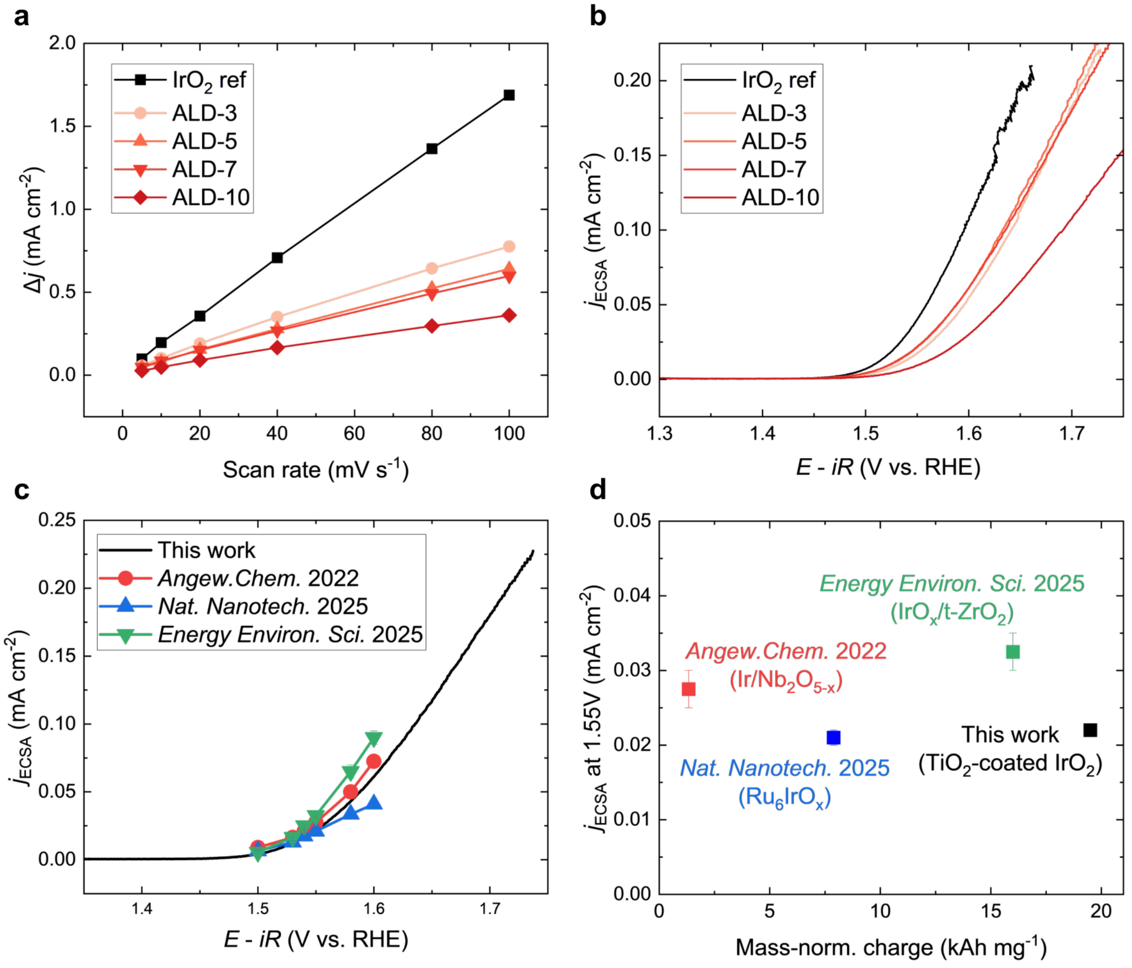

Supplementary Fig. 12 | RDE-derived intrinsic activity with benchmarking against state-of-the-art catalysts. **a,** Determination of electrochemical surface area (ECSA) from double-layer capacitance (C_{dl}) obtained from the linear relationship between scan rate and capacitive current density difference (Δj), where the slope is highest for the pristine IrO_2 and decreases progressively with increasing TiO_2-ALD cycle number. **b,** ECSA-normalized activity of the bare IrO_2 and TiO_2-coated catalysts, measured in 0.5 M H_2SO_4 (pH 0.29), recorded at 5 mV s^{-1} with 0.2 mg cm^{-2} Ir loading, 1,600 rpm, and 25 ºC. The ALD-3, ALD-5, and ALD-7 exhibit only a moderate decrease relative to bare IrO_2, whereas ALD-10 shows a substantial decline. The ohmic resistances for pristine IrO_2, ALD-3, ALD-5, ALD-7, and ALD-10 are 7.77, 8.15, 6.71, 8.89, and 7.21 Ω, respectively. **c,** Comparison of the ECSA-normalized activity of ALD-7 from this work with reported state-of-the-art Ir-based catalysts, demonstrating that the activity of ALD-7 falls within the range of literature values.[8,15,27] **d,** Correlation between ECSA-normalized activity and mass-normalized charge density, showing that this work achieves higher mass-normalized charge than state-of-the-art reports, indicating enhanced catalyst utilization and superior durability under high-current operation.

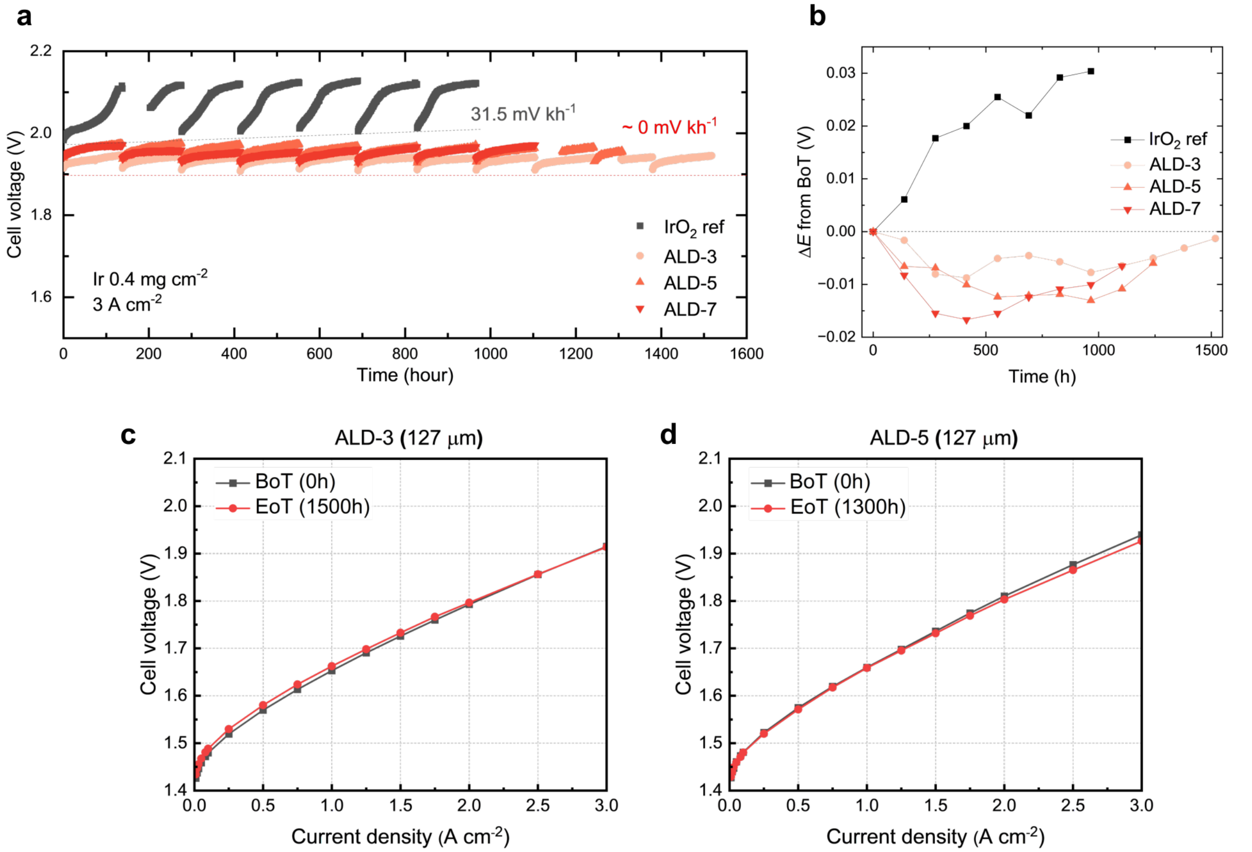

Supplementary Fig. 13 | Long-term stability and polarization analysis of TiO_2-coated IrO_2 anodes. a, Galvanostatic stability tests of PEMWE cells employing a 127 μm membrane, conducted at a constant current density of 3.0 A cm^{-2} with an Ir loading of 0.4 mg cm^{-2}. The IrO_2 reference anode (black squares) exhibits a degradation rate of 31.5 mV kh^{-1}, whereas the TiO_2-modified anodes (ALD-3, ALD-5, and ALD-7) show markedly enhanced stability with near-zero voltage drift over 1,500 h of operation. **b,** Comparison of the voltage change (ΔV), referenced to the beginning-of-test (BoT), during galvanostatic operation at 3.0 A cm^{-2} for the IrO_2 reference and TiO_2-coated IrO_2 anodes. The IrO_2 reference exhibits a monotonic voltage increase, whereas the TiO_2-modified anodes show an initial decrease over ~400–600 h, followed by a gradual rise, reflecting initial wetting and stabilization of the electrode structure. **c, d,** Polarization curves at the BoT and end-of-test (EoT) at 80 ºC with an Ir loading of 0.4 mg cm^{-2} for ALD-3 (**c**) and ALD-5 (**d**) anodes. The near-overlap of the BoT and EoT curves confirms negligible degradation.

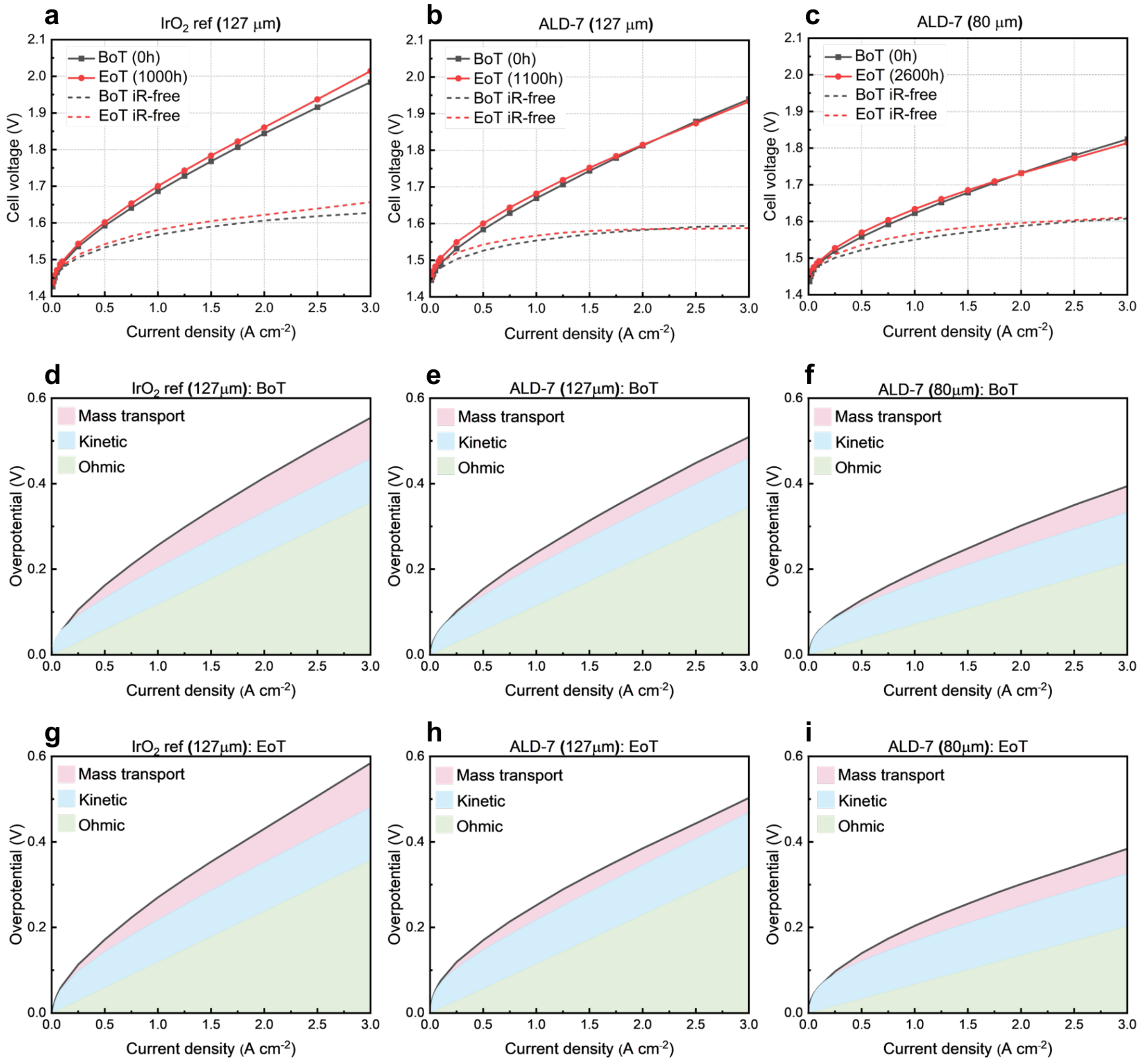

Supplementary Fig. 14 | Deconvolution of the total overpotential at the BoT and EoT. a–c, Polarization curves at BoT and EoT states at 80 °C with an Ir loading of 0.4 mg cm^{-2} for PEMWE cells assembled with: IrO_2 reference anode and 127 μm membrane (1,000 h EoT) (**a**); ALD-7 anode and 127 μm membrane (1,100 h EoT) (**b**); and ALD-7 anode and 80 μm membrane (2,600 h EoT) (**c**). Solid lines represent total cell voltage, while dashed lines indicate iR-free potentials. The IrO_2 reference displays a noticeable EoT voltage rise at 3 A cm^{-2}, whereas the ALD-7 anodes exhibit nearly overlapping BoT and EoT curves, demonstrating near-zero degradation. **d–f,** Deconvolution of overpotentials at BoT into mass-transport (η_{mt}, pink), kinetic (η_{kin}, blue), and ohmic (η_{ohm}, green) contributions for: IrO_2 reference (127 μm) (**d**); ALD-7 (127 μm) (**e**); and ALD-7 (80 μm) (**f**). **g–i,** Corresponding overpotential deconvolutions at EoT for: IrO_2 reference (127 μm) (**g**); ALD-7 (127 μm) (**h**); and ALD-7 (80 μm) (**i**). For the ALD-7 anode, a pronounced reduction in mass-transport overpotential is observed for both 80 μm and 127 μm membranes. In contrast, the IrO_2 reference exhibits a clear increase in mass-transport overpotential. Detailed performance metrics are summarized in Supplementary Table 7.

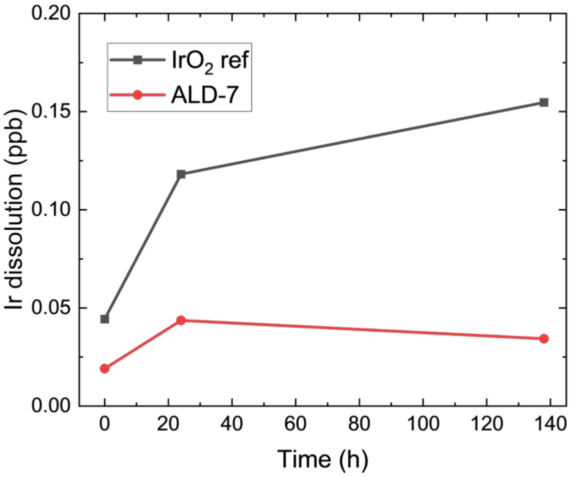

Supplementary Fig. 15 | Ir dissolution during PEMWE operation measured by inductively coupled plasma mass spectrometry (ICP-MS). Ir concentrations detected in the anode outlet water for the IrO_2 reference and ALD-7 anodes as a function of operation time. The reference IrO_2 anode shows a progressive increase in dissolved Ir concentration during operation, whereas the ALD-7 anode maintains substantially lower Ir concentrations close to the practical detection limit of ICP-MS, indicating suppressed Ir dissolution.

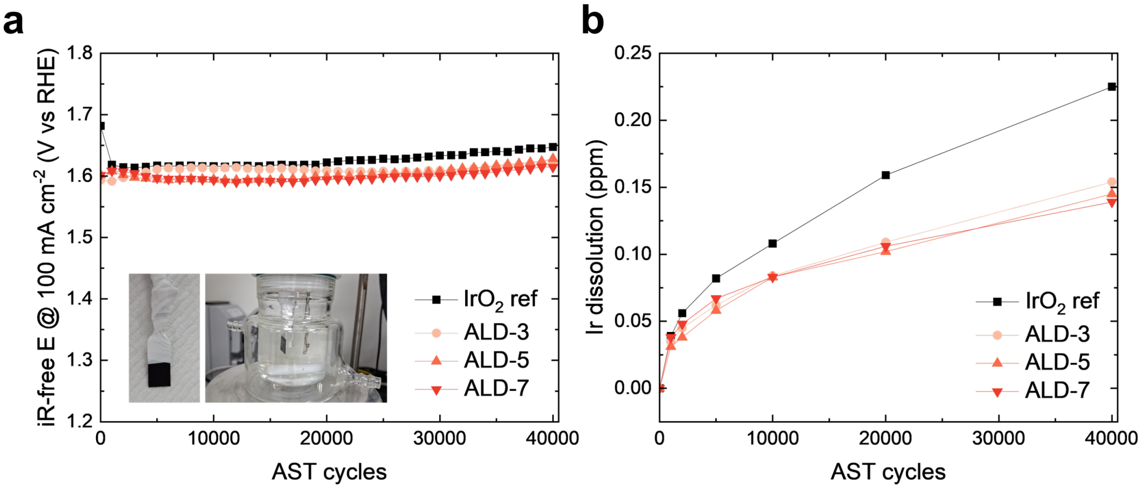

Supplementary Fig. 16 | Three-electrode accelerated stress test (AST) and Ir dissolution analysis of TiO_2-coated IrO_2. a, iR-free potential at 100 mA cm^{-2} recorded over 40,000 cycles of AST for the pristine IrO_2 reference and TiO_2-coated catalysts (ALD-3, ALD-5, and ALD-7). Measurements were performed in a three-electrode configuration using carbon paper electrodes in an acidic electrolyte. The inset shows photographic images of the carbon paper working electrode and the three-electrode cell setup used for the AST and chronopotentiometry (CP) measurements. **b,** Cumulative concentration of dissolved Ir in the electrolyte as a function of AST cycles, quantified by ICP–OES. The TiO_2-coated catalysts exhibit significantly suppressed Ir dissolution compared with the IrO_2 reference.

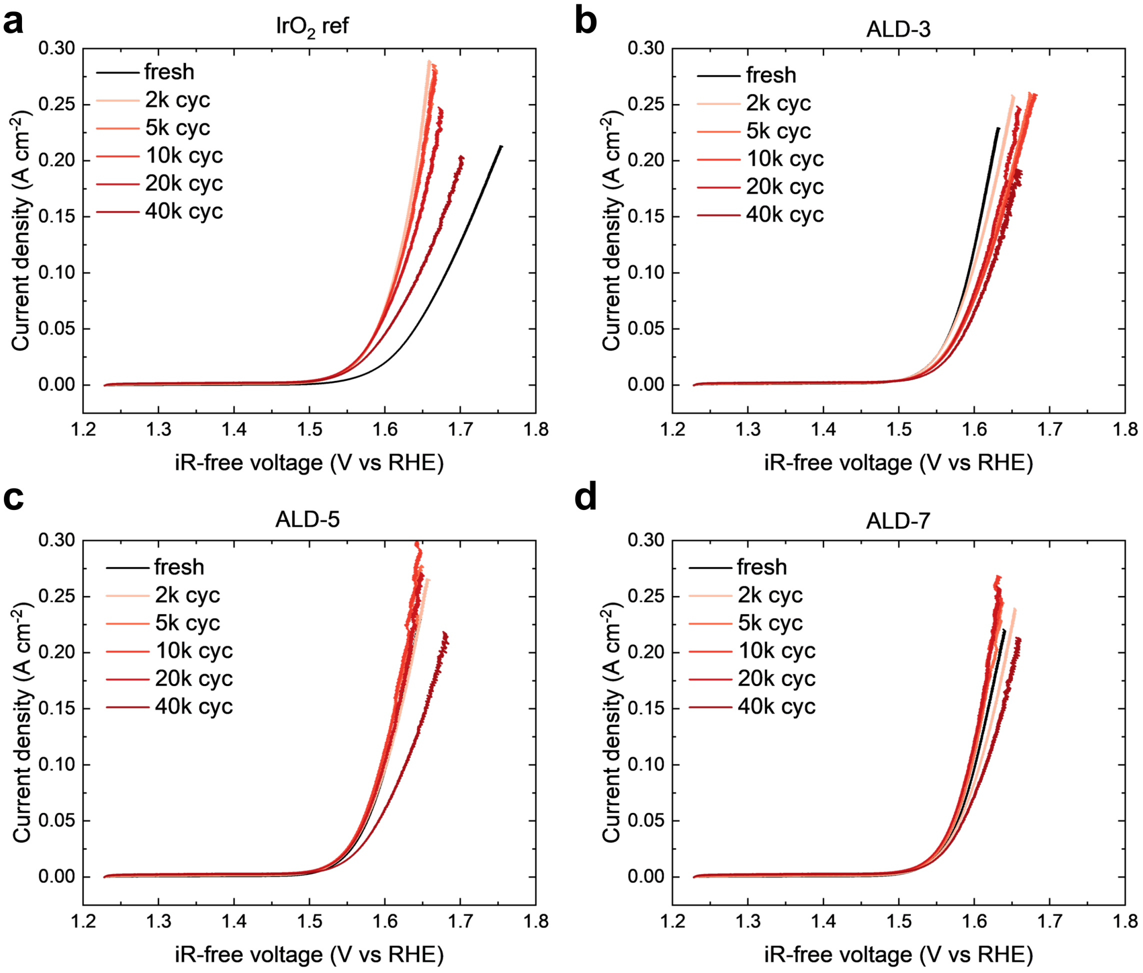

Supplementary Fig. 17 | Linear sweep voltammetry (LSV) curves at various cycle intervals during three-electrode AST. a–d, LSV curves recorded at various cycle intervals (fresh, 2k, 5k, 10k, 20k, and 40k cycles), measured in 0.5 M H_2SO_4 (pH 0.29), recorded at 50 mV s^{-1} with 0.4 mg cm^{-2} Ir loading, and 25 ºC, during AST for: IrO_2 reference (**a**); ALD-3 (**b**); ALD-5 (**c**); and ALD-7 (**d**) catalyst anodes. The initial ohmic resistances for IrO_2 reference, ALD-3, ALD-5, and ALD-7 are 2.21, 2.59, 2.51, and 2.66 Ω, respectively.

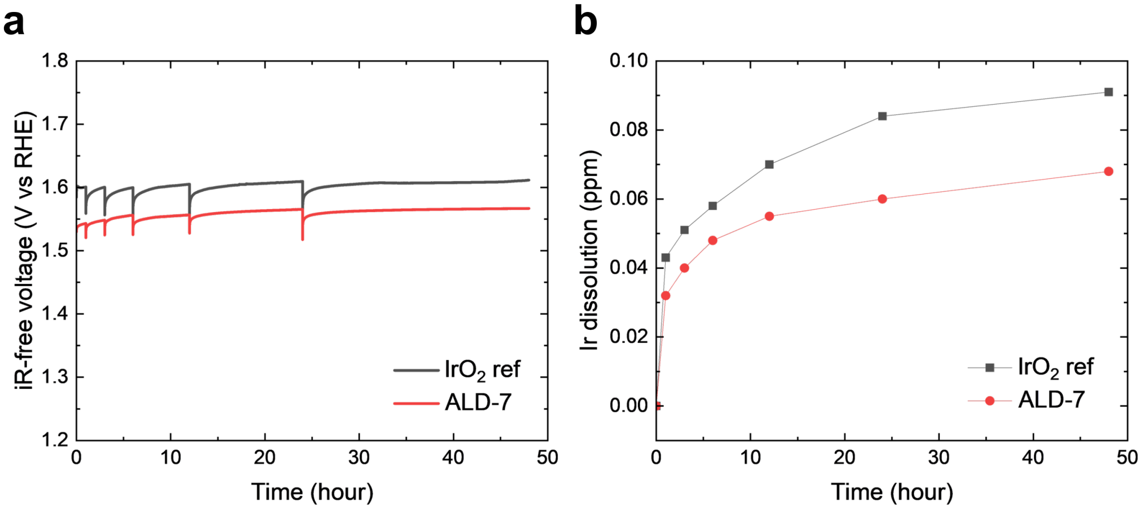

Supplementary Fig. 18 | Three-electrode CP evaluation and Ir dissolution analysis. a, CP stability profiles of the IrO_2 reference and the ALD-7 catalyst recorded at a constant current density of 10 mA cm^{-2} over 48 h. **b,** Cumulative Ir dissolution in the electrolyte as a function of CP time, quantified by ICP-OES. The significantly lower concentration measured for the ALD-7 catalyst relative to the IrO_2 reference confirms effective suppression of Ir dissolution during CP operation, consistent with trends observed under AST conditions.

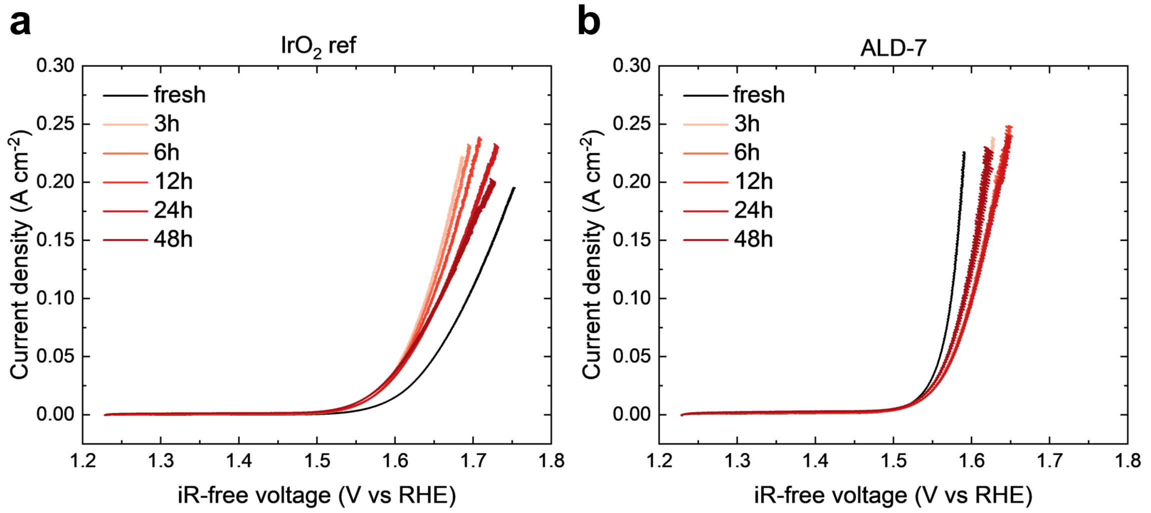

Supplementary Fig. 19 | LSV curves at various time intervals during three-electrode CP. a, b, Evolution of LSV curves recorded at various time intervals (0, 3, 6, 12, 24, and 48 h), measured in 0.5 M H_2SO_4 (pH 0.29), recorded at 50 mV s^{-1} with 0.4 mg cm^{-2} Ir loading, and 25 ºC, during CP for: the IrO_2 reference **(a)** and the ALD-7 catalyst anode **(b)**. The initial ohmic resistances for IrO_2 reference and ALD-7 are 2.42, and 2.81 Ω, respectively.

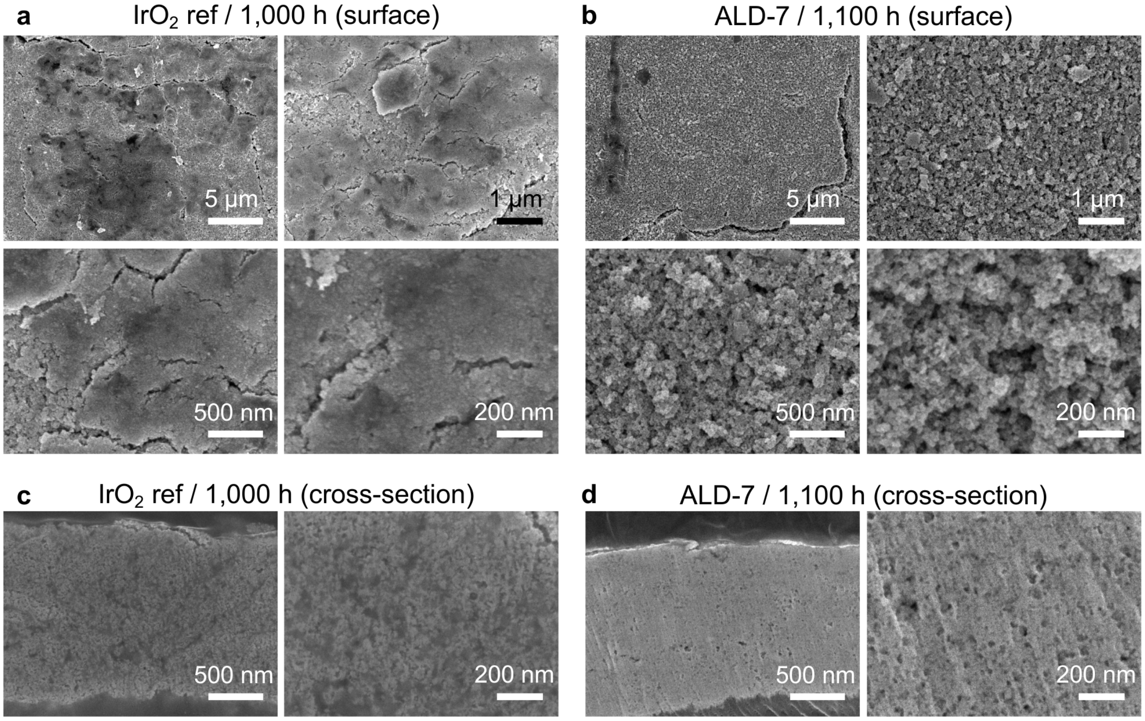

Supplementary Fig. 20 | Post-mortem surface and cross-sectional SEM analysis of ALD-7 anode. a, b, Surface SEM images of the IrO_2 reference anode after 1,000 h (**a**) and the ALD-7 catalyst anode after 1,100 h (**b**) of operation at 3.0 A cm^{-2}, both assembled with a 127 μm membrane. The pristine IrO_2 anode exhibits significant pore blockage, whereas the ALD-7 anode maintains a well-defined, homogenous porous morphology. **c, d,** Corresponding cross-sectional SEM images of the IrO_2 reference anode after 1,000 h (**c**) and the ALD-7 anode after 1,100 h (**d**) of operation. Consistent with the surface observations, the IrO_2 reference anode shows substantial loss of pore connectivity, whereas the ALD-7 anode preserves an open porous architecture across the catalyst layer.

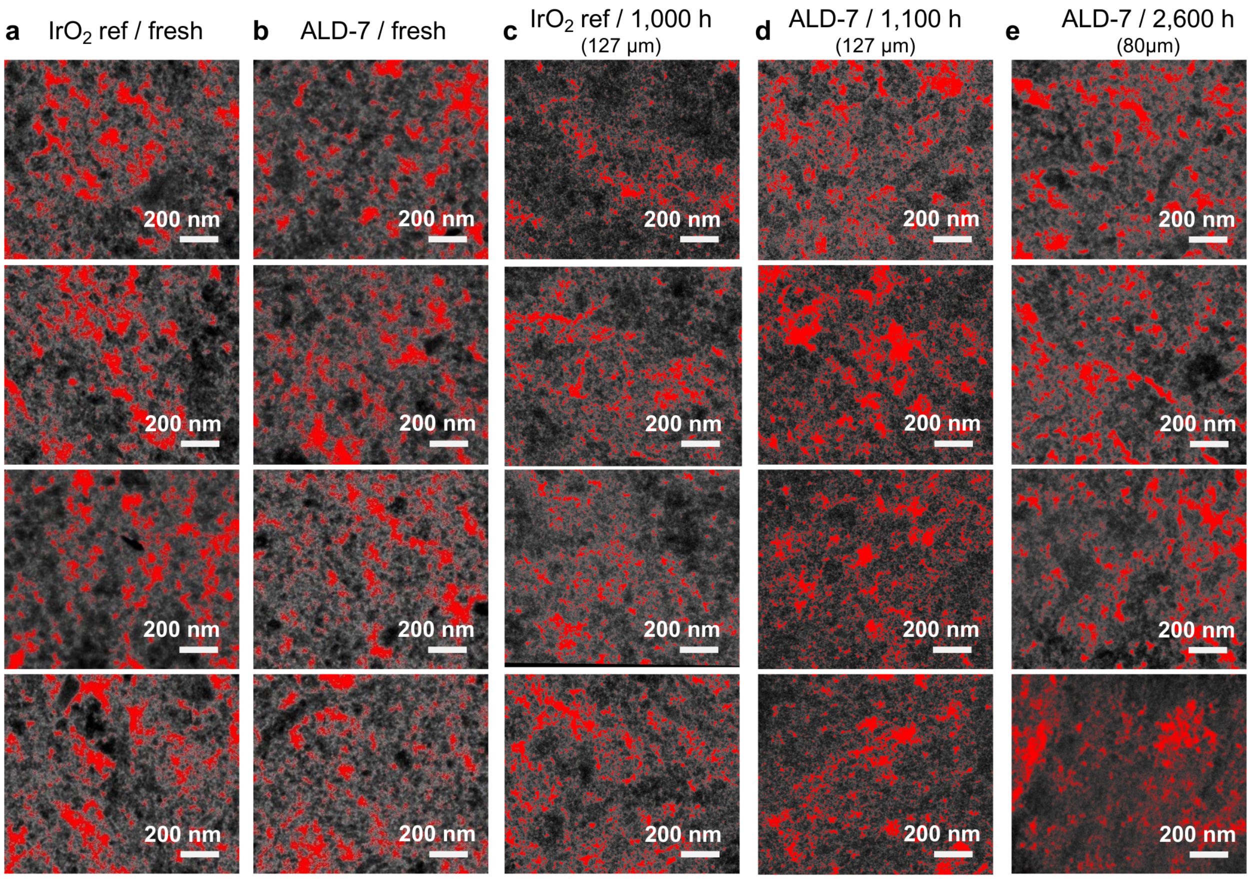

Supplementary Fig. 21 | Post-mortem cross-sectional TEM images used for quantitative porosity analysis. a, b, Representative cross-sectional TEM images of the fresh IrO_2 reference anode **(a)** and the ALD-7 anode **(b)** prior to electrochemical testing. The red overlays indicate pore regions within the catalyst layers identified for quantitative porosity analysis. **c,d,** Representative cross-sectional TEM images acquired after operation at 3.0 A cm^{-2} for the IrO_2 reference anode (1,000 h) **(c)** and the ALD-7 anode (1,100 h) **(d)**, both assembled with a 127 μm membrane. The IrO_2 reference anode exhibits a pronounced reduction in pore area after 1,000 h of operation, whereas the ALD-7 anode largely preserves its pore area after 1,100 h. **e,** Representative cross-sectional TEM image of the ALD-7 anode after extended operation at 3.0 A cm^{-2} for 2,600 h with an 80 μm membrane, showing sustained preservation of the porous structure. Scale bars represent 200 nm for all panels.

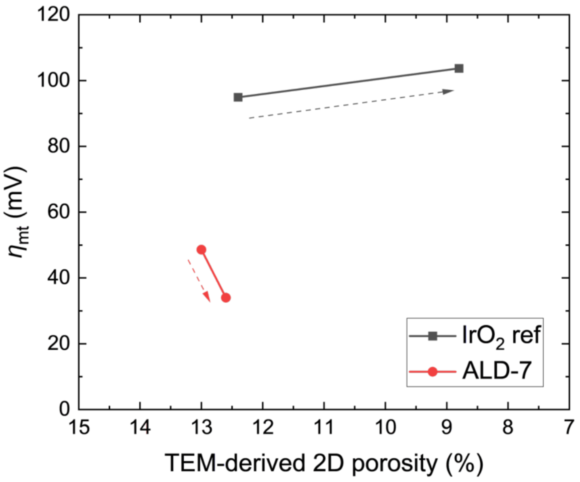

Supplementary Fig. 22 | Correlation between 2D porosity and mass-transport overpotential of anodes. Comparison of changes in η_{mt} at 3 A cm^{-2} during prolonged operation as a function of TEM-derived 2D porosity for IrO_2 reference and ALD-7 anodes. The IrO_2 reference exhibits a pronounced porosity loss (12.4% to 8.8%) after 1,000 h of operation, accompanied by an increase in η_{mt} (+8.8 mV). In contrast, the ALD-7 anode maintains its porosity (13.0% to 12.6%) after 1,100 h of operation and shows a decrease in η_{mt} (-14.6 mV). These results indicate that preserving catalyst-layer architecture is critical for mitigating mass-transport losses under high-current operation.

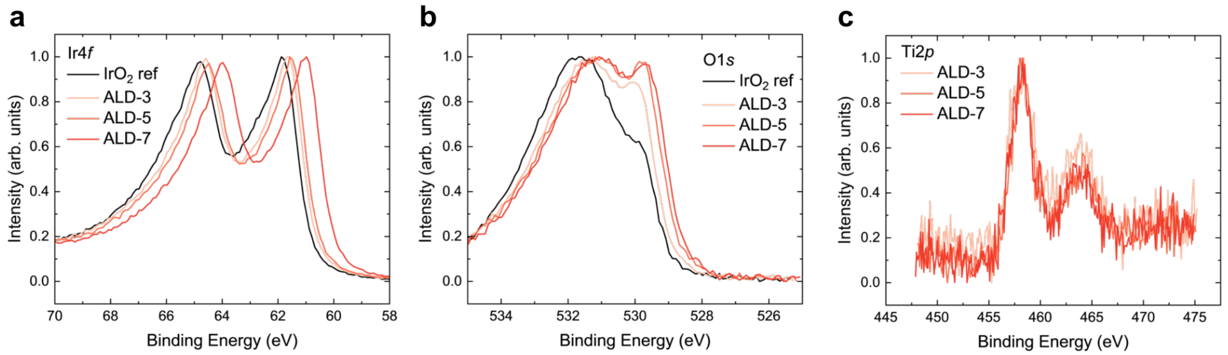

Supplementary Fig. 23 | XPS analysis of TiO_2-coated IrO_2 anodes. High-resolution XPS narrow scans for the IrO_2 reference and TiO_2-coated catalyst (3–7 cycles) anodes. **a,** Ir 4*f* core-level spectra. **b,** O 1*s* spectra. **c,** Ti 2*p* spectra. The anode-level XPS results exhibit trends that are consistent with those observed for the corresponding catalyst powders.

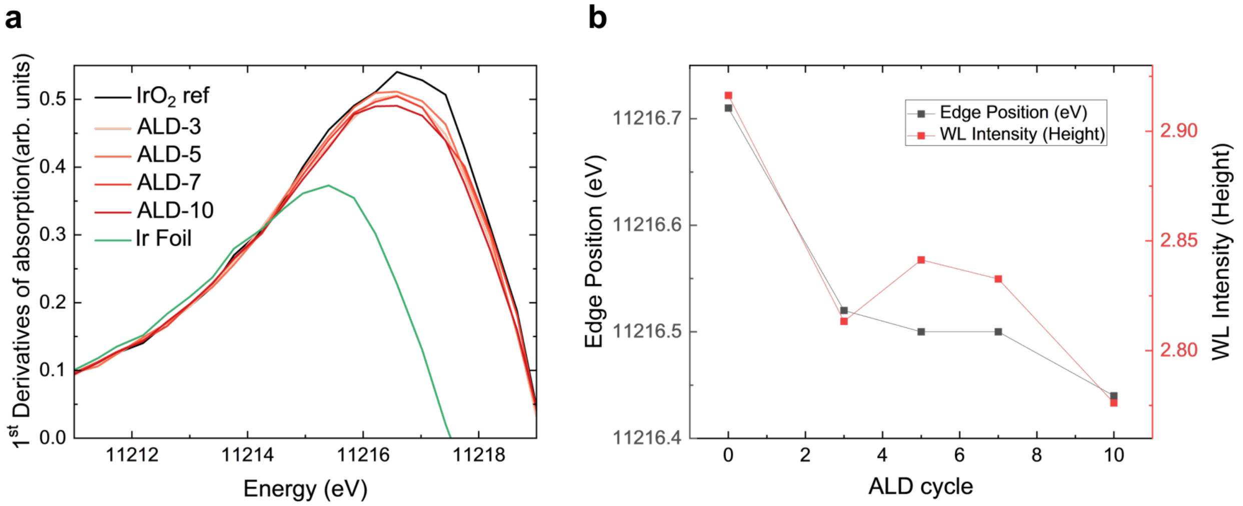

Supplementary Fig. 24 | XANES analysis of TiO_2-coated IrO_2 catalysts. a, First-order derivatives of the Ir L_3-edge XANES spectra for the IrO_2 reference, TiO_2-coated catalysts (3-10 cycles), and an Ir foil reference. **b,** Evolution of the absorption edge position (black squares) and white-line (WL) intensity (red squares) as a function of ALD cycle number. The systematic reduction in the absorption edge position and WL intensity indicates a reduction in the Ir valence state with increasing TiO_2 coverage.

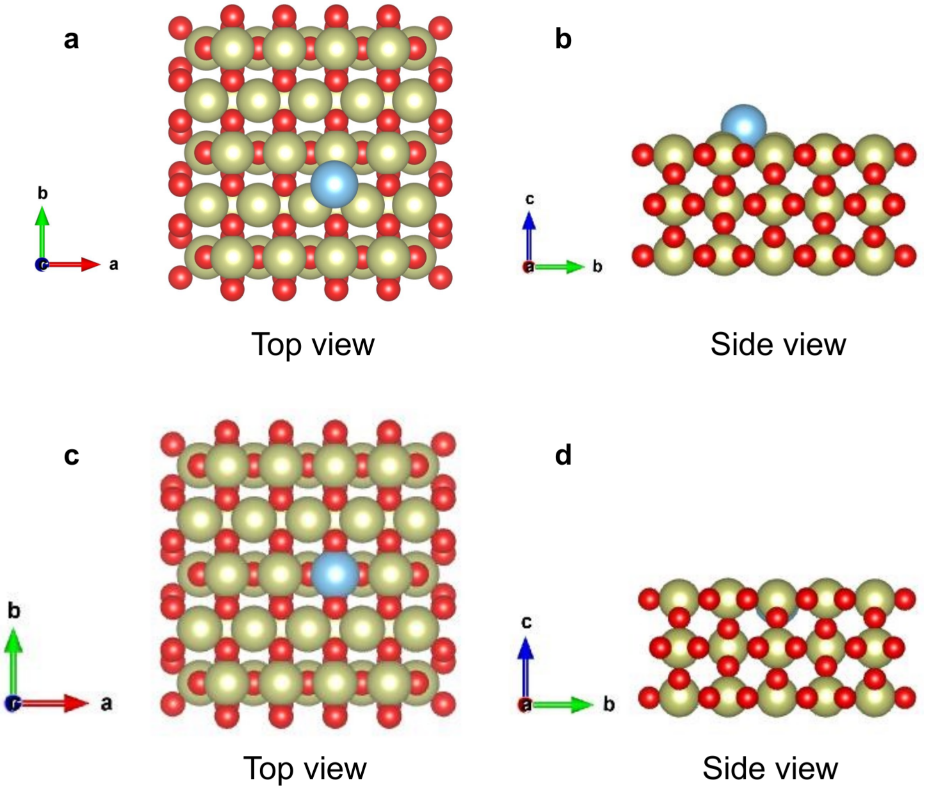

Supplementary Fig. 25 | Optimized atomic structures of the Ti-attached and Ti-doped IrO_2(110) surface models. a, b, Optimized atomic structures of the Ti-attached IrO_2(110) surface in top (**a**) and side (**b**) views. **c, d,** Optimized atomic structures of the Ti-doped IrO_2(110) surface in top (**c**) and side (**d**) views.

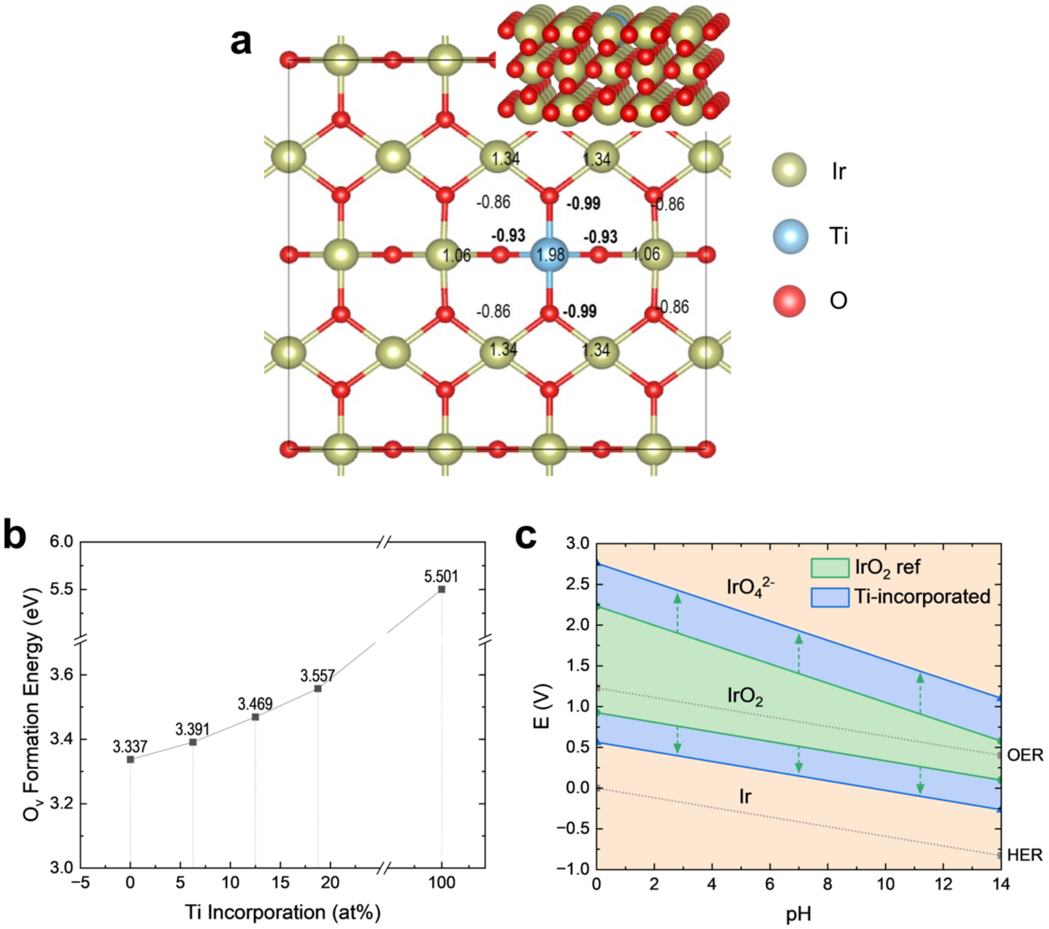

Supplementary Fig. 26 | Density functional theory (DFT) investigation of Ti-incorporated IrO_2. **a,** DFT-calculated atomic models of the Ti-doped IrO_2(110) surface shown in top and side views. The annotated values denote the localized atomic charges of Ir (gold), Ti (blue), and O (red), revealing Ti-induced electronic modification and a slight reduction of Ir atomic charges in the vicinity of the Ti site. **b,** Calculated oxygen vacancy formation energy (E_v) as a function of the incorporated Ti atom density (at%). The monotonic increase in E_v indicates that Ti incorporation suppresses oxygen vacancy formation, thereby stabilizing the IrO_2 lattice. **c,** Pourbaix diagram for the Ir–H_2O system comparing the stability regions of the pristine IrO_2 (green) and Ti-incorporated IrO_2 (25 at%, blue). The upward shift of the oxide stability window for Ti-incorporated IrO_2 relative to the OER and dissolution potentials indicates enhanced electrochemical stability against Ir oxidation and dissolution, suppressing the formation of soluble IrO_4^{2-} species.

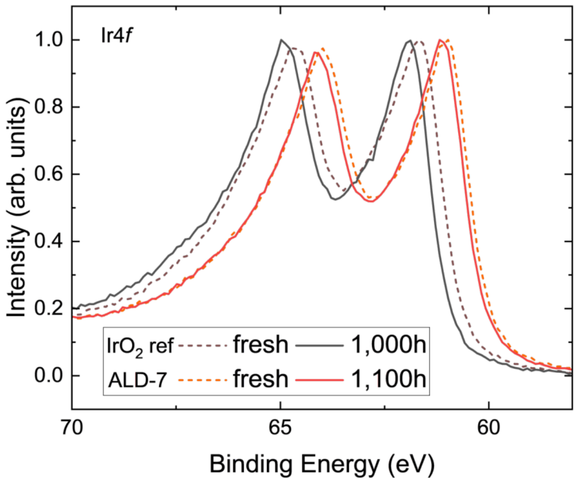

Supplementary Fig. 27 | XPS Ir 4*f* spectra of ALD-7 anodes after operation at 3.0 A cm^{-2}. The Ir 4*f* XPS spectra of both the IrO_2 reference (black) and ALD-7 (red) anodes show negligible changes between the fresh and post-operation states (1,000–1,100 h at 3.0 A cm^{-2}), while ALD-7 consistently maintains a lower binding energy than the IrO_2 reference, indicating that the initial electronic structure is well preserved under prolonged high-current operation.

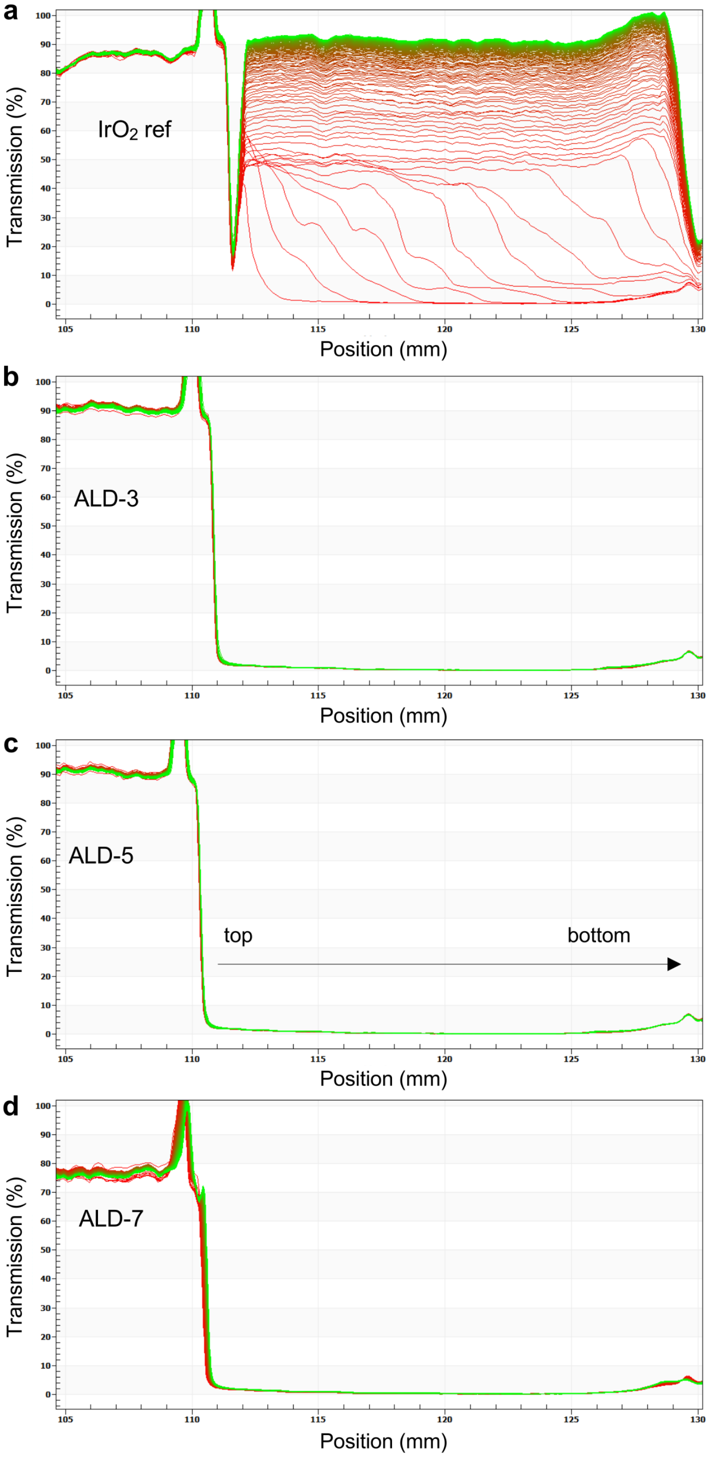

Supplementary Fig. 28 | Analytical centrifugation assessment of catalyst ink dispersion stability. Spatial and temporal transmission profiles of catalyst inks obtained by analytical centrifugation at 4,000 rpm for 2 h. The profiles show light transmission across the sample vial from top (left) to bottom (right) as a function of time for the IrO_2 reference **(a)**, ALD-3 **(b)**, ALD-5 **(c)**, and ALD-7 inks **(d)**. The pronounced increase and strong spatial evolution in transmission for the IrO_2 reference reflect rapid sedimentation and poor colloidal stability, whereas the negligible temporal and spatial changes observed for the TiO_2-modified inks (ALD-3 to ALD-7) indicate substantially enhanced dispersion stability under centrifugal stress.

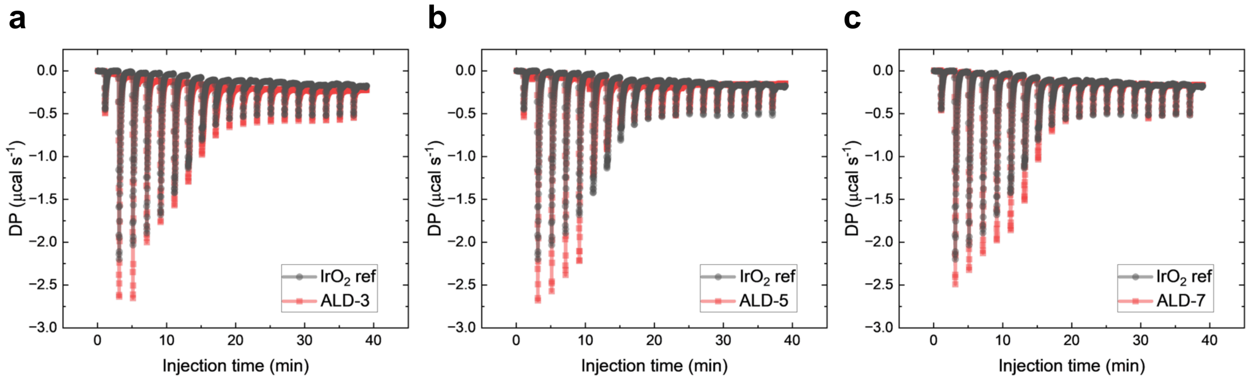

Supplementary Fig. 29 | Isothermal titration calorimetry (ITC) analysis of ionomer adsorption on TiO_2-coated IrO_2. Differential power (DP) thermograms obtained during ITC of an ionomer solution (2.5 mg mL^{-1}, 2 µL per injection) into aqueous catalyst dispersions (1 mg mL^{-1}) for ALD-3 (**a**), ALD-5, (**b**) and ALD-7 (**c**), in comparison with the IrO_2 reference. Gray traces correspond to the IrO_2 reference, while red traces represent the TiO_2-coated catalysts. The progressive attenuation and delayed saturation of the exothermic heat-flow peaks for the TiO_2-coated catalysts relative to the IrO_2 reference quantitatively reflect stronger and more sustained ionomer adsorption, demonstrating enhanced ionomer–catalyst interactions induced by TiO_2 coating.

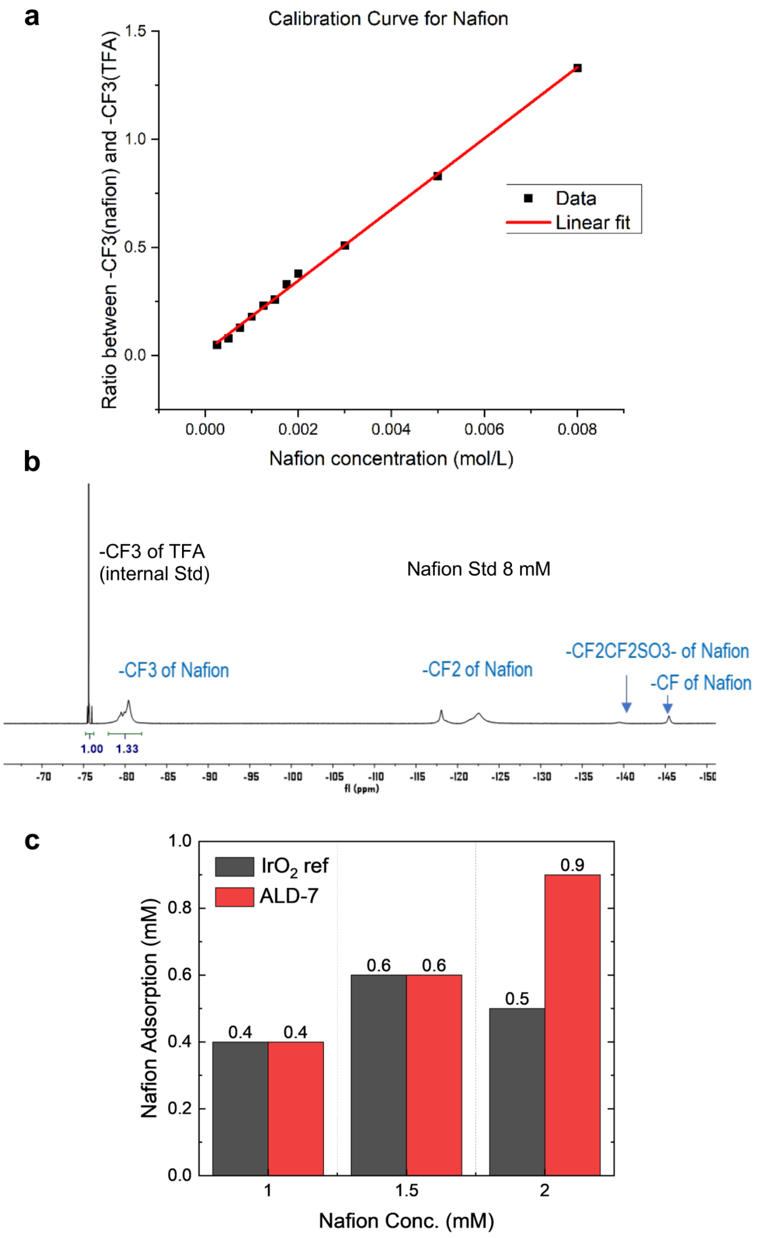

Supplementary Fig. 30 | Quantification of ionomer adsorption by ^{19}F nuclear magnetic resonance (NMR) spectroscopy. a, Calibration curve for ionomer quantification constructed by plotting the ratio of the $-CF_3$ peak areas (ionomer to internal standard, trifluoroacetic acid) as a function of ionomer concentration (0.25–8 mM). **b,** Representative ^{19}F NMR spectrum of an 8 mM ionomer standard solution with peak assignments for the perfluorinated backbone and side-chain groups. **c,** Quantitative comparison of ionomer adsorption at initial concentrations of 1.0, 1.5, and 2.0 mM for the IrO_2 reference and the ALD-7 catalyst. The higher adsorbed ionomer amount observed for the ALD-7 catalyst at 2.0 mM indicates enhanced ionomer adsorption on the TiO_2-coated catalyst surface.

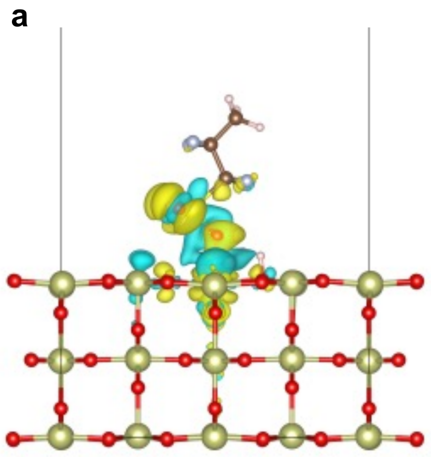

Supplementary Fig. 31 | DFT-calculated interfacial charge distribution upon ionomer adsorption. a,b, Side-view charge-density distribution at the adsorption interface of the ionomer side chain ($-SO_3H$) on pristine IrO_2(110) **(a)** and TiO_2-monolayer–coated IrO_2(110) **(b)**. Yellow regions denote charge accumulation, whereas blue regions indicate charge depletion. Compared with pristine IrO_2, the TiO_2-coated IrO_2 surface exhibits a more spatially extended charge-accumulation region at the adsorption interface, indicating enhanced interfacial polarization and stronger electrostatic interactions between the sulfonate group and the Ti-modified surface.

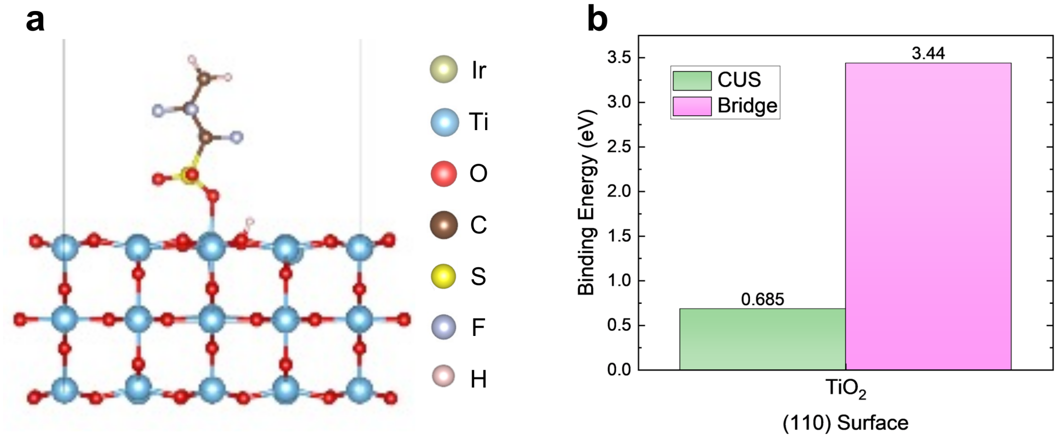

Supplementary Fig. 32 | DFT investigation of ionomer adsorption on pristine TiO_2. a, DFT-calculated optimized side-view structure of the ionomer side chain ($-SO_3H$) adsorbed on the pristine TiO_2(110) surface. Atomic spheres represent Ti (blue), O (red), C (brown), S (yellow), F (purple), and H (pink). **b,** Calculated ionomer binding energies at coordinatively unsaturated sites (CUS) and bridge sites on pristine TiO_2(110). The positive binding energies at both adsorption sites indicate energetically unfavorable ionomer adsorption on pristine TiO_2, in contrast to the stabilized adsorption observed in Ti-modified IrO_2 surfaces.

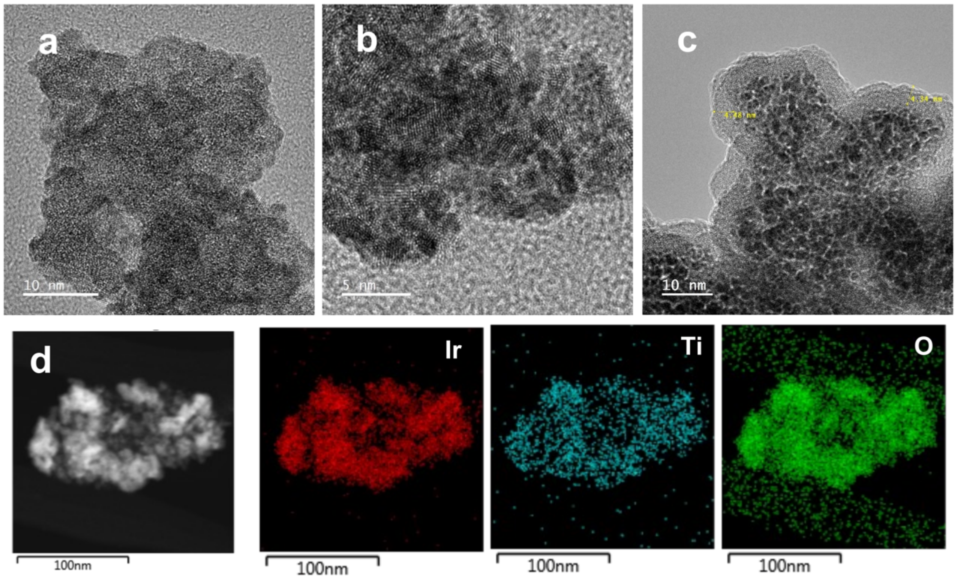

Supplementary Fig. 33 | TEM image and STEM-EDS elemental maps of TiO_2-coated amorphous-IrO_x (a-IrO_x@TiO_2). a–c, Representative high-resolution transmission electron microscopy (HR-TEM) images of amorphous IrO_x catalysts: pristine amorphous IrO_x reference catalyst (a-IrO_x) (**a**); TiO_2-ALD 3-cycle-coated catalyst (a-ALD-3) (**b**); and TiO_2-ALD 30-cycle-coated catalyst (a-ALD-30) (**c**). The images reveal the disordered nature of the base IrO_x and the subsequent formation of a conformal TiO_2 overlayer with increasing ALD cycles. **d,** STEM-EDS elemental mapping of the a-ALD-3 catalyst. The composite and individual maps for Ir (red), Ti (cyan), and O (green) demonstrate the uniform and continuous distribution of the TiO_2 coating across the amorphous catalyst surface (Supplementary Note 2).

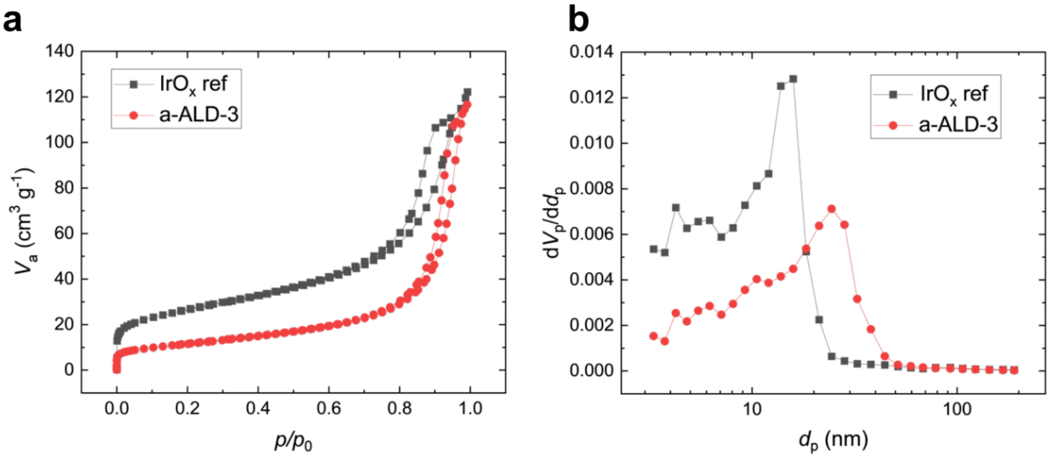

Supplementary Fig. 34 | BET surface area analysis of a-IrO_x@TiO_2. a, N_2 adsorption–desorption isotherms for the a-IrO_x reference (black squares) and the a-ALD-3 catalyst (red circles). **b,** Corresponding BJH pore-size distributions derived from the desorption branches for the a-IrO_x reference and a-ALD-3. The distribution highlights a reduced total pore volume and suppressed mesopore contribution relative to the a-IrO_x reference, consistent with partial pore filling by the conformal TiO_2 coating. Quantitative BET surface areas and pore volumes are summarized in Supplementary Table 10.

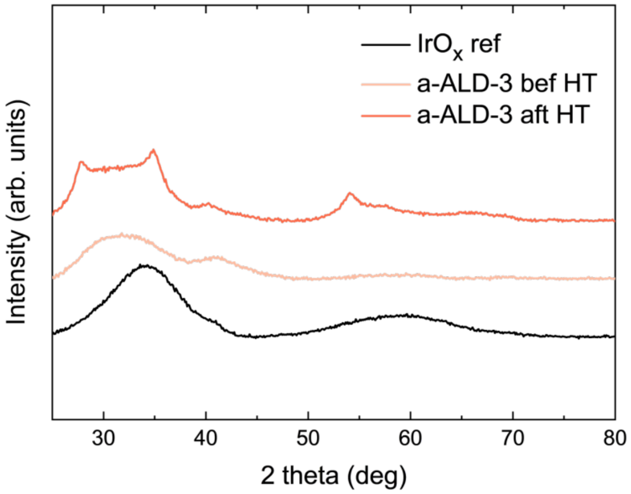

Supplementary Fig. 35 | XRD analysis of phase evolution in a-IrO_x@TiO_2. XRD patterns of the a-IrO_x reference (black) and the a-ALD-3 catalyst recorded before (light red) and after (dark red) heat treatment. Although weak diffraction features emerge after heat treatment, indicative of partial crystallization, the catalysts remain predominantly amorphous, with no sharp reflections corresponding to long-range ordered IrO_2 phase.

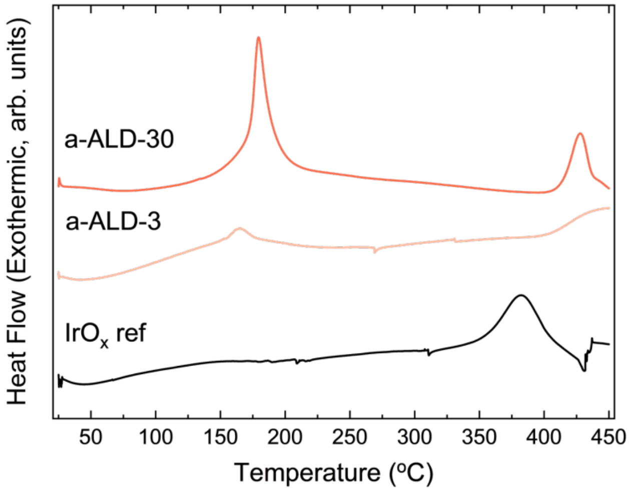

Supplementary Fig. 36 | DSC thermal analysis of a-IrO_x@TiO_2. DSC thermograms of the a-IrO_x reference (black), a-ALD-3 (light red), and a-ALD-30 (dark red). Distinct exothermic features observed at ∼150–200 ºC are assigned to the crystallization of the amorphous TiO_2 coating, with increasing intensity at higher ALD cycle numbers, whereas broader exothermic features appearing at ∼350–450 ºC correspond to the crystallization of IrO_2.

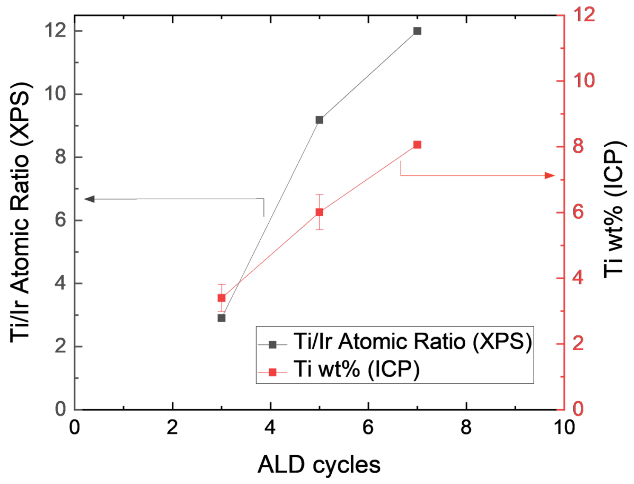

Supplementary Fig. 37 | Compositional analysis as a function of ALD cycle number for a-IrO_x@TiO_2. Atomic Ti/Ir ratios and Ti weight percentages of TiO_2-coated a-IrO_x as a function of ALD cycle number. The Ti/Ir atomic ratios obtained by XPS (left axis) and the bulk Ti weight fractions measured by ICP–OES (right axis) increase systematically with ALD cycle number, confirming controlled and approximately linear growth of the TiO_2 coating layer with increasing ALD cycles.

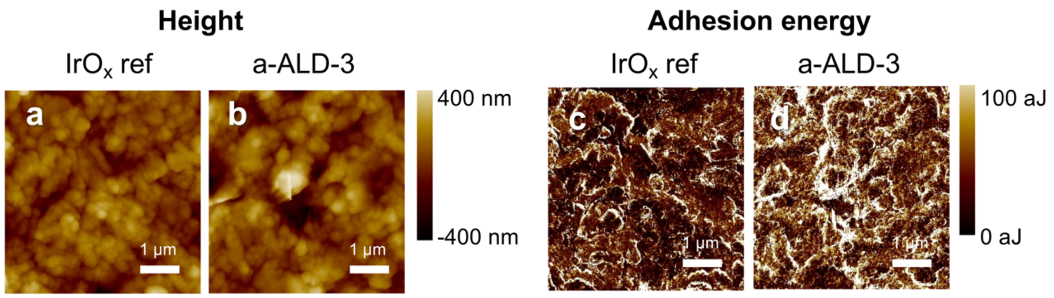

Supplementary Fig. 38 | AFM measurements of a-IrO_x@TiO_2 anode. a, b, AFM height topography images of the a-IrO_x reference (**a**) and a-ALD-3 anodes (**b**). The height scale ranges from -400 to 400 nm. **c, d,** Adhesion energy maps measured by AFM for the a-IrO_x reference (**c**) and a-ALD-3 anodes (**d**). The adhesion energy scale ranges from 0 to 100 aJ. The higher adhesion energy observed for the a-ALD-3 anode suggests increased ionomer coverage on the TiO_2-coated amorphous catalyst surface. Quantitative AFM data are summarized in Supplementary Table 11.

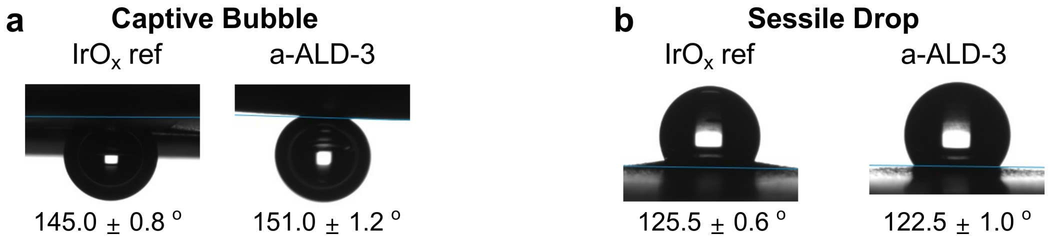

Supplementary Fig. 39 | Drop shape analysis of a-IrO$_x$@TiO$_2$ anode. a, Contact angle measurements using air captive bubbles on the a-IrO$_x$ reference and the a-ALD-3 anode. The a-ALD-3 anode shows a higher captive bubble contact angle ($151.0 \pm 1.2°$) compared to the reference ($145.0 \pm 0.8°$). **b,** Contact angle measurements using water sessile drops on the a-IrO$_x$ reference and the a-ALD-3 anode. The a-ALD-3 anode exhibits a lower sessile drop contact angle ($122.5 \pm 1.0°$) compared to the reference ($125.5 \pm 0.6°$). These trends confirm that the TiO_2 coating shifts the anode surface toward a more hydrophilic and aerophobic state.

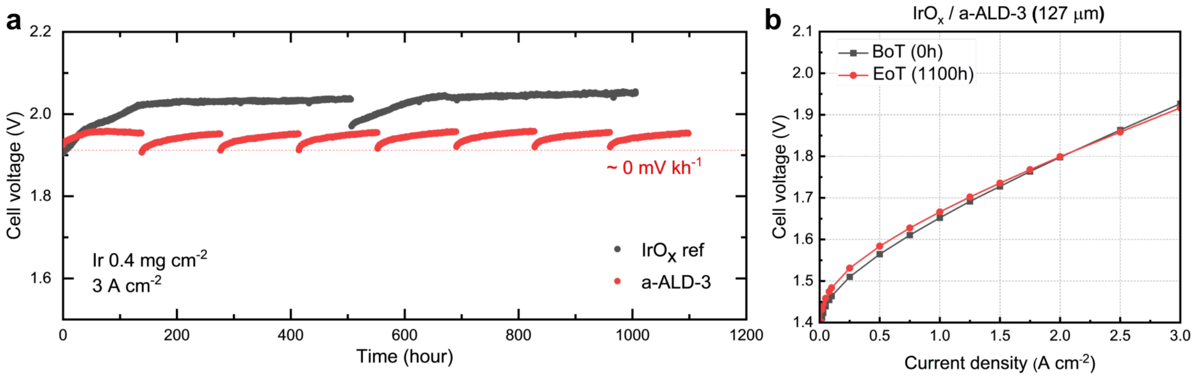

Supplementary Fig. 40 | Long-term stability and polarization analysis of a-IrO$_x$@TiO$_2$ anode. a, Galvanostatic stability tests of PEMWE cells at a constant current density of 3.0 A cm^{-2}, employing the a-IrO$_x$ reference and the a-ALD-3 anode, both assembled with a 127 μm membrane. The amorphous reference (black circles) exhibits a pronounced increase in cell voltage over time, whereas the a-ALD-3 anode (red circles) maintains exceptional stability with near-zero degradation over 1,100 h of operation, mirroring the durability improvement observed in the rutile IrO_2 system. **b,** Polarization curves at the BoT (0 h) and EoT (1,100 h) states at 80 °C with an Ir loading of 0.4 mg cm^{-2} for the PEMWE cell assembled with the a-ALD-3 anode. The negligible shift between the BoT and EoT curves confirms minimal performance degradation.

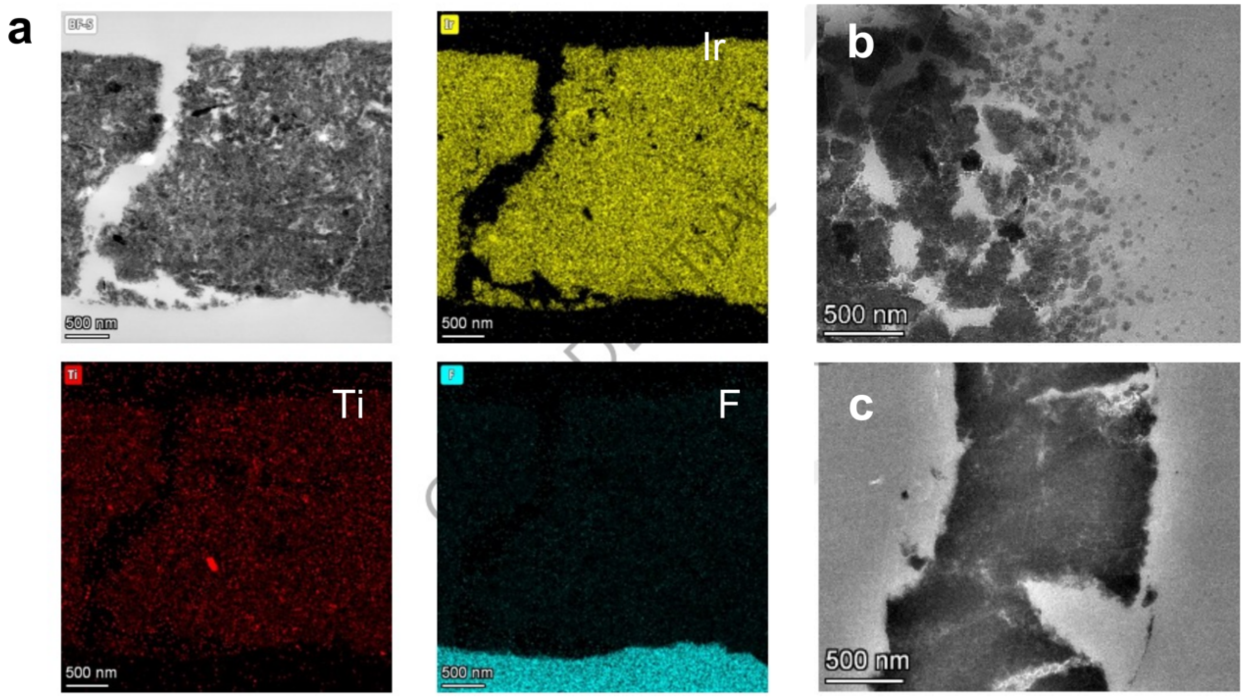

Supplementary Fig. 41 | Post-mortem analysis of a-IrO_x@TiO_2 anode. a, Cross-sectional TEM image and corresponding EDS mapping of a fresh a-ALD-3 anode. The elemental maps for Ir (yellow), Ti (red), and F (cyan) demonstrate a homogeneous distribution of the catalyst and ionomer within the electrode architecture. **b,** Cross-sectional TEM image of the a-IrO_x reference anode after 500 h of operation at 3.0 A cm^{-2}. The image reveals severe structural degradation accompanied by migrated Ir species within the membrane region, indicative of interfacial deterioration and catalyst dissolution. **c,** Cross-sectional TEM image of the a-ALD-3 anode after 1,100 h of operation at 3.0 A cm^{-2}. The a-ALD-3 electrode maintains structural integrity with no detectable Ir migration into the membrane region, consistent with the behavior observed for the rutile IrO_2 system. Scale bars for all panels represent 500 nm.

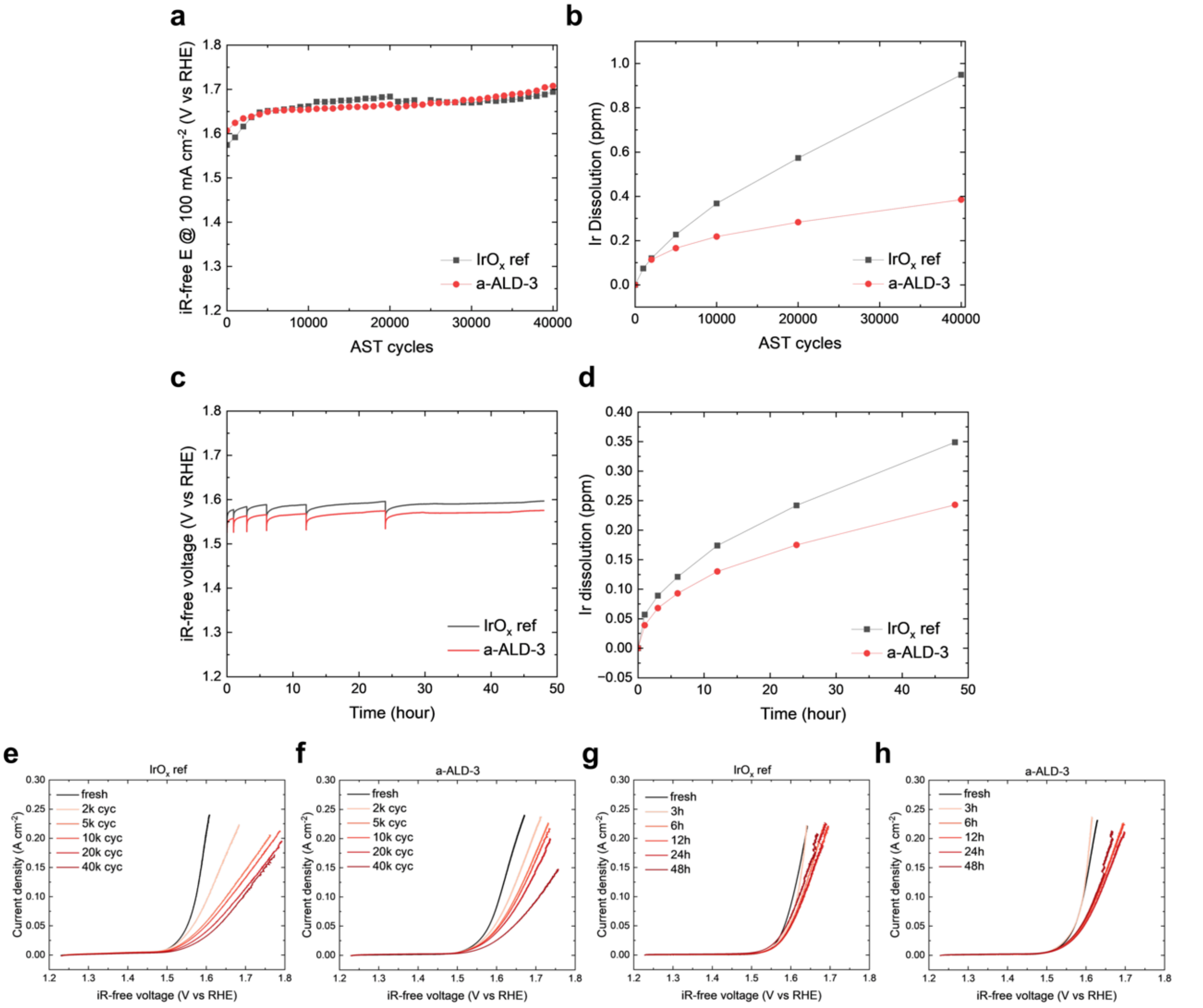

Supplementary Fig. 42 | Three-electrode durability and Ir dissolution analysis of a-IrO_x@TiO_2. **a,** iR-free potential measured at 100 mA cm^{-2} as a function of AST cycles (up to 40,000 cycles) for the a-IrO_x reference and the a-ALD-3 catalyst. **b,** Cumulative Ir concentration dissolved into the electrolyte during the AST, quantified by ICP-OES, showing substantially reduced Ir leaching for the a-ALD-3 catalyst relative to the amorphous reference. **c,** CP stability profiles recorded at a constant current density of 10 mA cm^{-2} in a three-electrode configuration for the a-IrO_x reference and the a-ALD-3 catalyst. **d,** Corresponding cumulative Ir dissolution measured during the CP test, confirming effective suppression of Ir dissolution by the TiO_2 coating. **e, f,** LSV curves recorded at selected AST cycle intervals (fresh to 40k cycles), measured in 0.5 M H_2SO_4 (pH 0.29), recorded at 50 mV s^{-1} with 0.4 mg cm^{-2} Ir loading, and 25 ºC, for the a-IrO_x reference **(e)** and the a-ALD-3 catalyst **(f)**. The initial ohmic resistances for a-IrO_x reference and a-ALD-3 are 2.57 and 2.31 Ω, respectively. **g, h,** LSV curves recorded at selected time intervals (fresh to 48 h) during the CP test, measured in 0.5 M H_2SO_4 (pH 0.29), recorded at 50 mV s^{-1} with 0.4 mg cm^{-2} Ir loading, and 25 ºC, for the a-IrO_x reference **(g)** and the a-ALD-3 catalyst **(h)**. The initial ohmic resistances for a-IrO_x reference and a-ALD-3 are 2.64 and 2.58 Ω, respectively.

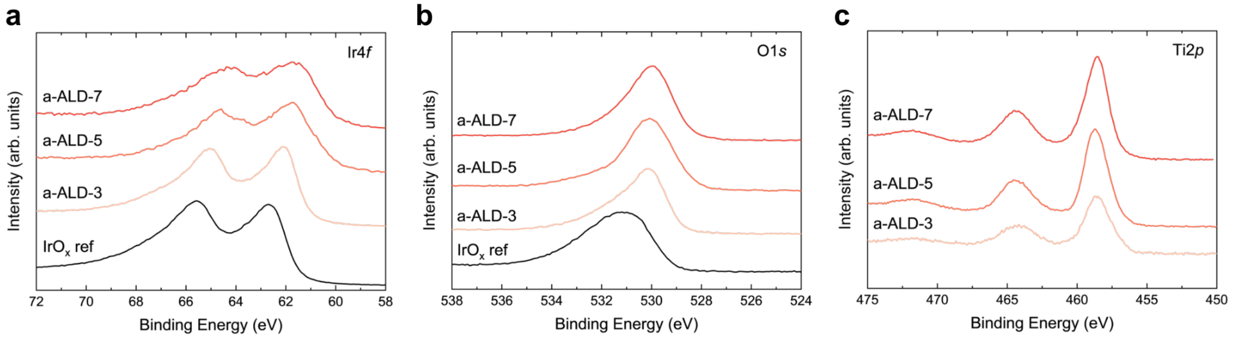

Supplementary Fig. 43 | XPS analysis of a-IrO$_x$@TiO$_2$. High-resolution XPS narrow scans of the a-IrO$_x$ reference and TiO$_2$-coated catalysts (a-ALD-3 to a-ALD-7). **a,** Ir 4*f* spectra showing a systematic shift to lower binding energy with increasing TiO$_2$ ALD cycle number, consistent with the trend observed for rutile IrO$_2$. **b, c,** High-resolution XPS narrow scans of the O 1*s* (**b**) and Ti 2*p* (**c**) regions for the a-IrO$_x$ reference and catalysts coated with 3–7 ALD cycles. With increasing ALD cycle number, the lattice-oxygen contribution in the O 1*s* region and the Ti 2*p* signal intensity increase monotonically, indicating progressively enhanced TiO$_2$ surface coverage on the amorphous IrO$_x$ catalyst.

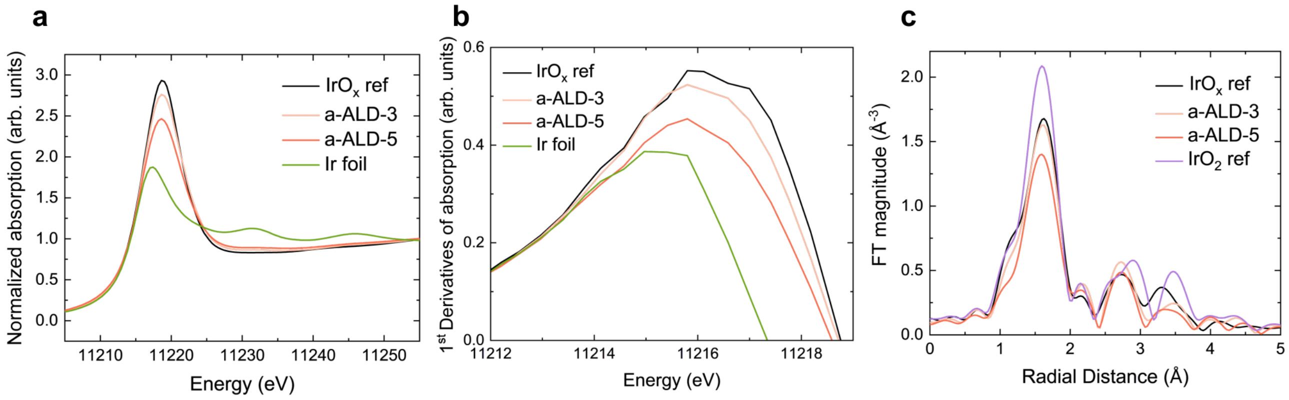

Supplementary Fig. 44 | XANES analysis of a-IrO$_x$@TiO$_2$ a, Ir L$_3$-edge XANES spectra for the a-IrO$_x$ reference and TiO$_2$–coated catalysts (a-ALD-3 and a-ALD-5), with an Ir foil reference. A progressive decrease in white-line intensity is observed with increasing ALD cycle number, consistent with the trend observed for rutile IrO$_2$. **b,** First derivatives of the XANES spectra showing a leftward shift of the absorption edge with increasing TiO$_2$-ALD cycle number, indicative of a reduced Ir oxidation state. **c,** Fourier-transformed EXAFS spectra at the Ir edge showing a gradual decrease in Ir−O coordination with increasing TiO$_2$ ALD cycle number, indicating TiO$_2$-induced reduction in the Ir valence state.

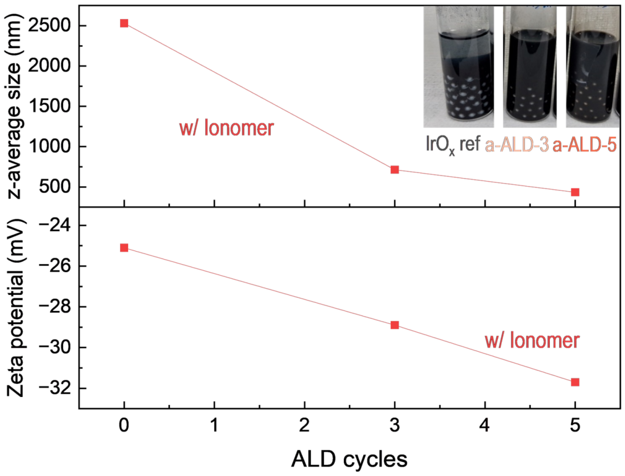

Supplementary Fig. 45 | Colloidal stability and surface charge of a-IrO_x@TiO_2. *z*-average hydrodynamic size and zeta potential of a-IrO_x@TiO_2 catalysts as a function of ALD cycle number, measured in the presence of ionomer. The TiO_2 coating progressively decreases aggregate size relative to the a-IrO_x reference and yields increasingly negative zeta potentials, indicating enhanced ionomer adsorption. The inset provides photographic evidence of improved colloidal stability for a-ALD-3 and a-ALD-5 compared to the IrO_x reference. This trend is consistent with that observed for rutile IrO_2 systems.

Supplementary Table 1 | BET surface area and pore analysis of IrO_2 reference and ALD-7 catalysts.

Catalyst	IrO_2 ref	ALD-7
Surface area ($a_{S,\mathrm{BET}}$) (m^2 g^{-1})	60.853	46.617
Pore volume ($p/p_0 = 0.990$) (cm^3 g^{-1})	0.1299	0.167
Mean pore diameter (nm)	8.5374	14.329

Supplementary Table 2 | Mercury intrusion porosimetry for IrO_2 reference and ALD-7 anodes.

Property	IrO_2 ref	ALD-7
Porosity (%)	47.2	50.8
Pore area (m^2 g^{-1})	0.914	0.971
Avg. Pore Size (nm)	12.45	12.66

Supplementary Table 3 | AFM measurements for IrO_2 reference and TiO_2-coated IrO_2 anodes.

AFM Measurement	IrO_2 ref	ALD-3	ALD-5	ALD-7
Roughness [Sa] (nm)	57.7 ± 5.4	71.2 ± 3.9	40.4 ± 3.3	59.0 ± 7.3
Stiffness (N m^{-1})	443.2 ± 111.7	428.5 ± 37.3	264.7 ± 56.2	159.5 ± 44.0
Adhesion Energy (aJ)	39.4 ± 2.9	35.1 ± 4.8	90.8 ± 9.4	108.3 ± 8.7

Supplementary Table 4 | ECSA-normalized activity of TiO_2-coated IrO_2 catalysts.

Catalyst	C_{dl} $mF\,cm^{-2}$	ECSA cm^2	j_{ECSA} at 1.55 V $mA\,cm^{-2}$
**IrO_2 ref**	16.73	418.3	0.040
**ALD-3**	7.54	188.5	0.018
**ALD-5**	6.26	156.4	0.022
**ALD-7**	5.78	144.4	0.022
**ALD-10**	3.52	88.1	0.009

Supplementary Table 5 | Benchmarking ECSA-normalized activity of Ir-based catalysts.

Catalyst	Rotating disk electrode (RDE) measurements			PEMWE measurements			
	**Loading** $mg\,cm^{-2}$	**Overpotential** mV at 10 $mA\,cm^{-2}$	j_{ECSA} $mA\,cm^{-2}$	**Ir loading** $mg\,cm^{-2}$	**Cell voltage** V	**Deg. Rate** $mV\,kh^{-1}$	**Mass-norm. charge,** $kAh\,mg^{-1}$
**TiO_2-coated IrO_2** (This work)	0.20	378	0.022 (1.55 V)	0.40	1.94 (3 $A\,cm^{-2}$, 80 °C)	0	19.5
**Ir/Nb_2O_{5-x}**[8]	1.6	218	0.0275 (1.55 V)	3.0	1.84 (3 $A\,cm^{-2}$, 80 °C)	0	1.33
**Ru_6IrO_x**[15]	0.40	240	0.021 (1.55 V)	0.38	1.94 (2 $A\,cm^{-2}$, 60 °C)	2.3	7.89
**IrO_x/t-ZrO_2**[27]	1.0	288	0.0325 (1.55 V)	0.10	1.89 (3 $A\,cm^{-2}$, 80 °C)	6.25	16.0
**IrO_x**[10]	0.28	263	0.035 (1.54 V)	0.28	1.90 (3 $A\,cm^{-2}$, 80 °C)	7.0	12.9
**Ti@Ir**[11]	0.35	272	0.023 (1.53 V)	0.50	1.86 (2 $A\,cm^{-2}$, 80 °C)	1.25	7.20
**$Sr_{0.9}Co_{0.9}Ir_{0.1}O_{3-\delta}$**[46]	0.51	240	0.029 (1.50 V)	-	-	-	-
**$Ir_{0.063}Co_{2.937}O_4$**[47]	0.24	266	0.099 (1.53 V)	-	-	-	-

Supplementary Table 6 | Benchmarking PEMWE durability under record-high cumulative charge.

Catalyst	Mem. (μm)	Temp. (°C)	Ir loading ($mg\,cm^{-2}$)	j ($A\,cm^{-2}$)	t (h)	Deg. rate ($mV\,kh^{-1}$)	$j \cdot t$ ($A\,h\,cm^{-2}$)	Mass norm. $j \cdot t$ ($A\,h\,mg^{-1}$)
**TiO_2-coated IrO_2** (This work)	**80**	**80**	**0.4**	**3**	**2600**	**0**	**7800**	**19.50**
Ir@IrO_x/m-Nb-TiO_2 [1]	127	80	0.27	2	3000	4	6000	22.22
$IrRuO_x$ [2]	90	55	3	1	5700	3.3	5700	1.90
Ir/IrO_x [3]	183	50	0.3	1.8	3000	24	5400	18.00
IrO_x/MnO_2 [4]	127	80	0.08	1.8	2700	0	4860	60.75
IrO_x [5]	183	80	0.08	1.8	2500	36.5	4500	56.25
$Ir_{0.51}Sn_{0.44}Sb_{0.05}O_x$ [6]	183	80	0.2	2	2000	18	4000	20.00
Ir_1Ru/TiO_2 [7]	101	80	0.035	2	2000	29.43	4000	114.29
Ir/Nb_2O_{5-x} [8]	127	80	3	2	2000	0	4000	1.33
Ir-Mn [9]	51	80	0.24	2	2000	32.7	4000	16.67
IrO_x [10]	127	80	0.28	2	1800	7	3600	12.86
Ti@Ir [11]	127	80	0.5	2	1800	1.252	3600	7.20
IrO_2 [12]	127	80	2	1.5	2000	7	3000	1.50
IrO_2@TaO_x@TaB [13]	127	80	0.26	2	1500	6.8	3000	11.54
$Ir_{0.7}Ru_{0.25}Nb_{0.05}O_2$ [14]	80	65	0.3	2	1500	27.8	3000	10.00
Ru_6IrO_x [15]	90	60	0.38	2	1500	2.3	3000	7.89
$IrRuO_x$ [16]	90	80	0.34	3	1000	23	3000	8.82
$IrRuO_x$ [16]	90	80	1.27	3	1000	11	3000	2.36
$IrRuO_x$ [17]	90	80	0.4	3	1000	23	3000	7.50
$IrRuO_x$ [17]	90	80	1.5	3	1000	11	3000	2.00
Ir/Pt [18]	127	80	2	1.35	2000	1.5	2700	1.35
$H_{3.8}Ir_{1-x}Ru_xO_4$ [19]	183	80	0.75	2	1280	39	2560	3.41
IrO_2+ODT@Ti_4O_7 [20]	127	80	0.2	1.5	1600	75	2400	12.00
IrO_x [21]	127	80	0.3	2	1186	2.5	2372	7.91
Sr_2CaIrO_6 [22]	50	80	0.2	2	1000	10	2000	10.00
IrO_2/TiO_2 [23]	127	80	0.5	2	1000	22.2	2000	4.00
Pt@$RuIrO_x$ [24]	127	80	0.19	1	2000	160	2000	10.53
IrO_x [25]	127	80	0.5	2	1000	22	2000	4.00
IrO_x [5]	183	80	0.08	1.8	1043	11.5	1877	23.47
$IrW_xTi_{1-x}O_2$ [26]	127	80	0.4	1.5	1200	16.7	1800	4.50
IrO_x/t-ZrO_2 [27]	127	80	0.1	1	1600	6.25	1600	16.00
$Mn_{0.15}Ir_{0.85}O_2$ [28]	51	80	0.8	2	800	19.4	1600	2.00
IrO_x/$K_2Ti_8O_{17}$ [29]	80	80	0.3	3	510	60	1530	5.10
IrO_2 [30]	127	80	1	1	1500	10.6	1500	1.50
IrO_2 [31]	175	80	1.99	1	1440	6.2	1440	0.72
Pt@$Re_{0.024}Ir_2$ [32]	51	80	0.1	1	1300	28	1300	13.00
Ru_2IrO_x [33]	127	80	1	1	1200	54.7	1200	1.20
$Mn_{0.1}Ir_{0.9}O_2$ [34]	127	80	2.5	1	1200	17.5	1200	0.48
$H_{3.8}Ir_{1-x}Ru_xO_4$ [19]	183	80	0.75	1	1160	25.8	1160	1.55
Pt@$Re_{0.024}Ir_2$ [32]	51	80	0.2	1	1100	26	1100	5.50
$IrRuO_x$@IrRu [35]	127	80	0.25	2	550	7.52	1100	4.40
IrO_2@TaO_x@TaB [13]	127	80	0.26	1	1000	2.6	1000	3.85
IrO_x-3Nd [36]	127	80	2	1	1000	55.8	1000	0.50
$IrO_x(OH)_y(H_2O)_n$ [37]	127	80	0.3	1	1000	58	1000	3.33
Ir@CeO_2 [38]	127	80	0.3	1	1000	4.85	1000	3.33
Ir/IrO_x-SO_3H [39]	51	80	1	1	1000	17	1000	1.00
IrO_2 [40]	–	80	0.84	2	500	4.12	1000	1.19
IrO_2 [41]	90	80	0.34	1	1000	0	1000	2.94
$IrRuO_x$ [16]	90	80	0.34	1	1000	15	1000	2.94
LaIr-Co_3O_4 [42]	51	80	0.2	1	1000	95	1000	5.00
IrO_2/Ti [43]	127	80	0.12	1	1000	27	1000	8.33
$IrRuO_x$ [17]	90	80	0.4	1	1000	15	1000	2.50

Supplementary Table 7 | Electrochemical metrics for IrO_2 reference and ALD-7 anodes at 3.0 A cm^{-2}.

@3.0 A cm^{-2}	IrO_2 ref (127μm)			ALD-7 (127μm)			ALD-7 (80μm)		
	BoT	EoT	Δ	BoT	EoT	Δ	BoT	EoT	Δ
Voltage (V)	1.984	2.014	+0.030	1.939	1.933	-0.006	1.824	1.814	-0.010
Tafel slope (mV/dec)	36.9	51.0	+14.1	44.5	44.0	-0.5	45.3	45.7	+0.4
$\eta_{overall}$ (mV)	553.9	584.3	+30.4	509.3	502.9	-6.4	394.2	383.9	-10.3
η_{ohmic} (mV)	356.4	357.6	+1.2	345.1	345.0	-0.1	216.4	202.8	-13.6
$\eta_{kinetic}$ (mV)	102.6	123.0	**+20.4**	115.5	123.8	**+8.3**	116.5	122.9	**+6.4**
η_{mass} (mV)	94.9	103.7	**+8.8**	48.6	34.0	**-14.6**	61.2	58.1	**-3.1**

Supplementary Table 8 | Quantitative comparison of anode properties and mass-transport overpotential.

Anode	Wettability (DSA, deg)		Pore structure (MIP)			η_{mt} at BoT (mV)
	Air bubble	Water drop	Porosity (%)	Pore area (m^2 g^{-1})	avr. Pore size (nm)	
IrO_2 ref	142.7	119.7	47.2	0.914	12.45	94.9
ALD-7	150.3	112.8	50.8	0.971	12.66	48.6
ALD effect (Δ%)	+5.33	-5.76	+7.63	+6.24	+1.69	-48.79

Supplementary Table 9 | Correlation between 2D porosity with mass-transport overpotential of anodes.

Anode	TEM-derived 2D porosity (%)			η_{mt} (mV)		
	BoT	EoT	Δ	BoT	EoT	Δ
IrO_2 ref	12.4	8.8	-3.6	94.9	103.7	+8.8
ALD-7	13	12.6	-0.4	48.6	34.0	-14.6
ALD effect (Δ%)	+4.84	+43.18	-88.89	-48.79	-67.21	-265.9

Supplementary Table 10 | BET surface area and pore analysis of IrO_x reference and a-ALD-3 catalysts.

Catalysts	IrO_x ref	a-ALD-3
Surface area ($a_{s,\mathrm{BET}}$) (m^2 g^{-1})	92.989	41.704
Pore volume ($p/p_0 = 0.990$) (cm^3 g^{-1})	0.1872	0.1794
Mean pore diameter (nm)	8.0545	17.204

Supplementary Table 11 | AFM measurements for IrO_x reference and a-ALD-3 anodes.

AFM Measurement	IrO_x ref	a-ALD-3
Roughness [Sa] (nm)	73.9	106.6
Stiffness (N m^{-1})	10.3	12.3
Adhesion Energy (aJ)	47.9	69.4

Supplementary Table 12 | Statistical metrics of Ir loading from X-ray fluorescence (XRF) mapping across the 100 cm^2 membrane electrode assembly (MEA).

**XRF Measurement**	**100 cm^2 MEA with ALD-7 anode**	
**Axis**	**Machine direction (MD)**	**Transverse direction (TD)**
**Avg. Ir-Lα Intensity (kcps)**	122.2	122.2
**Standard deviation**	1.36	3.35
**Relative standard deviation**	1.12	2.74

Supplementary Table 13 | XRF-measured Ir loading across nine sub-regions of the 100 cm^2 MEA.

**Area No.**	**Ir loading ($mg\,cm^{-2}$)**	**Area No.**	**Ir loading ($mg\,cm^{-2}$)**	**Area No.**	**Ir loading ($mg\,cm^{-2}$)**
**1**	0.421	**2**	0.414	**3**	0.395
**4**	0.422	**5**	0.422	**6**	0.414
**7**	0.411	**8**	0.422	**9**	0.416

4. Supplementary References

(S1) Ni, J. *et al.* Heterointerface anchored Ir with localized strong orbital coupling for durable proton exchange membrane water electrolysis. *Angew. Chem. Int. Ed.* **64**, e202509985 (2025).

(S2) Siracusano, S. *et al.* Degradation issues of PEM electrolysis MEAs. *Renew. Energy* **123**, 52–57 (2018).

(S3) Mirshekari, G. *et al.* High-performance and cost-effective membrane electrode assemblies for advanced proton exchange membrane water electrolyzers: Long-term durability assessment. *Int. J. Hydrog. Energy* **46**, 1526–1539 (2021).

(S4) Li, A. *et al.* Atomically dispersed hexavalent iridium oxide from MnO_2 reduction for oxygen evolution catalysis. *Science* **384**, 666–670 (2024).

(S5) Yu, H. *et al.* Nano-size IrO_x catalyst of high activity and stability in PEM water electrolyzer with ultra-low iridium loading. *Appl. Catal. B* **239**, 133–146 (2018).

(S6) Venkatesan, S. *et al.* Rapid scalable one-step production of catalysts for low-iridium content proton exchange membrane water electrolyzers. *Adv. Energy Mater.* **15**, 2401659 (2025).

(S7) Li, S. *et al.* Embedded Ir-Ru single-atom alloy with self-limiting motifs for sustainable proton exchange membrane water electrolysis. *Adv. Mater.* **38**, e07340 (2026).

(S8) Shi, Z. *et al.* Enhanced acidic water oxidation by dynamic migration of oxygen species at the Ir/Nb_2O_{5-x} catalyst/support interfaces. *Angew. Chem. Int. Ed.* **61**, e202212341 (2022).

(S9) Wang, S. *et al.* Strong heteroatomic bond-induced confined restructuring on Ir-Mn intermetallics enable robust PEM water electrolyzers. *Angew. Chem. Int. Ed.* **64**, e202420470 (2025).

(S10) Zhang, M. *et al.* Tunnel-structured IrO_x unlocks catalytic efficiency in proton exchange membrane water electrolyzers. *Nat. Commun.* **16**, 7608 (2025).

(S11) Yang, J. *et al.* Ultrastable Ti@Ir core-shell catalyst with low iridium loading for water electrolysis at industrial-level current density. *Chem. Eng. J.* **506**, 160118 (2025).

(S12) Zhang, S. *et al.* Catalyst layers subjected to sequential cation exchange and thermal annealing for efficient and durable proton exchange water electrolysis. *ACS Sustain. Chem. Eng.* **13**, 15363–15371 (2025).

(S13) Wang, Y. *et al.* Supported IrO_2 nanocatalyst with multilayered structure for proton exchange membrane water electrolysis. *Adv. Mater.* **36**, 2407717 (2024).

(S14) Wang, H. *et al.* Stabilizing bulk lattice oxygen via the enhancement of Ir/Ru–O bonds for stable oxidation catalysts in acidic media. *Appl. Catal. B* **371**, 125219 (2025).

(S15) Qiu, C. *et al.* Low-iridium stabilized ruthenium oxide anode catalyst for durable proton-exchange membrane water electrolysis. *Nat. Nanotechnol.* **20**, 1787–1795 (2025).

(S16) Siracusano, S. *et al.* New insights into the stability of a high performance nanostructured catalyst for sustainable water electrolysis. *Nano Energy* **40**, 618–632 (2017).

(S17) Siracusano, S., Baglio, V., Van Dijk, N., Merlo, L. & Aricò, A. S. Enhanced performance and durability of low catalyst loading PEM water electrolyser based on a short-side chain perfluorosulfonic ionomer. *Appl. Energy* **192**, 477–489 (2017).

(S18) Selamet, Ö. F., Becerikli, F., Mat, M. D. & Kaplan, Y. Development and testing of a highly efficient proton exchange membrane (PEM) electrolyzer stack. *Int. J. Hydrog. Energy* **36**, 11480–11487 (2011).

(S19) Tang, J. *et al.* Ruthenium single-atom modulated protonated iridium oxide for acidic water

oxidation in proton exchange membrane electrolysers. *Adv. Mater.* **36**, 2407394 (2024).

(S20) Ma, S. *et al.* Engineering conductive Ti_4O_7 networks for efficient and durable low-ir-loading anode in proton exchange membrane water electrolysis. *Small* **22**, e09778 (2026).

(S21) Xie, Z. *et al.* Honeycomb-structured IrO_x foam platelets as the building block of anode catalyst layer in PEM water electrolyzer. *Angew. Chem. Int. Ed.* **64**, e202415032 (2025).

(S22) Torrero, J. *et al.* High performance and durable anode with 10-fold reduction of iridium loading for proton exchange membrane water electrolysis. *Adv. Energy Mater.* **13**, 2204169 (2023).

(S23) Liu, H. *et al.* Optimizing ionomer distribution in anode catalyst layer for stable proton exchange membrane water electrolysis. *Adv. Mater.* **36**, 2402780 (2024).

(S24) Wang, S. *et al.* Confined subnanometer amorphous $RuIrO_x$ overlayers on ultrafine pt nanowires achieve ampere-level durable PEM water electrolysis. *Adv. Mater.* **38**, e17532 (2026).

(S25) Sun, K. *et al.* Highly efficient and durable anode catalyst layer constructed with deformable hollow IrO_x nanospheres in low-iridium PEM water electrolyzer. *Angew. Chem. Int. Ed.* **64**, e202504531 (2025).

(S26) Zhao, S., Stocks, A., Rasimick, B., More, K. & Xu, H. Highly active, durable dispersed iridium nanocatalysts for PEM water electrolyzers. *J. Electrochem. Soc.* **165**, F82–F89 (2018).

(S27) Fang, S. R. *et al.* Tetragonal ZrO_2 supported low-iridium catalyst activating oxygen spillover stabilized lattice oxygen for proton exchange membrane water electrolysis. *Energy Environ. Sci.* **18**, 5470–5481 (2025).

(S28) Chang, B. *et al.* Dynamic redox induced localized charge accumulation accelerating proton exchange membrane electrolysis. *Adv. Mater.* **37**, 2405447 (2025).

(S29) Sui, J. *et al.* Defective $K_2Ti_8O_{17}$ nanorod supports enable stable high-current-density acidic water electrolysis via confinement-engineered IrO_x. *Small* **21**, 2505131 (2025).

(S30) Yang, P. *et al.* Imidazole-tailored ionomers achieve concurrent proton conduction boost and electron transport retention in the anode catalyst layer for PEM water electrolysis. *ACS Sustain. Chem. Eng.* **13**, 14693–14701 (2025).

(S31) Fouda-Onana, F. *et al.* Investigation on the degradation of MEAs for PEM water electrolysers part I: Effects of testing conditions on MEA performances and membrane properties. *Int. J. Hydrog. Energy* **41**, 16627–16636 (2016).

(S32) Liang, J. *et al.* Rhenium-doping to promote structural evolution of metallic iridium to oxides on platinum nanowire bundles for acidic oxygen evolution. *Angew. Chem. Int. Ed.* **64**, e202512317 (2025).

(S33) Zhao, L. *et al.* Wrinkle-induced strain activates a rapid deprotonation pathway in Ru_2IrO_x for efficient acidic water electrolysis. *Adv.Funct. Mater.* **36**, e15512 (2026).

(S34) Gao, H. *et al.* Activation of iridium site based on IrO_2 catalysts towards highly stable PEM water electrolyzer. *Chem. Eng. Sci.* **302**, 120912 (2025).

(S35) Wu, H. *et al.* Electron-enriched iridium active centers via spontaneous core-shell architecture engineering for efficient and durable water oxidation catalysis. *J. Energy Chem.* **110**, 751–759 (2025).

(S36) Zhang, N. *et al.* Local oxygen vacancy-mediated oxygen exchange for active and durable acidic water oxidation. *Angew. Chem. Int. Ed.* **64**, e202503246 (2025).

(S37) Huang, R. *et al.* $Na_2Ir(OH)_6$-derived hollandite-type iridium (hydr) oxides as efficient and durable electrocatalysts for low-iridium PEM electrolyzers. *Int. J. Hydrog. Energy* **190**, 152209 (2025).

(S38) Mao, X. *et al.* Ultralow-iridium oxygen evolution catalyst with dual-site oxide pathway for proton exchange membrane water electrolysis. *Nano Lett.* **25**, 16253–16261 (2025).

(S39) Li, J. *et al.* Surface sulfonic-group bonded oxygen evolution catalyst for proton exchange membrane water electrolysis. *Nat. Commun.* **16**, 9910 (2025).

(S40) Wang, L. *et al.* Oxygen vacancy-mediated oxide pathway mechanism in proton-exchange membrane water electrolysis. *Adv. Funct. Mater.* **36**, e16646 (2026).

(S41) Siracusano, S. *et al.* The influence of iridium chemical oxidation state on the performance and durability of oxygen evolution catalysts in PEM electrolysis. *J. Power Sources* **366**, 105–114 (2017).

(S42) Wei, Z. *et al.* Lanthanum-assisted lattice anchoring of iridium in Co_3O_4 for efficient oxygen evolution reaction in low-iridium water electrolysis. *Nat. Commun.* **16**, 8145 (2025).

(S43) Rozain, C., Mayousse, E., Guillet, N. & Millet, P. Influence of iridium oxide loadings on the performance of PEM water electrolysis cells: Part II–advanced oxygen electrodes. *Appl. Catal. B* **182**, 123–131 (2016).

(S44) Nga Ngo (Sarah Ngo), T. H., Love, J. & O'Mullane, A. P. Investigating the influence of amorphous/crystalline interfaces on the stability of IrO_2 for the oxygen evolution reaction in acidic electrolyte. *ChemElectroChem* **10** (2023).

(S45) van der Merwe, M. *et al.* Unravelling the mechanistic complexity of the oxygen evolution reaction and Ir dissolution in highly dimensional amorphous hydrous iridium oxides. *Energy Environ. Sci.* **18**, 1214–1231 (2025).

(S46) Guo, H. *et al.* Ex situ reconstruction-shaped Ir/CoO/perovskite heterojunction for boosted water oxidation reaction. *ACS Catal.* **13**, 5007–5019 (2023).

(S47) Hua, K. *et al.* Catalytic activity of nanometer-sized $Ir–O_x$ catalysts with different coordination numbers for electrocatalytic oxygen evolution. *ACS Appl. Nano Mater.* **7**, 487–497 (2024).
