# Supplementary material for: Dual-interface stabilization of low-iridium anodes for durable proton exchange membrane water electrolysis
Source: Nat Commun. 2026 Jul 4;17:6818. doi: 10.1038/s41467-026-75113-6 (PMC13389522; doi:10.1038/s41467-026-75113-6)
Supplement: Supplementary file 2 — Description of Additional Supplementary Files [file 41467_2026_75113_MOESM2_ESM.pdf]

Description of Additional Supplementary Files

File Name: Supplementary Data 1

Description: Atomic coordinates of the optimized ionomer adsorption models (associated with Fig. 5d,e,f, and Supplementary Fig. 31, and Fig. 32).
