# Supplementary material for: Dual-interface stabilization of low-iridium anodes for durable proton exchange membrane water electrolysis
Source: Nat Commun. 2026 Jul 4;17:6818. doi: 10.1038/s41467-026-75113-6 (PMC13389522; doi:10.1038/s41467-026-75113-6)
Supplement: Supplementary file 4 — Transparent Peer Review file [file 41467_2026_75113_MOESM4_ESM.pdf]

nature portfolio

Peer Review File

Dual-interface stabilization of low-iridium anodes for durable proton exchange membrane water electrolysis at 3 A cm^{-2}

Corresponding Author: Dr Jai Hyun Koh

This file contains all reviewer reports in order by version, followed by all author rebuttals in order by version.

Version 0:

Reviewer comments:

Reviewer #1

(Remarks to the Author)
Recommendation: Minor Revision
Overview: The manuscript entitled "Highly durable proton exchange membrane water electrolysis at 3 A cm-2 via dual-interface stabilization of low-iridium anodes" reports a well-designed study on TiO2-modified IrO2 catalysts using ALD. The authors demonstrate impressive durability (up to 2600 h at 3 A cm-2) at low Ir loading and combine comprehensive characterization with DFT analysis to elucidate the stabilization mechanism. The work is of high relevance to PEMWE development and industrial implementation. Overall, the manuscript merits publication after minor revisions.
Comment 1. The comparison of catalytic performance with prior literature is currently focused mainly on durability metrics. It would strengthen the manuscript to include a more explicit comparison of activity (e.g., ECSA-normalized activity) with representative state-of-the-art catalysts, or to better position the system within the activity–stability trade-off.
Comment 2. The description of roll-to-roll compatibility may lead to ambiguity. While the electrode fabrication is demonstrated via roll-to-roll coating, the TiO2 layer is deposited by powder ALD. The authors are encouraged to clarify the scalability of the ALD process and its integration with continuous manufacturing.
Comment 3. The chemical nature of the deposited Ti species requires clearer definition. At low ALD cycles, the Ti appears to be highly dispersed rather than forming a continuous TiO2 layer. Additional clarification on whether the Ti exists as TiO2, suboxides, or interfacial Ti–O–Ir species would improve the mechanistic interpretation.
Comment 4. Relatedly, the manuscript consistently refers to "TiO2 coating," whereas the DFT models and experimental observations suggest the presence of interfacial Ti species. The authors are encouraged to distinguish between surface TiO2 overlayer and interfacial Ti configurations, supported by appropriate spectroscopic discussion.
Comment 5. The description of the DFT methodology, particularly the Bader charge analysis, is somewhat brief. Additional calculation details should be provided.
Comment 6. The discussion of mass-transport improvement remains largely qualitative. If possible, the authors could provide more quantitative evidence or clearer linkage between pore structure/wettability changes and transport-related overpotential.

Reviewer #2

(Remarks to the Author)
This manuscript presents a dual-interface stabilization strategy in which TiO2 is introduced onto IrO2 by atomic layer deposition to construct a low-iridium anode for PEM water electrolysis. The work addresses an important topic and shows promising durability at high current density, while the demonstration of roll-to-roll coating further enhances its practical relevance. Overall, this study is interesting and potentially valuable. However, several issues related to interfacial interpretation, catalyst stability, and computational analysis should be addressed before the manuscript can be considered further.
1.The differences in contact angle are relatively small, and the interfacial behavior of the catalyst layer is likely strongly influenced by the ionomer. Please clarify more explicitly the specific contribution of the catalyst itself to the observed interfacial properties.
2.The manuscript indicates that TiO2 modification reduces the oxidation state of Ir. Please discuss the functional significance of this change more clearly. In particular, does it help suppress over-oxidation or Ir dissolution under high-current-density operation?

3.The claim that TiO_2 modification suppresses lattice oxygen participation during the OER is important, but direct evidence is currently insufficient. Additional experimental or theoretical support would strengthen this conclusion.
4.The post-reaction analysis mainly focuses on pore structure and interfacial properties. A more complete discussion of changes in the chemical environment of Ir and the evolution of active sites during operation would further strengthen the mechanistic understanding.
5.TiO_2 modification may also introduce unfavorable effects, such as decreased conductivity and partial blockage of active sites. Since performance deterioration is observed at higher ALD cycle numbers, please discuss more carefully the trade-off between activity and stability as the coating thickness increases.
6.During the stability test, repeated cycles of voltage increase and recovery are observed on a timescale of roughly 100 h. Please clarify the origin of these fluctuations. For a PEMWE system, electrolyte depletion should not be the dominant reason for this behavior. It would also be helpful to explain why the reference IrO_2 anode exhibits a much larger degradation amplitude than the modified samples.
7.The presentation of the performance data in Fig. 2a requires clarification. The stated 20-30 mV reduction in iR-free cell voltage at 3.0 A cm^{-2} relative to the reference IrO_2 anode is not clearly reflected in the figure. Please improve the figure and ensure consistency between the text and the plotted data. In addition, the statement that the catalyst meets the U.S. DOE 2026 durability target should be supported by a clear quantitative comparison, as this point is not fully convincing based on the current data. Since high-current operation is critical for practical electrolyzers, it would also be valuable to provide performance data at current densities above 3 A cm^{-2}, if available.
8.The DFT model used in Fig. 4e, based on a three-layer IrO_2 slab with a single Ti atom introduced at the surface, requires further justification. Please clarify the basis for constructing this model and provide the optimized structures in the Supporting Information.
9.Although characterization after 1000 h is presented, longer-term post-test characterization would provide stronger evidence for structural stability under extended operation. If available, please provide morphological or structural data after longer durability tests. In addition, ICP-MS analysis of the electrolyte to quantify dissolved Ir would further support the long-term stability claim.
10.The manuscript shows that the 80 μm Nafion membrane performs better than the 127 μm membrane. Please explain why the 127 μm membrane was still selected for the performance evaluation of the 100 cm^2 MEA.

Reviewer #3

(Remarks to the Author)
This study addresses durability/cost challenges of iridium-based anodes in high-current PEM water electrolyzers via a dual-interface strategy using TiO_2 ALD on IrO_2. It enables 2600h stable operation at 3.0 A cm^{-2} with near-zero voltage decay at 0.4 mg cm^{-2} Ir loading—vastly outperforming bare IrO_2. Mechanistically, Ti-O-Ir coupling inhibits Ir dissolution; TiO_2 coating boosts ionomer interaction/mass transport. Roll-to-roll compatible, it cuts capital costs by 22% and projects \$3.2/kg H_2 at 1000 MW yr^{-1}. Critically, it offers a generalizable principle to extend electrolyzer lifetime and reduce noble-metal loading in electrochemical technology. Therefore, I would like to recommend the publication of this work, with the following revisions suggested.
1.TEM-EDS only detects Ir deposited in the membrane, not dissolved Ir lost in the anode effluent. Moreover, Ir dissolution in a three-electrode system does not necessarily reflect that in a real membrane electrode assembly. Direct ICP analysis of the outlet water from PEMWE operation is needed to confirm that the TiO_2 coating truly suppresses total Ir dissolution rather than merely altering its transport and deposition behavior.
2.In the Ir 4f XPS fitting in Supplementary Fig. 19a, the spin–orbit splitting between the Ir 4f 7/2 and 4f 5/2 doublets is not held constant, violating a fundamental physical constraint. This inconsistency undermines the reliability of the derived Ir oxidation states and the conclusions drawn from the XPS analysis.
3.The main text mentioned the Ti-induced electronic modulation suppresses Ir over-oxidation, however post characterization of the Ir oxidation state after long-term operation is missing.
4.The authors attribute the initial voltage reduction of TiO_2 coated anodes primarily to improved mass transport, while noting that partial coverage of Ir active sites by TiO_2 would otherwise be detrimental. However, the manuscript does not provide electrochemically active surface area measurements or specific activity for the different catalysts.
5.Static contact angle measurements alone are insufficient to prove enhanced bubble detachment under operating conditions. Operando optical or high-speed imaging is needed to directly verify that the TiO_2 coating reduces bubble accumulation and thereby lowers mass transport overpotential at 3.0 A cm^{-2}.
6.The pore size distributions derived from BET shows a dVp/ddp at ~12 nm, while the pre size distributions derived from mercury intrusion porosimetry reveal that ALD-7 have an obvious higher pore area. Why the pore size distributions from these two methods show different results?
7.Page 5, Line105, "the TiO_2 coating modulates catalyst surface characteristics and thereby establishes a uniform pore architecture and favorable wettability across the electrodes." However, it's hard to say that there is a big difference between the contact angle of 119.7° and 112.8°, which may have minor influence on water spreading and gas bubble detachment.
8.The authors claimed that the wettability contrast facilitates water spreading and gas bubble detachment. However, there seems to be no experiment evidence to support this viewpoint. More experiments like bubble visualization of catalytic layer are suggested to be performed to support this viewpoint.
9.ALD-7 shows exhibits a uniform Ti distribution at IrO_2 surface, but Fig. 2a shows the catalyst with best activity is ALD-3. Why chooses ALD-7 as the target sample? And as the cycle numbers changes, what dominates the activity change?
10.Standard tests with RDE should be performed to fully evaluate the performance and mechanism of the catalysts. After ALD cycles, the ECSA may decrease and the inherent activity may change. These elements should be considered.
11.From TEM images, Ti atoms are located onto IrO_2 surface and form heterogeneous interfaces. However, the DFT-calculated atomic model uses a Ti doped IrO_2 model, which may be not match with reality.

12.The authors claims that Ti induced electronic electronic stabilization of Ir mitigates over-oxidation-driven dissolution. However, there seems to be no evidence that IrO_2 dissolve because over-oxidation and Ti doping suppress this process. XPS or XAFS after OER should be provided and analyzed.
13.TiO_2 coating enhances ionomer adsorption capacity and binding strength, therefore stabilizing the catalyst surface and catalyst-layer architecture. However, several literatures have pointed out that strong adsorption of anionic sulfonate groups can lead to the sedimentation of catalysts in catalyst inks, and block active sites (Nat. Catal. 2023, 6 (5), 392-401.). Why in this work, the binding of catalysts and anionic sulfonate groups have the opposite effect?
14.Supplementary Fig.19 a, the fitting parameters should be provided. The X-axis of XPS results should be uniform.

Version 1:

Reviewer comments:

Reviewer #1

(Remarks to the Author)
I have reviewed the authors' revised manuscript and their detailed point-by-point response to my previous comments. I am pleased to conclude that all of my concerns have been addressed.

Recommendation: Accept

Reviewer #2

(Remarks to the Author)
The authors have made substantial revisions and provided additional experimental results and analyses that significantly improve the overall quality and comprehensiveness of the manuscript. The work is now suitable for publication in its current form. Nevertheless, a few issues remain that should be further clarified or improved:
1. The XPS results and the increased oxygen-vacancy formation energy primarily indicate enhanced lattice stability. However, they do not directly demonstrate the suppression of lattice oxygen participation during the OER process. The authors are encouraged to provide more direct evidence, such as isotope-labeling experiments, to substantiate this claim, or alternatively, to moderate the corresponding conclusions in the manuscript.
2. Supplementary Table 5 should be expanded to include more representative studies on Ir-based catalysts. In addition, important benchmarking parameters, such as Ir loading and overpotential, should be incorporated to enable a more comprehensive comparison.
3. The white labels in Fig. 6b and 6c are difficult to distinguish from the background and can be easily overlooked. The authors are advised to replace them with black or other higher-contrast colors to improve readability.

Reviewer #3

(Remarks to the Author)
The authors have addressed my questions.

Version 2:

Reviewer comments:

Reviewer #2

(Remarks to the Author)
I recommend acceptance of this work for publication.

Open Access This Peer Review File is licensed under a Creative Commons Attribution 4.0 International License, which permits use, sharing, adaptation, distribution and reproduction in any medium or format, as long as you give appropriate credit to the original author(s) and the source, provide a link to the Creative Commons license, and indicate if changes were made.
In cases where reviewers are anonymous, credit should be given to 'Anonymous Referee' and the source.
The images or other third party material in this Peer Review File are included in the article's Creative Commons license, unless indicated otherwise in a credit line to the material. If material is not included in the article's Creative Commons license and your intended use is not permitted by statutory regulation or exceeds the permitted use, you will need to obtain permission directly from the copyright holder.
To view a copy of this license, visit https://creativecommons.org/licenses/by/4.0/

Note to all reviewers: As requested by the editor, we have removed the techno-economic assessment (former Fig. 6b,c and the associated discussion) from the revised manuscript. Accordingly, reviewer comments that pertain to the techno-economic analysis are no longer applicable to the revised version. All remaining comments have been addressed in full as detailed below.

Response to the Reviewer #1:

Recommendation: Minor Revision

Overview: The manuscript entitled "Highly durable proton exchange membrane water electrolysis at 3 $A\,cm^{-2}$ via dual-interface stabilization of low-iridium anodes" reports a well-designed study on TiO_2-modified IrO_2 catalysts using ALD. The authors demonstrate impressive durability (up to 2600 h at 3 $A\,cm^{-2}$) at low Ir loading and combine comprehensive characterization with DFT analysis to elucidate the stabilization mechanism. The work is of high relevance to PEMWE development and industrial implementation. Overall, the manuscript merits publication after minor revisions.

We sincerely thank Reviewer #1 for the positive assessment of our work and for recognizing the relevance of the dual-interface stabilization strategy to PEMWE development and industrial implementation. We are grateful for the constructive comments that have helped us further strengthen the manuscript. We carefully addressed all detailed comments provided and revised the manuscript and SI accordingly.

1. The comparison of catalytic performance with prior literature is currently focused mainly on durability metrics. It would strengthen the manuscript to include a more explicit comparison of activity (e.g., ECSA-normalized activity) with representative state-of-the-art catalysts, or to better position the system within the activity–stability trade-off.

We thank the reviewer for this valuable suggestion. We agree that positioning the catalytic activity relative to prior literature is important. In response, we have quantitatively compared the ECSA-normalized current density (j_{ECSA}) at 1.55 V vs. RHE with representative state-of-the-art Ir-based catalysts reported in the literature (*Angew. Chem. Int. Ed.* **61**, e202212341 (2022); *Nat. Nanotechnol.* **20**, 1787–1795 (2025); *Energy Environ. Sci.* **18**, 5470–5481 (2025)). The ALD-7 catalyst exhibited a j_{ECSA} of 0.022 $mA\,cm^{-2}$, which is comparable to reported values of 0.0275, 0.021, and 0.0325 $mA\,cm^{-2}$ from these studies, respectively. In contrast, when evaluated in terms of mass-normalized charge density ($j \cdot t$ per mg_{Ir}, reflecting cumulative charge throughput during long-term operation), the present catalyst delivered a significantly higher value of 19.5 $kAh\,mg^{-1}$, exceeding those reported in the same studies (1.33, 7.89, and 16.0 $kAh\,mg^{-1}$, respectively). These results demonstrate that the TiO_2 modification preserves the intrinsic catalytic activity at a level comparable to state-of-the-art Ir-based catalysts, while markedly enhancing catalyst utilization and durability. Consequently, the present system effectively shifts the conventional activity–stability trade-off toward a more favorable regime.

The experimental details and corresponding data have been added as Supplementary Note 2 (page 4), Supplementary Fig. 12 (page 17), and Supplementary Tables 4 and 5 (page 42) of the revised SI:

"**Supplementary Note 2 |** RDE-based evaluation of ECSA and intrinsic activity

Rotating disk electrode (RDE) measurements were conducted to quantitatively assess the intrinsic catalytic activity of the TiO_2-coated IrO_2 catalysts and to benchmark them against state-of-the-art Ir-based systems. In particular, the electrochemically active surface area (ECSA) and ECSA-normalized current density

(j_{ECSA}) were determined to decouple intrinsic activity from geometric and morphological effects.

Catalyst inks (10 mg of catalyst, 300 μL of deionized water, 900 μL of *n*-propanol, and 5.2 μL of 20 wt% Nafion) were ultrasonicated for 20 min, and 5.5 μL was drop-cast onto a glassy carbon electrode. Electrochemical measurements were performed in a three-electrode configuration with a Ag/AgCl reference electrode and a Pt counter electrode in 0.5 M H_2SO_4 at a rotation rate of 1,600 rpm. The double-layer capacitance (C_{dl}) was obtained from cyclic voltammetry (0.2–1.2 V, 5-100 mV s^{-1}) with Δj extracted at 1.1 V, and ECSA was calculated using C_s = 40 μF cm^{-2}. Electrochemical impedance spectroscopy (EIS, 1.4 V, 100 kHz-100 Hz) and linear sweep voltammetry (LSV, 1.1-1.6 V, 5 mV s^{-1}) were performed to evaluate catalytic activity, and potentials were converted to the RHE scale after iR correction.

The ECSA of bare IrO_2 was determined to be 418.3 cm^2 and decreased systematically with increasing TiO_2 ALD cycle number. The corresponding values for ALD-3, ALD-5, ALD-7, and ALD-10 were 188.5, 156.4, 144.4, and 88.1 cm^2, respectively. This trend indicates progressive surface coverage by TiO_2 and reduced accessible active sites. Despite the substantial decrease in ECSA, the intrinsic activity, represented by j_{ECSA} at 1.55 V, remains relatively stable from ALD-3 to ALD-7. Bare IrO_2 shows a value of 0.040 mA cm^{-2}, while ALD-3 to ALD-7 maintain comparable values ranging from 0.018 to 0.022 mA cm^{-2}, and ALD-10 exhibits a markedly reduced value of 0.009 mA cm^{-2}. ALD-3 to ALD-7 retain intrinsic activity within the typical range of state-of-the-art Ir-based catalysts, while delivering the highest mass-normalized charge among reported systems, indicating substantially enhanced durability without compromising activity. (Supplementary Fig. 12 and Supplementary Tables 4 and 5)."

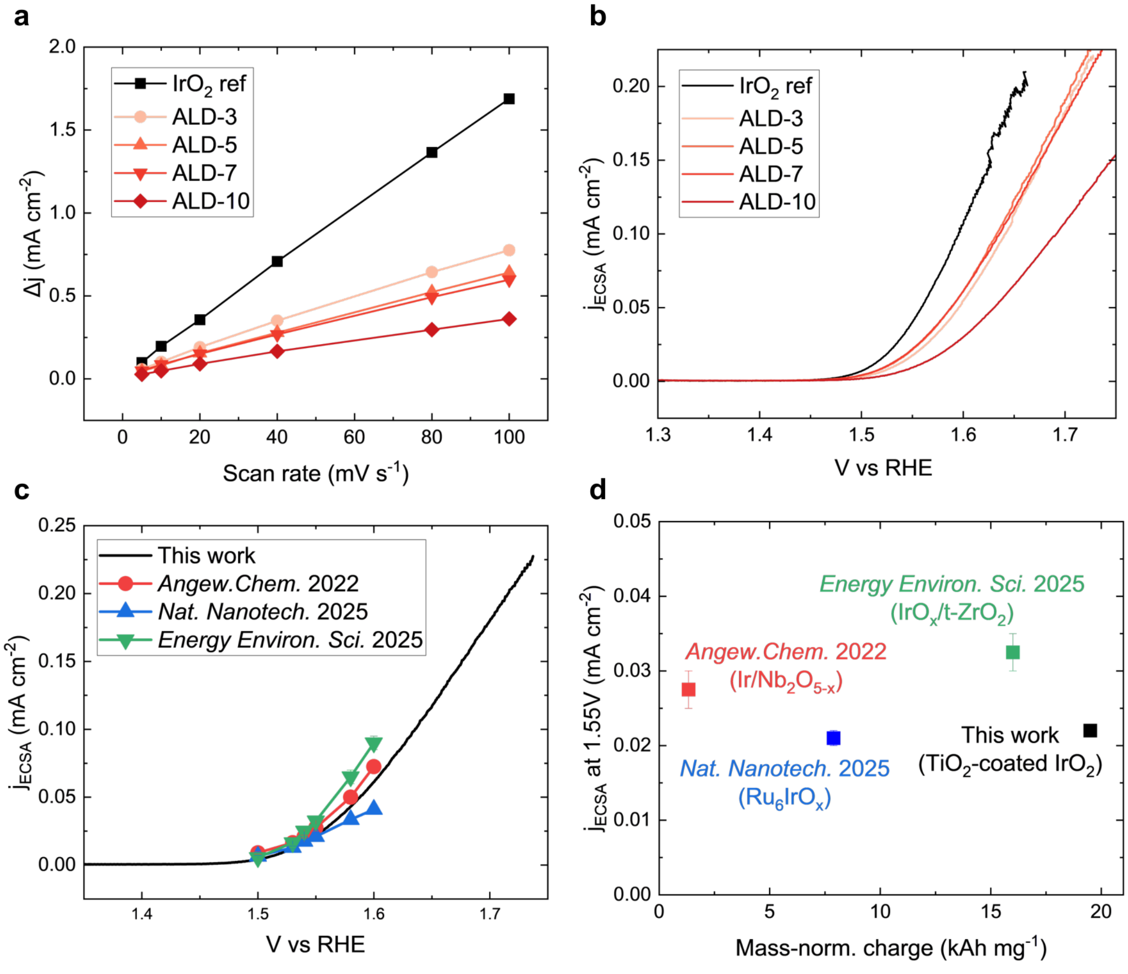

Supplementary Fig. 12 | RDE-derived intrinsic activity with benchmarking against state-of-the-art catalysts. **a,** Determination of ECSA from double-layer capacitance (C_{dl}) obtained from the linear relationship between scan rate and capacitive current density difference (Δj), where the slope is highest for the pristine IrO_2 and decreases progressively with increasing TiO_2-ALD cycle number. **b,** ECSA-normalized activity of the bare IrO_2 and TiO_2-coated catalysts, showing that ALD-3, ALD-5, and ALD-7 exhibit only a moderate decrease relative to bare IrO_2, whereas ALD-10 shows a substantial decline. **c,** Comparison of the ECSA-normalized activity of ALD-7 from this work with reported state-of-the-art Ir-based catalysts, demonstrating that the activity of ALD-7 falls within the range of literature values.[14–16] **d,** Correlation between ECSA-normalized activity and mass-normalized charge density, showing that this work achieves higher mass-normalized charge than state-of-the-art reports, indicating enhanced catalyst utilization and superior durability under high-current operation.

References:

(14) Shi, Z. *et al.* Enhanced acidic water oxidation by dynamic migration of oxygen species at the Ir/Nb_2O_{5-x} catalyst/support interfaces. *Angew. Chem. Int. Ed.* **61**, e202212341 (2022).
(15) Qiu, C. *et al.* Low-iridium stabilized ruthenium oxide anode catalyst for durable proton-exchange membrane water electrolysis. *Nat. Nanotechnol.* **20**, 1787–1795 (2025).
(16) Fang, S. R. *et al.* Tetragonal ZrO_2 supported low-iridium catalyst activating oxygen spillover stabilized lattice oxygen for proton exchange membrane water electrolysis. *Energy Environ. Sci.* **18**, 5470–5481 (2025).

Supplementary Table 4 | ECSA-normalized activity of TiO_2-coated IrO_2 catalysts.

**Catalyst**	C_{dl} mF cm^{-2}	**ECSA** cm^2	j_{ECSA} **at 1.55 V** mA cm^{-2}
**IrO_2 ref**	16.73	418.3	0.040
**ALD-3**	7.54	188.5	0.018
**ALD-5**	6.26	156.4	0.022
**ALD-7**	5.78	144.4	0.022
**ALD-10**	3.52	88.1	0.009

Supplementary Table 5 | Benchmarking ECSA-normalized activity of Ir-based catalysts.

**Catalyst**	j_{ECSA} **at 1.55 V** mA cm^{-2}	**Deg. Rate** mV kh^{-1}	**Mass-norm. charge** kAh mg^{-1}	**Reference**
**TiO_2-coated IrO_2**	0.022	0	19.5	This work
**Ir/Nb_2O_{5-x}**	0.0275	0	1.33	*Angew. Chem. Int. Ed.* **61**, e202212341 (2022).
**Ru_6IrO_x**	0.021	2.3	7.89	*Nat. Nanotechnol.* **20**, 1787–1795 (2025).
**IrO_x/t-ZrO_2**	0.0325	6.25	16.0	*Energy Environ. Sci.* **18**, 5470–5481 (2025).

We have also incorporated the reviewer's suggestion by adding the following statement to the main text on page 6:

"The electrochemical performance of the TiO_2-modified catalysts was assessed at two complementary levels through rotating disk electrode (RDE) measurements for intrinsic activity and PEMWE single-cell

testing at 80 ºC with an Ir loading of 0.4 mg cm^{-2} for electrode-level performance under industrially relevant conditions. RDE measurements revealed that the electrochemically active surface area (ECSA) decreased progressively with increasing ALD cycles, consistent with a partial loss of accessible surface area upon TiO_2 coating. Nevertheless, the intrinsic catalytic activity (j_{ECSA}) of ALD-3 to ALD-7 remained comparable to that of state-of-the-art Ir-based catalysts, whereas ALD-10 exhibited inferior intrinsic activity due to excessive surface coverage at higher cycle numbers (Supplementary Fig. 12, Supplementary Tables 4 and 5, and Supplementary Note 2)."

2. The description of roll-to-roll compatibility may lead to ambiguity. While the electrode fabrication is demonstrated via roll-to-roll coating, the TiO_2 layer is deposited by powder ALD. The authors are encouraged to clarify the scalability of the ALD process and its integration with continuous manufacturing.

We thank the reviewer for raising this point and agree that the distinction between the two process steps warrants clarification. The TiO_2 deposition and the electrode fabrication are decoupled, sequential processes. ALD is applied to the catalyst powder as an upstream batch modification step, after which the coated powder is formulated into a catalyst ink and processed through continuous roll-to-roll slot-die coating. The two steps therefore do not need to be integrated inline, and each can be scaled independently.

Regarding the scalability of the powder ALD step itself, the deposition was performed using a commercial rotary ALD reactor (Atomic Shell, CN1) with batch loadings of 1.8–5.0 g. Rotary and fluidized-bed ALD reactor configurations have been demonstrated at industrial scales with throughput reaching kilograms and are already employed for commercial powder coating applications in battery and catalyst manufacturing (*Mater. Today Chem.* **14,** 100183 (2019)). In addition, the low number of ALD cycles required in our process (7 cycles) further reduces processing time and cost relative to thick-film ALD coatings. From a practical perspective, the required catalyst quantities for large-scale MEA production can be accommodated within the demonstrated scaling range of such ALD systems. Moreover, the additional processing cost is expected to be minor compared to the iridium material cost. These considerations support the scalability and practical feasibility of the proposed approach.

In the revised manuscript, we have added clarifying text to the roll-to-roll discussion to explicitly distinguish the two process steps and to address ALD scalability. The revised text on page 17 is as follows, with changes underlined:

"Finally, the enhanced colloidal stability of the TiO_2-coated catalyst enabled direct translation of the materials design to industrially relevant roll-to-roll processing, yielding uniform, defect-free coatings with precise thickness control. This process compatibility is further supported by the fact that the TiO_2 ALD step is applied to the catalyst powder prior to ink formulation, rendering it fully decoupled from the continuous coating process. In addition, the availability of ALD reactors with kilogram-scale powder throughput further underscores the scalability of the approach.[49]...

49. van Ommen, J. R. & Goulas, A. Atomic layer deposition on particulate materials. *Mater. Today Chem.* **14,** 100183 (2019)."

3. The chemical nature of the deposited Ti species requires clearer definition. At low ALD cycles, the Ti appears to be highly dispersed rather than forming a continuous TiO_2 layer. Additional clarification on whether the Ti exists as TiO_2, suboxides, or interfacial Ti−O−Ir species would improve the mechanistic interpretation.

We thank the reviewer for this insightful comment. We agree that the chemical nature of the deposited Ti species evolves with ALD cycle number and that a clearer distinction is warranted.

(1) Structural evolution of Ti species with ALD cycles: We distinguish between the structural regimes as a function of ALD cycle number. As shown in Supplementary Fig. 1, a continuous TiO_2 overlayer is formed only at higher ALD cycles (≥15 cycles), whereas in the range primarily investigated in this work (ALD-3 to ALD-7), the Ti species are highly dispersed on the IrO_2 surface rather than forming a continuous film (Fig. 1b–d).

(2) Chemical state of Ti species: The chemical state of these dispersed Ti species was examined by XPS (Supplementary Fig. 2, relocated from the original Supplementary Fig. 19 to the catalyst characterization section to align with the revised manuscript flow). The Ti $2p_{3/2}$ spectra (Supplementary Fig. 2c) show binding energies at approximately 458.5–459.0 eV, which are characteristic of Ti^{4+} species and are consistent with TiO_2-like oxide environments.

(3) Interfacial electronic coupling via Ti−O−Ir motifs: Multiple spectroscopic analyses indicate that the Ti species interact strongly with the IrO_2 surface. Specifically, systematic shifts in the Ir electronic structure with increasing ALD cycles are observed in the Ir 4f XPS spectra (Fig. 4a), Ir L_3-edge XANES (Fig. 4b), and EXAFS (Fig. 4c), suggesting electronic coupling between Ti and Ir. These results are consistent with the formation of interfacial Ti−O−Ir motifs rather than a physically separated TiO_2 layer. If the deposited phase were bulk-like TiO_2 with no electronic interaction, the Ir 4f position would remain unchanged. This interpretation is further supported by DFT calculations (Fig. 4d–f), which show that Ti incorporation at the IrO_2 surface induces charge redistribution and modifies the local electronic structure of adjacent Ir atoms, consistent with the experimentally observed shifts.

Based on these combined results, we clarify that, in the low-cycle regime relevant to this work, the deposited Ti exists predominantly as highly dispersed Ti^{4+} oxide species that are electronically coupled to IrO_2 through interfacial Ti−O−Ir configurations, rather than as a continuous TiO_2 overlayer.

In the revised manuscript, we have added relevant text to the catalyst characterization section on pages 3–4 to clarify the chemical nature of the dispersed Ti species. The added text is underlined below:

"...Transmission electron microscopy (TEM) and scanning transmission electron microscopy–energy-dispersive X-ray spectroscopy (STEM–EDS) analyses indicate that ALD-7 exhibits a uniform Ti distribution at the IrO_2 surface without the formation of a clearly resolved continuous layer (Fig. 1b–d), whereas samples prepared with higher ALD cycle numbers display distinct core–shell structures (i.e., IrO_2@TiO_2, Supplementary Fig. 1). <u>XPS analysis further confirms that the dispersed Ti species exhibit Ti $2p_{3/2}$ binding energies of 458.5–459.0 eV, characteristic of Ti^{4+} in TiO_2-like oxide environments</u> (Supplementary Fig. 2)..."

4. Relatedly, the manuscript consistently refers to "TiO_2 coating," whereas the DFT models and experimental observations suggest the presence of interfacial Ti species. The authors are encouraged to distinguish between surface TiO_2 overlayer and interfacial Ti configurations, supported by appropriate spectroscopic discussion.

We thank the reviewer for this suggestion that is closely related to *Point 3 from Reviewer #1*. We agree that the terminology should more accurately reflect the nature of the deposited Ti species, which varies with ALD cycle number as revealed by the spectroscopic and microscopic evidence. At low ALD cycle numbers (e.g., ALD-7), Ti exists primarily as highly dispersed interfacial species rather than as a continuous bulk-like TiO_2 overlayer. STEM-EDS shows uniform Ti distribution without a resolvable shell structure, while XPS and XANES/EXAFS reveal direct electronic modulation of Ir sites through interfacial Ti−O−Ir coupling. In contrast, distinct core–shell morphologies become evident only at higher ALD cycle numbers (Supplementary Fig. 1). These observations collectively indicate that the term "TiO_2 coating" is an operational simplification. In the revised manuscript, we have adopted the following terminology convention to address this point:

(a) "TiO_2-coated IrO_2" or "TiO_2-modified IrO_2" is retained as the general descriptor for the catalyst system, as TiO_2 is the ALD-deposited material and this terminology is consistent with the ALD process description.

(b) When discussing the mechanistic origin of electronic stabilization, we now explicitly refer to "interfacial Ti−O−Ir species" or "Ti−O−Ir linkages" rather than "TiO_2 coating" to accurately reflect the spectroscopic and microscopic evidence.

(c) When discussing ionomer–catalyst interactions and surface wettability, we use "TiO_2-like surface" to describe the modified surface character at moderate ALD cycle numbers, consistent with the surface property saturation observed at 5–7 cycles (Supplementary Figs. 7 and 8).

This distinction has been applied throughout the revised manuscript. Representative examples of the revised terminology include, with changes underlined:

Page 3:
"...The formation of an <u>interfacial Ti−O−Ir species at the IrO_2 surface</u> suppresses excessive Ir oxidation under highly anodic potentials,..."

"...The outer <u>TiO_2-modified</u> surface further enhances interfacial compatibility with the ionomer,..."

Page 12:
"...These spectroscopic trends demonstrate that <u>interfacial Ti−O−Ir coupling, rather than passive coverage by a bulk TiO_2 phase,</u> lowers the Ir valence state."

Page 16:
"Collectively, the Ti-induced electronic modulation <u>via interfacial Ti−O−Ir coupling</u> not only suppresses Ir over-oxidation but also strengthens the electrostatic interaction with the ionomer,..."

Page 18:
"...<u>At moderate ALD cycle numbers, the deposited Ti exists as interfacial Ti−O−Ir species that</u> simultaneously modulate the electronic structure of the catalyst surface and the ionomer–catalyst interactions..."

5. The description of the DFT methodology, particularly the Bader charge analysis, is somewhat brief. Additional calculation details should be provided.

We thank the reviewer for this helpful comment. To improve clarity and reproducibility, we have expanded the description of the DFT methodology in the revised manuscript. Specifically, we now provide additional details on the PAW potentials used, including the treatment of semi-core electrons, and we have clarified the procedure for atomic charge analysis using the grid-based Bader method.

The construction of the all-electron charge density and the definition of atomic charges are now explicitly described, and the relevant reference for the Bader analysis has been added. We believe these additions improve the transparency and completeness of the computational methodology. The relevant text is now added in the Methods section on page 23 of the revised manuscript as follows, with changes underlined:

"The generalized gradient approximation with the Perdew-Burke-Ernzerhof functional was adopted as the exchange-correlation treatment.[55] The electron–ion interactions were described using PAW potentials that explicitly included semi-core electrons for both iridium ($5d^8 6s^1$) and titanium ($3p^6 3d^2 4s^2$), while oxygen atoms were treated with their ground-state valence configuration ($2s^2 2p^4$)...."

"...Atomic charge states were analyzed using the grid-based Bader charge analysis method.[57] The all-electron charge density was reconstructed by combining valence and core charge densities, and the atomic charge of each element was defined as the difference between the number of electrons in the neutral atom and the integrated Bader charge...."

6. The discussion of mass-transport improvement remains largely qualitative. If possible, the authors could provide more quantitative evidence or clearer linkage between pore structure/wettability changes and transport-related overpotential.

We thank the reviewer for this valuable comment and agree that a more quantitative correlation between catalyst-layer properties and mass-transport behavior strengthens the manuscript. In the revised manuscript, we have added a new Supplementary Table 8 that directly correlates initial catalyst-layer properties (wettability and pore structure) with the mass-transport overpotential (η_{mt}). Compared to the IrO_2 reference, the ALD-7 anode exhibits modest changes in wettability (air-bubble contact angle: +5.33%; water contact angle: –5.76%) and structural properties (porosity: +7.63%; pore area: +6.24%; average pore size: +1.69%). These changes collectively result in a substantial reduction in mass-transport overpotential (-48.8%) as shown in Supplementary Table 8 below:

Supplementary Table 8 | Quantitative comparison of anode properties and mass-transport overpotential.

Anode	Wettability (DSA, deg)		Pore structure (MIP)			η_{mt} at BoT (mV)
	Air bubble	Water drop	Porosity (%)	Pore area ($m^2\ g^{-1}$)	avr. Pore size (nm)	
IrO_2 ref	142.7	119.7	47.2	0.914	12.45	94.9
ALD-7	150.3	112.8	50.8	0.971	12.66	48.6
ALD effect (Δ%)	+5.33	-5.76	+7.63	+6.24	+1.69	-48.79

We further established a quantitative correlation by analyzing the evolution of TEM-derived 2D porosity and the corresponding change in $\Delta\eta_{mt}$ during prolonged operation at 3 A cm^{-2}. The IrO_2 reference anode exhibits a significant decrease in porosity (12.4% to 8.8%) after 1,000 h, accompanied by an increase in η_{mt} (+8.8 mV). In contrast, the ALD-7 anode shows minimal porosity loss (13.0% to 12.6%) after 1,100 h operation and, notably, a decrease in η_{mt} (–14.6 mV) as shown in the newly added Supplementary Table 9 and Supplementary Fig. 22 below:

Supplementary Table 9 | Correlation between 2D porosity with mass-transport overpotential of anodes.

**Anode**	**TEM-derived 2D porosity (%)**			**η_{mt} (mV)**		
	**BoT**	**EoT**	**Δ**	**BoT**	**EoT**	**Δ**
IrO_2 ref	12.4	8.8	-3.6	94.9	103.7	+8.8
ALD-7	13	12.6	-0.4	48.6	34.0	-14.6
ALD effect (Δ%)	+4.84	+43.18	-88.89	-48.79	-67.21	-265.9

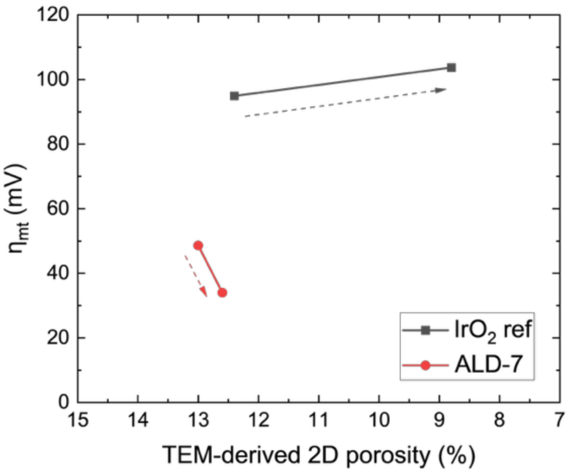

Supplementary Fig. 22 | Correlation between 2D porosity with mass-transport overpotential of anodes. Comparison of changes in η_{mt} at 3 A cm^{-2} during prolonged operation as a function of TEM-derived 2D porosity for IrO_2 reference and ALD-7 anodes. The IrO_2 reference exhibits a pronounced porosity loss (12.4% to 8.8%) after 1,000 h operation, accompanied by an increase in η_{mt} (+8.8 mV). In contrast, the ALD-7 anode maintains its porosity (13.0% to 12.6%) after 1,100 h operation and shows a decrease in η_{mt} (-14.6 mV). These results indicate that preserving catalyst-layer architecture is critical for mitigating mass-transport losses under high-current operation.

These results indicate that mass-transport behavior is governed by both the initial pore structure, which establishes baseline transport pathways, and the ability to preserve the catalyst-layer architecture under high-current operation. Among these, the dominant effect arises from the suppression of pore collapse, which mitigates gas accumulation and transport resistance, whereas the initial wettability and pore structure contribute secondarily to improved interfacial characteristics. Furthermore, the observed decrease in η_{mt} for the ALD-7 anode suggests a dynamic stabilization of the catalyst layer during operation, likely associated with improved ionomer distribution and maintained transport pathways. This behavior contrasts with the progressive structural degradation observed in the reference IrO_2 sample.

We have incorporated these quantitative analyses and discussions into the revised manuscript and SI to clarify the linkage between catalyst-layer structure and transport-related overpotential. The relevant discussions are added to the revised manuscript as follows, with the changes underlined.

Page 9, in the overpotential decomposition section:

"...To quantify this effect, the total overpotential (η_{total}) at 3.0 A cm^{-2} was deconvoluted into mass-transport (η_{mt}), kinetic (η_{kin}), and ohmic (η_{ohm}) contributions at both the beginning-of-test (BoT) and the end-of-test (EoT) states (Fig. 2e,f, Supplementary Fig. 14 and Supplementary Table 7). At BoT, the ALD-7 anode exhibited a significantly lower η_{mt} (48.6 mV) compared to the reference IrO_2 anode (94.9 mV), which can be attributed to its improved wettability and more open pore structure (Supplementary Table 8)..."

Page 11, in the post-mortem porosity analysis section:

"...Quantitative porosity analysis derived from TEM images also shows a decrease in porosity of the reference IrO_2 anode from 12.4% to 8.8% after operation with a 127 μm membrane, while the porosity of the ALD-7 anode changed marginally from 13.0% to 12.6% under the same conditions, and remained largely preserved at 12.1% even after extended operation for 2,600 h with an 80 μm membrane (Fig. 3i). This structural evolution directly correlates with the change in mass-transport overpotential, where the reference anode exhibits an increase in η_{mt} due to pore collapse, whereas the ALD-7 anode shows a decrease, consistent with preserved transport pathways (Supplementary Table 9 and Supplementary Fig. 22)..."

Response to the Reviewer #2:

This manuscript presents a dual-interface stabilization strategy in which TiO_2 is introduced onto IrO_2 by atomic layer deposition to construct a low-iridium anode for PEM water electrolysis. The work addresses an important topic and shows promising durability at high current density, while the demonstration of roll-to-roll coating further enhances its practical relevance. Overall, this study is interesting and potentially valuable. However, several issues related to interfacial interpretation, catalyst stability, and computational analysis should be addressed before the manuscript can be considered further.

We thank Reviewer #2 for the thoughtful evaluation and for acknowledging the practical relevance of our dual-interface stabilization strategy and roll-to-roll demonstration. We appreciate the detailed comments regarding interfacial interpretation, catalyst stability, and computational analysis, which have prompted us to substantially strengthen the mechanistic discussion and add new experimental and computational evidence. We have carefully addressed each point with additional data, clarified analyses, and expanded discussion as detailed below.

1. The differences in contact angle are relatively small, and the interfacial behavior of the catalyst layer is likely strongly influenced by the ionomer. Please clarify more explicitly the specific contribution of the catalyst itself to the observed interfacial properties.

We thank the reviewer for this important point. We agree that the ionomer strongly influences the macroscopic interfacial behavior of the catalyst layer, and that the electrode-level contact angle difference alone does not constitute the primary evidence for our claims. The specific contribution of the catalyst surface is not to directly control electrode-level wettability, but rather to modulate how the ionomer adsorbs and organizes at the catalyst–ionomer interface. Several independent measurements support this distinction:

(1) Intrinsic surface contribution (ionomer-free): Coffee-ring analyses and contact angle measurements on model films (Supplementary Figs. 7 and 8) demonstrate that TiO_2 coating progressively increases surface hydrophilicity up to a saturation plateau beyond 5–7 ALD cycles. This effect is a direct consequence of the modified catalyst surface chemistry, measured independently of the ionomer.

(2) Catalyst-mediated ionomer reorganization: The TiO_2-modified surface governs ionomer distribution and adhesion strength. AFM adhesion energy maps of the electrodes show progressively greater ionomer coverage with increasing ALD cycle number (Supplementary Fig. 10 and Supplementary Table 3). ITC measurements reveal an extended adsorption regime and higher cumulative heat for TiO_2-coated catalysts (Fig. 5c), while ^{19}F NMR confirms an increase in the adsorbed ionomer fraction from 25% to 45% for ALD-7 (Supplementary Fig. 30). DFT calculations further show that the enhanced binding originates from a charge redistribution across the Ti−O−Ir linkages, which creates electron-deficient Ti sites and thus strengthens electrostatic stabilization of sulfonate groups at bridge sites (–1.42 eV vs. –1.00 eV on pristine IrO_2). Such changes in ionomer adsorption and sulfonate-group organization at the catalyst surface are expected to influence the local interfacial wetting behavior (*J. Phys. Chem. B* **122**, 6107–6119 (2018)). Accordingly, the observed wetting behavior can serve as indirect signatures of catalyst-induced ionomer reorganization at the catalyst–ionomer interface.

(3) Functional consequence at the pore level: Rather than the macroscopic contact angle, the operationally relevant outcome is the preservation of pore architecture during prolonged operation. Mercury intrusion porosimetry shows a more uniform pore size distribution and higher porosity for ALD-7 anodes

(Fig. 1f), and post-mortem TEM-based porosity analysis (Fig. 3i) confirms that the ALD-7 anode retained its pore volume (13.0% to 12.6%) while the reference IrO_2 anode suffered pronounced pore collapse (12.4% to 8.8%) after 1,000 h.

Taken together, these results clarify that the TiO_2-modified catalyst surface modulates the catalyst–ionomer interfacial interaction. Although the resulting electrode-level contact angle differences are relatively small, they nevertheless provide supporting evidence that the underlying catalyst surface modification alters the organization of ionomer sulfonate groups at the catalyst-ionomer interface. This catalyst-mediated ionomer reorganization is expected to promote a more uniform and stable pore structure under operation. This mechanistic chain establishes a clear link between catalyst surface chemistry and the observed electrode-level interfacial behavior. In the revised manuscript, we have tempered the language linking contact angle directly to bubble detachment and reframed wettability as one indicator within a broader suite of interfacial evidence. The relevant text on page 5 of the revised manuscript is shown below with changes underlined:

"...Drop shape analysis further confirms that the ALD-7 anode exhibits modestly higher hydrophilicity and greater aerophobicity than the reference IrO_2 anode (Supplementary Fig. 11). Although the difference in electrode-level contact angle is small, such wettability changes are consistent with altered adsorption and organization of ionomer sulfonate groups at the catalyst–ionomer interface induced by the underlying surface modification.[32–34] This ionomer reorganization, rather than the macroscopic wettability change alone, contributes to preserving a uniform pore architecture and a favorable interfacial environment during prolonged operation."

2. The manuscript indicates that TiO_2 modification reduces the oxidation state of Ir. Please discuss the functional significance of this change more clearly. In particular, does it help suppress over-oxidation or Ir dissolution under high-current-density operation?

We thank the reviewer for this comment and agree that the functional significance of the reduced Ir oxidation state deserves a more explicit discussion. The mechanistic chain connecting electronic modulation to operational durability can be summarized as follows:

(1) Baseline oxidation state reduction: XPS Ir 4f spectra show a progressive shift toward lower binding energies with increasing TiO_2-ALD cycle number (Fig. 4a), and Ir L_3-edge XANES reveals a systematic decrease in white-line intensity (Fig. 4b). Both observations indicate that interfacial Ti−O−Ir coupling lowers the average Ir valence state. DFT Bader charge analysis confirms substantial charge reduction at Ir atoms adjacent to Ti (0.81 e for Ti-attached vs. 1.29 e for pristine IrO_2, Fig. 4f).

(2) Suppression of over-oxidation under anodic conditions: Under the highly oxidative potentials of OER at 3.0 $A\,cm^{-2}$, Ir atoms in pristine IrO_2 are driven toward higher oxidation states (Ir^{5+}/Ir^{6+}), which are thermodynamically unstable and constitute the precursors to dissolution. The Ti-induced electronic downshift is expected to increase the energetic barrier for reaching these over-oxidized states, thereby widening the thermodynamically stable potential–pH window, as supported by the Pourbaix analysis in Supplementary Fig. 26c. Consistently, DFT calculations further show that increasing Ti content raises the oxygen-vacancy formation energy (Supplementary Fig. 26b), reflecting a stabilized oxygen sublattice with reduced susceptibility to lattice-oxygen participation during OER (i.e., a pathway closely linked to transient Ir dissolution).

(3) Direct evidence of suppressed dissolution: Post-mortem cross-sectional TEM reveals pronounced Ir migration into the membrane for the reference IrO_2 anode after 1,000 h at 3.0 A cm^{-2}, whereas the ALD-7 anode shows no detectable Ir migration after 1,100 h (Fig. 3a–d). ICP-MS analysis of PEMWE effluent water and independent ICP–OES analysis from three-electrode stability tests consistently confirms that the TiO_2 coating suppresses Ir dissolution (Supplementary Figs. 15–19). We further substantiated this mechanism by adding post-operation XPS characterization of the ALD-7 anode after long-term operation as shown below in the newly added Supplementary Fig. 27. The Ir 4f binding energy of the operated ALD-7 anode remains close to the pre-operation values, suggesting that the Ir electronic state remains comparatively stable even after prolonged 3.0 A cm^{-2} operation. This result provides supporting evidence that the electronic stabilization mechanism remains functional throughout the durability test and is not merely an initial-state effect.

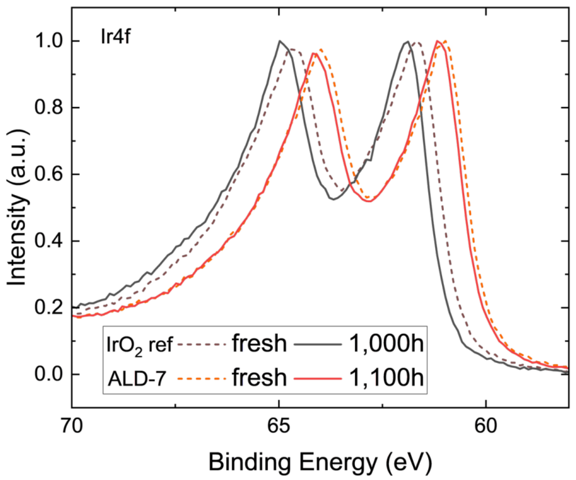

Supplementary Fig. 27 | XPS Ir 4f spectra of ALD-7 anodes after operation at 3.0 A cm^{-2}. The Ir 4f XPS spectra of both the IrO_2 reference (black) and ALD-7 (red) anodes show negligible changes between the fresh and post-operation states (1,000–1,100 h at 3.0 A cm^{-2}), while ALD-7 consistently maintains a lower binding energy than the IrO_2 reference, indicating that the initial electronic structure is well preserved under prolonged high-current operation.

Collectively, these results consistently support the interpretation that Ti-induced electronic stabilization mitigates over-oxidation-driven dissolution. In the revised manuscript, we have expanded the discussion on pages 12–13 to explicitly describe this functional chain as follows, with the added text underlined:

"...These findings indicate that interfacial Ti−O−Ir coupling stabilizes the oxygen sublattice and is expected to suppress lattice-oxygen participation during OER.[26,28,29,44] Specifically, the lower baseline Ir valence state increases the potential required to access highly oxidized Ir species (Ir^{5+}/Ir^{6+}) which are associated with transient dissolution under strongly anodic conditions.[13,14,22] This electronic stabilization is corroborated by the absence of detectable Ir migration in post-mortem TEM analysis (Fig. 3d), lower dissolved Ir concentrations measured by ICP-MS and ICP-OES (Supplementary Figs. 15–19), and the minimal change in the Ir 4f binding energy observed by post-operation XPS (Supplementary Fig. 27). Overall, the Ti-induced electronic stabilization of Ir mitigates over-oxidation-driven dissolution under acidic OER conditions and provides the mechanistic foundation for the enhanced durability observed in the TiO_2-modified anodes."

3. The claim that TiO_2 modification suppresses lattice oxygen participation during the OER is important, but direct evidence is currently insufficient. Additional experimental or theoretical support would strengthen this conclusion.

We thank the reviewer for this comment. We agree that the claim regarding suppressed lattice oxygen participation warrants stronger support. As discussed in our response to *Point 2 from Reviewer #2*, we have performed post-operation XPS on the ALD-7 anode after long-term testing at 3.0 A cm^{-2} (Supplementary Fig. 27). The Ir 4f binding energy of the operated anode remains close to pre-operation values, suggesting that irreversible over-oxidation of Ir is suppressed during operation. Although these measurements do not directly track lattice oxygen exchange, they are consistent with stabilization of the oxygen sublattice and suppressed lattice-oxygen-mediated degradation pathways. Ir over-oxidation is closely linked to lattice oxygen participation because oxygen-vacancy formation destabilizes the Ir coordination environment and facilitates Ir release. The preserved Ir electronic state is therefore consistent with a more stable oxygen sublattice.

This interpretation is further supported by DFT calculations showing that the oxygen-vacancy formation energy increases systematically with Ti content (Supplementary Fig. 26b), which indicates that extracting lattice oxygen from the Ti-modified surface is thermodynamically less favorable. The corresponding Pourbaix diagram (Supplementary Fig. 26c) further shows that the thermodynamically stable potential–pH window broadens upon Ti incorporation. The suppressed Ir dissolution confirmed by post-mortem TEM (Fig. 3a–d) and ICP-OES (Supplementary Figs. 16–19) provides additional indirect support, as transient Ir dissolution is frequently associated with lattice-oxygen vacancy formation. The corresponding manuscript revisions, including the tempered language, are shown in our response to *Point 2 from Reviewer #2*.

4. The post-reaction analysis mainly focuses on pore structure and interfacial properties. A more complete discussion of changes in the chemical environment of Ir and the evolution of active sites during operation would further strengthen the mechanistic understanding.

We thank the reviewer for this suggestion. This point is addressed by the new post-operation XPS data and expanded mechanistic discussion presented in our responses to *Points 2 and 3 from Reviewer #2*. Together, the post-reaction characterization now spans the chemical environment of Ir (post-operation Ir 4f XPS, Supplementary Fig. 27), structural integrity of the catalyst layer (post-mortem TEM porosity, Fig. 3i), and Ir dissolution behavior (TEM, Fig. 3a–d; ICP-MS and ICP-OES, Supplementary Figs. 15–19). These analyses collectively provide a comprehensive picture of active-site preservation during prolonged operation.

5. TiO_2 modification may also introduce unfavorable effects, such as decreased conductivity and partial blockage of active sites. Since performance deterioration is observed at higher ALD cycle numbers, please discuss more carefully the trade-off between activity and stability as the coating thickness increases.

We thank the reviewer for this important comment. We agree that excessive TiO_2 deposition may introduce unfavorable effects such as reduced electronic conductivity and partial blockage of active sites. We further elucidated this point through RDE measurements to evaluate the electrochemically active surface area (ECSA) and the ECSA-normalized activity (j_{ECSA}) at 1.55 V vs. RHE for pristine IrO_2 and TiO_2-coated catalysts with varying ALD cycles (Supplementary Table 4, Supplementary Fig. 12, and Supplementary Note 2, shown in our response to *Point 1 from Reviewer #1*). The ECSA of bare IrO_2 was measured to be 418.3 cm^2 and decreased with increasing ALD cycle number. The corresponding values for ALD-3, ALD-5, ALD-7, and ALD-10 were 188.5, 156.4, 144.4, and 88.1 cm^2, respectively. This trend indicates

progressive surface coverage by TiO_2 and reduced accessible active sites. Despite the substantial decrease in ECSA, the intrinsic activity (represented by j_{ECSA} at 1.55 V) remains relatively stable from ALD-3 to ALD-7. Bare IrO_2 exhibits a value of 0.040 mA cm^{-2}, while ALD-3, ALD-5, and ALD-7 show comparable values ranging from 0.018 to 0.022 mA cm^{-2}. ALD-10 exhibits a significantly reduced value of 0.009 mA cm^{-2}, indicating that excessive TiO_2 coverage leads to a deterioration in intrinsic catalytic activity.

In contrast, the PEMWE performance exhibits a different trend, where the iR-free cell voltage at 3.0 A cm^{-2} follows the order ALD-5 $\sim$ ALD-7 < ALD-3 < ALD-10 < IrO_2 reference (Fig. 2a). This behavior indicates that practical cell performance is governed not only by intrinsic activity but also by catalyst-layer interfacial and mass-transport properties. The deterioration at higher ALD cycle numbers (ALD-10) can be attributed to excessive surface coverage, as directly evidenced by both reduced ECSA and lowered intrinsic activity. In contrast, the slightly higher voltage of ALD-3 relative to ALD-5 and ALD-7 is attributed to insufficient interfacial modification, where the Ti coverage is too low to effectively improve catalyst-layer wettability and pore structure. As a result, mass-transport limitations are not fully alleviated, which leads to higher cell voltage at high current density.

Notably, while activity shows a clear dependence on ALD cycle number, the durability enhancement is already achieved within the low-cycle regime (ALD-3 to ALD-7), with no significant additional stability gain at higher cycle numbers. This pattern indicates that the dominant trade-off is not between activity and stability, but rather between insufficient interfacial stabilization at low coverage and excessive surface coverage at high cycle numbers. Therefore, ALD-7 represents an optimal balance, where sufficient Ti-induced interfacial effects are achieved without incurring the detrimental effects associated with overcoating, consistent with both RDE-derived intrinsic activity and full-cell PEMWE performance. We have clarified this point on page 6 of the revised manuscript as follows, with changes underlined:

"The electrochemical performance of the TiO_2-modified catalysts was assessed at two complementary levels through rotating disk electrode (RDE) measurements for intrinsic activity and PEMWE single-cell testing at 80 ºC with an Ir loading of 0.4 mg cm^{-2} for electrode-level performance under industrially relevant conditions. RDE measurements revealed that the electrochemically active surface area (ECSA) decreased progressively with increasing ALD cycles, consistent with a partial loss of accessible surface area upon TiO_2 coating. Nevertheless, the intrinsic catalytic activity (j_{ECSA}) of ALD-3 to ALD-7 remained comparable to that of state-of-the-art Ir-based catalysts, whereas ALD-10 exhibited inferior intrinsic activity due to excessive surface coverage at higher cycle numbers (Supplementary Fig. 12, Supplementary Tables 4 and 5, and Supplementary Note 2).

Under polarization testing in PEMWE single-cell measurements, all TiO_2-modified anodes (3, 5, and 7 cycles), except for ALD-10, exhibited an approximately 20–30 mV reduction in iR-free cell voltage at 3.0 A cm^{-2} compared with the reference IrO_2 anode (Fig. 2a). The observed voltage reduction was not driven by an increase in intrinsic catalytic activity, but rather by enhanced mass transport arising from improved pore structure and wettability. The slightly higher iR-free voltage of ALD-3 reflects incomplete interfacial optimization at insufficient TiO_2 coverage, whereas the inferior performance of ALD-10 reflects compromised active-site accessibility at excessive coverage. Based on the trend in iR-free voltage (ALD-5 $\sim$ ALD-7 $<$ ALD-3 $<$ ALD-10 $<$ IrO_2 reference), ALD-7 was selected as the representative anode in this study, as it provides a favorable balance between performance and durability."

6. During the stability test, repeated cycles of voltage increase and recovery are observed on a timescale of roughly 100 h. Please clarify the origin of these fluctuations. For a PEMWE system, electrolyte depletion should not be the dominant reason for this behavior. It would also be helpful to explain why the reference IrO_2 anode exhibits a much larger degradation amplitude than the modified samples.

We thank the reviewer for this insightful comment. We agree that electrolyte depletion is unlikely to be the dominant origin of the observed voltage fluctuations under PEMWE operation. Instead, the periodic voltage increase and recovery arise from reversible mass-transport limitations associated with high-current-density operation.

In this study, the durability test was conducted using a cyclic protocol consisting of galvanostatic operation at 3 $A\,cm^{-2}$ for 138 h, followed by a 24 h rest period and subsequent performance evaluation for approximately 6 h using stepwise polarization. Each cycle required approximately one week and was repeated multiple times. During prolonged high-current operation, oxygen bubble accumulation within the catalyst layer and porous transport layer can progressively hinder reactant/product transport, leading to a gradual increase in cell voltage. Upon interruption of operation (i.e., rest), these accumulated gas bubbles are released and the local water distribution is re-equilibrated, resulting in a recovery of the cell voltage. This phenomenon is commonly referred to as reversible voltage increase (*Fuel* **386**, 134344 (2025)).

The magnitude of this reversible voltage increase depends strongly on the catalyst-layer microstructure and interfacial properties. In the reference IrO_2 anode, the larger voltage fluctuation amplitude is attributed to less favorable pore structure and wettability, which exacerbate gas accumulation and transport limitations over time. As a result, both the extent of voltage rise during operation and the degree of recovery upon rest are significantly larger than those observed for the TiO_2-modified anodes (Fig. 2b).

In contrast, the TiO_2-modified anodes exhibit substantially suppressed reversible voltage increase, with a reduced amplitude of $\sim$30 mV compared to $\sim$100 mV for the reference IrO_2 anode (Fig. 2b). This behavior is consistent with the improved catalyst-layer architecture observed in this work, including preserved pore structure and more favorable interfacial wettability, which facilitate more efficient gas removal and water redistribution under high-current-density conditions. As a result, transport-related accumulation effects are mitigated, leading to a smaller voltage rise during operation and reduced fluctuation amplitude than for the reference IrO_2 anode.

In the revised manuscript, we have clarified this point by explicitly describing the origin of the reversible voltage increase in terms of mass-transport limitations associated with oxygen bubble accumulation within the catalyst layer. The revised text on page 7 is as follows, with changes underlined:

"...The reversible voltage increase is substantially suppressed upon TiO_2 coating ($\sim$30 mV) compared with that of the reference IrO_2 anode ($\sim$100 mV). This behavior is consistent with mitigation of mass-transport limitations associated with oxygen bubble accumulation within the catalyst layer, which obstructs reactant supply and hinders efficient gas removal under high-current-density operation.[35] The threefold reduction in voltage oscillation amplitude provides in situ functional evidence of suppressed bubble-induced transport limitations during 3.0 $A\,cm^{-2}$ operation, complementing the enhanced aerophobicity observed in ex situ wettability measurements (Supplementary Fig. 11)..."

7. The presentation of the performance data in Fig. 2a requires clarification. The stated 20–30 mV reduction in iR-free cell voltage at 3.0 A cm^{-2} relative to the reference IrO_2 anode is not clearly reflected in the figure. Please improve the figure and ensure consistency between the text and the plotted data. In addition, the statement that the catalyst meets the U.S. DOE 2026 durability target should be supported by a clear quantitative comparison, as this point is not fully convincing based on the current data. Since high-current operation is critical for practical electrolyzers, it would also be valuable to provide performance data at current densities above 3 A cm^{-2}, if available.

We thank the reviewer for these suggestions. We have addressed the three sub-points accordingly.

(1) Clarification of Fig. 2a: We agree that the 20–30 mV difference in iR-free cell voltage at 3.0 A cm^{-2} is difficult to discern in the original figure due to the compressed voltage scale. In the revised manuscript, we have added an inset to Fig. 2a to magnify the high-current-density region, making the iR-free voltage trend clearly observable in the order of ALD-5 $\sim$ ALD-7 $<$ ALD-3 $<$ ALD-10 $<$ IrO_2 reference. This clarified presentation also supports the selection of ALD-7 as the representative anode in this study, based on its balanced performance and durability. The revised Fig. 2 is shown below, with changes in the caption underlined:

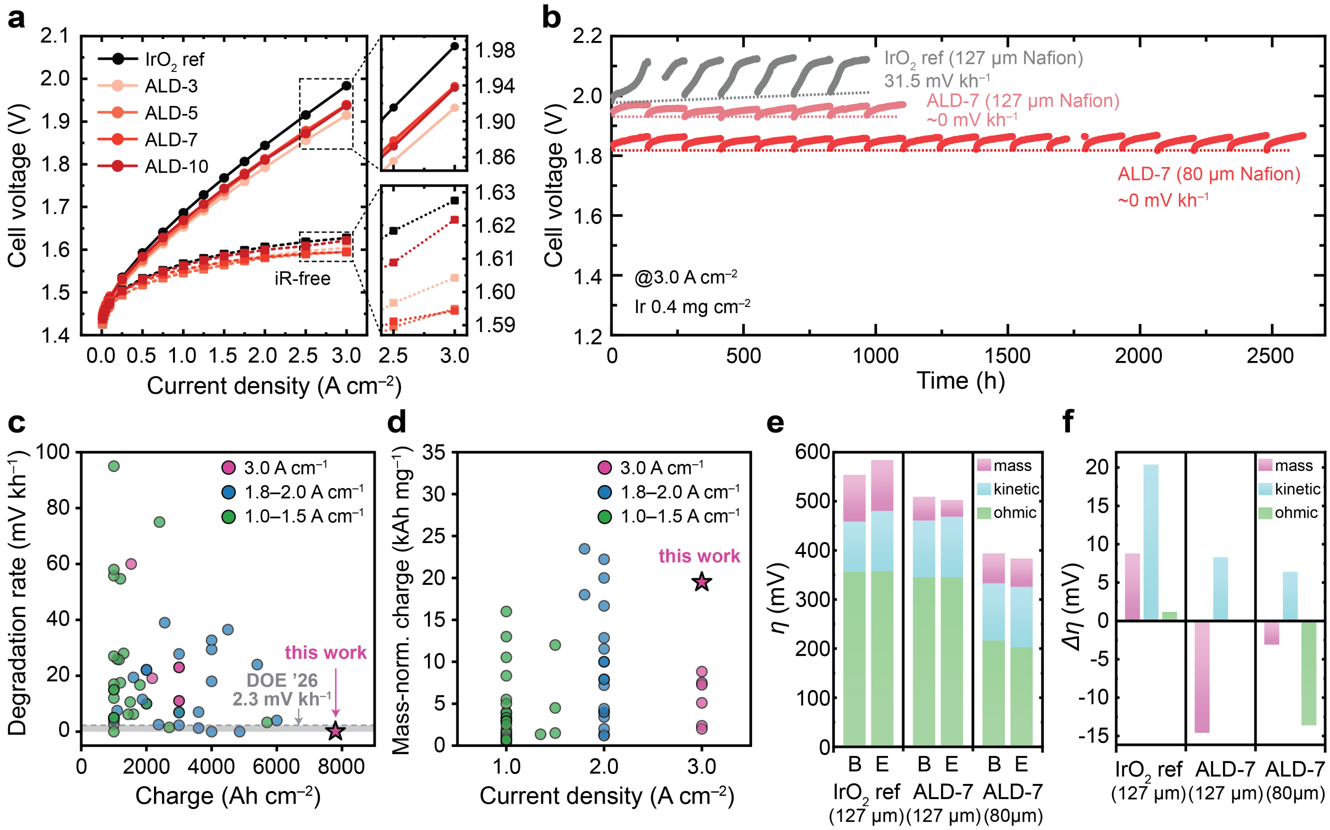

Fig. 2 | Electrochemical performance and durability of TiO_2-coated IrO_2 anodes under high-current PEMWE operation. a Polarization curves of PEMWE cells equipped with pristine IrO_2 and TiO_2-coated IrO_2 anodes. <u>The insets display magnified voltages in the range of 2.5–3.0 A cm^{-2}, highlighting the reduced cell voltage of TiO_2-coated IrO_2 anodes</u>...

(2) U.S. DOE 2026 durability target: We agree that a clear quantitative comparison is needed and have clarified the comparison with the U.S. DOE 2026 durability target by providing a quantitative evaluation of the degradation rate. The DOE target specifies a degradation rate of ≤2.3 mV kh^{-1} and emphasizes

evaluation under conditions relevant to practical operation, including dynamic operation and on–off cycling. In this work, the durability test was conducted using a cyclic protocol that includes prolonged galvanostatic operation at 3.0 A cm^{-2}, followed by rest and performance evaluation steps. Such a protocol inherently incorporates operational interruptions and recovery processes, thereby reflecting practical duty cycles relevant to real electrolyzer operation.

Under these conditions, the TiO_2-modified anode exhibited a near-zero degradation rate ($\leq$0 mV kh^{-1}, Supplementary Fig. 13) during the high-current operation segments, which is lower than the DOE 2026 target. The slightly negative value originates from a gradual reduction in mass-transport overpotential during operation. The present results therefore demonstrate stable operation within the targeted degradation range under practically relevant, dynamic operating conditions. We have clarified this point on page 3 of the revised manuscript as follows, with changes underlined:

"...As a result, the optimized anode with an Ir loading of 0.4 mg cm^{-2} sustains operation at 3.0 A cm^{-2} for 2,600 h with a near-zero voltage degradation rate. This durability at low Ir loading outperforms recently reported PEMWE systems <u>and</u> satisfies the U.S. Department of Energy (DOE) 2026 durability target <u>($\leq$2.3 mV kh^{-1})</u>..."

(3) Performance data above 3.0 A cm^{-2}: In this study, both the galvanostatic durability tests and polarization curve measurements were conducted up to 3.0 A cm^{-2}, which already exceeds the current densities employed in the majority of reported PEMWE durability studies (Supplementary Table 6). The DOE 2026 durability target itself specifies a minimum of 2.0 A cm^{-2}, which further underscores that 3.0 A cm^{-2} constitutes a stringent test condition. Evaluation at even higher current densities is an interesting direction that would require cell hardware optimized for enhanced thermal management and water delivery, and is a subject of ongoing work.

8. The DFT model used in Fig. 4e, based on a three-layer IrO_2 slab with a single Ti atom introduced at the surface, requires further justification. Please clarify the basis for constructing this model and provide the optimized structures in the Supporting Information.

We thank the reviewer for this important comment. We agree that the choice of the DFT model requires clearer justification, particularly with respect to how it represents the local coordination environment of Ti species on the IrO_2 surface.

(1) Justification of the DFT model: The DFT model used in Fig. 4e was constructed to capture the local electronic interaction between Ti species and the IrO_2 surface through Ti−O−Ir linkages. In this work, a three-layer IrO_2(110) slab with a single Ti atom introduced at the surface was employed as a simplified and representative configuration to evaluate the effect of Ti incorporation on the electronic structure of neighboring Ir atoms. We note that this model is not intended to represent the exact atomic structure of the experimentally observed system, where Ti species are highly dispersed at the surface. Rather, it provides a physically meaningful framework to isolate and quantify the local charge redistribution induced by Ti in the vicinity of Ir sites.

(2) Justification of slab thickness: The use of a three-layer IrO_2(110) slab is based on the metallic nature of IrO_2, where electronic perturbations introduced by surface species are efficiently screened within the topmost atomic layers. Because the Bader charge analysis employed in this work primarily probes short-range local coordination effects, the key electronic interactions between Ti and neighboring Ir atoms are well captured within this slab thickness. The conclusions of this work are therefore based on relative

trends in local charge redistribution rather than absolute surface energetics. This modeling approach is also consistent with prior computational studies on rutile IrO_2(110) surfaces (*J. Phys. Chem. C* **112**, 9872 (2008)).

Following the reviewer's suggestion, we have included the fully optimized atomic structures of the DFT models in the revised SI as Supplementary Fig. 25 as shown below:

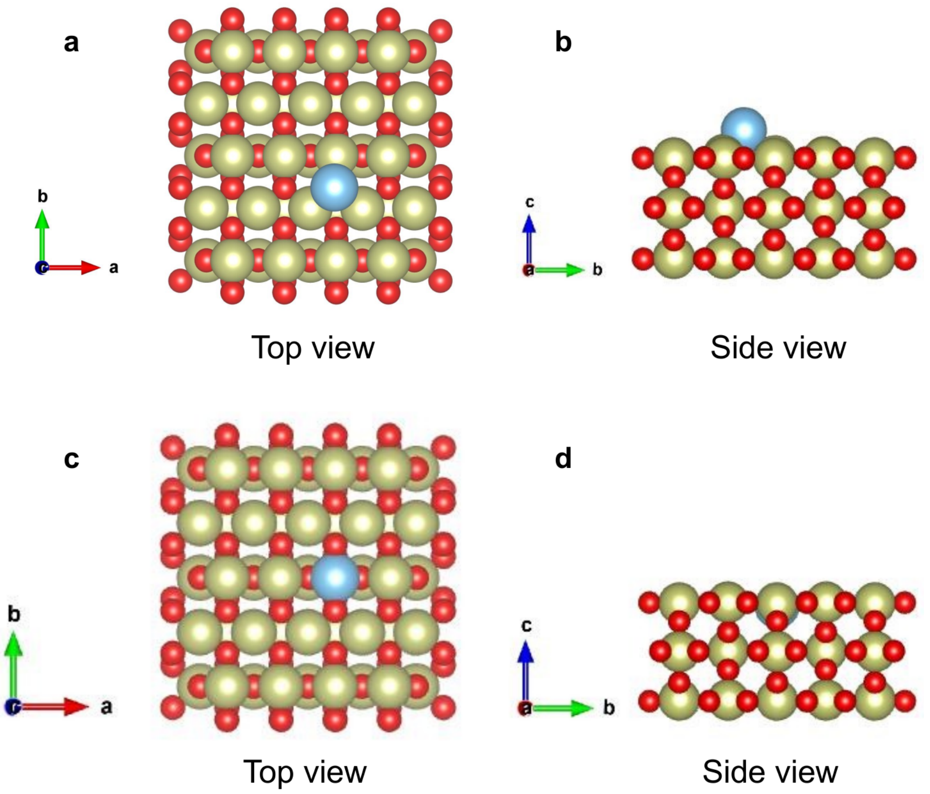

Supplementary Fig. 25 | Optimized atomic structures of the Ti-attached and Ti-doped IrO_2(110) surface models. a, b, Optimized atomic structures of the Ti-attached IrO_2(110) surface in top **(a)** and side **(b)** views. **c, d,** Optimized atomic structures of the Ti-doped IrO_2(110) surface in top **(c)** and side **(d)** views.

We have also clarified this point on page 12 of the revised manuscript as follows:

"To model the Ti−O−Ir interfacial interaction, we employed both Ti-attached and Ti-substituted IrO_2(110) surface models in density functional theory (DFT) calculations. These configurations represent limiting cases of local coordination environments at the interface, enabling systematic evaluation of the electronic modulation of Ir sites induced by Ti species. While the "Ti-attached" model reflects surface-bound Ti species observed by TEM, the Ti-substituted model ("Ti-doped") represents a more strongly coupled configuration that may arise from interfacial mixing during post-annealing. Accordingly, these models are intended to capture local electronic interactions rather than to reproduce the exact atomic structure of the catalyst (Supplementary Fig. 25). Based on these models, we first examined the electronic perturbation induced by Ti at the IrO_2 surface..."

9. Although characterization after 1000 h is presented, longer-term post-test characterization would provide stronger evidence for structural stability under extended operation. If available, please provide morphological or structural data after longer durability tests. In addition, ICP-MS analysis of the electrolyte to quantify dissolved Ir would further support the long-term stability claim.

We thank the reviewer for this valuable suggestion.

(1) Longer-term post-test characterization: We further supported the long-term structural stability through additional post-test characterization of the ALD-7 anode after extended operation (2,600 h) with an 80 μm membrane. Cross-sectional TEM imaging was performed on the same specimen used for the 2,600 h durability test, and the resulting images were processed by the same image-segmentation procedure described for the original Fig. 3i and Supplementary Fig. 18 to extract TEM-derived 2D porosity values. The analysis shows that the porosity of the catalyst layer remains largely preserved, with a TEM-derived 2D porosity of 12.1%, compared to 13.0% for the fresh electrode and 12.6% after 1,100 h of operation, as shown in the revised Fig. 3i and Supplementary Fig. 21. This result indicates that no significant pore collapse occurs even after prolonged high-current operation and thus provides further evidence for the structural robustness of the TiO_2-modified anode.

The newly acquired 2,600 h dataset has been incorporated into both the revised Fig. 3i and Supplementary Fig. 21. Specifically, Fig. 3i has been updated to include the ALD-7 (80 μm) / 2,600 h dataset as follows with changes in the caption underlined:

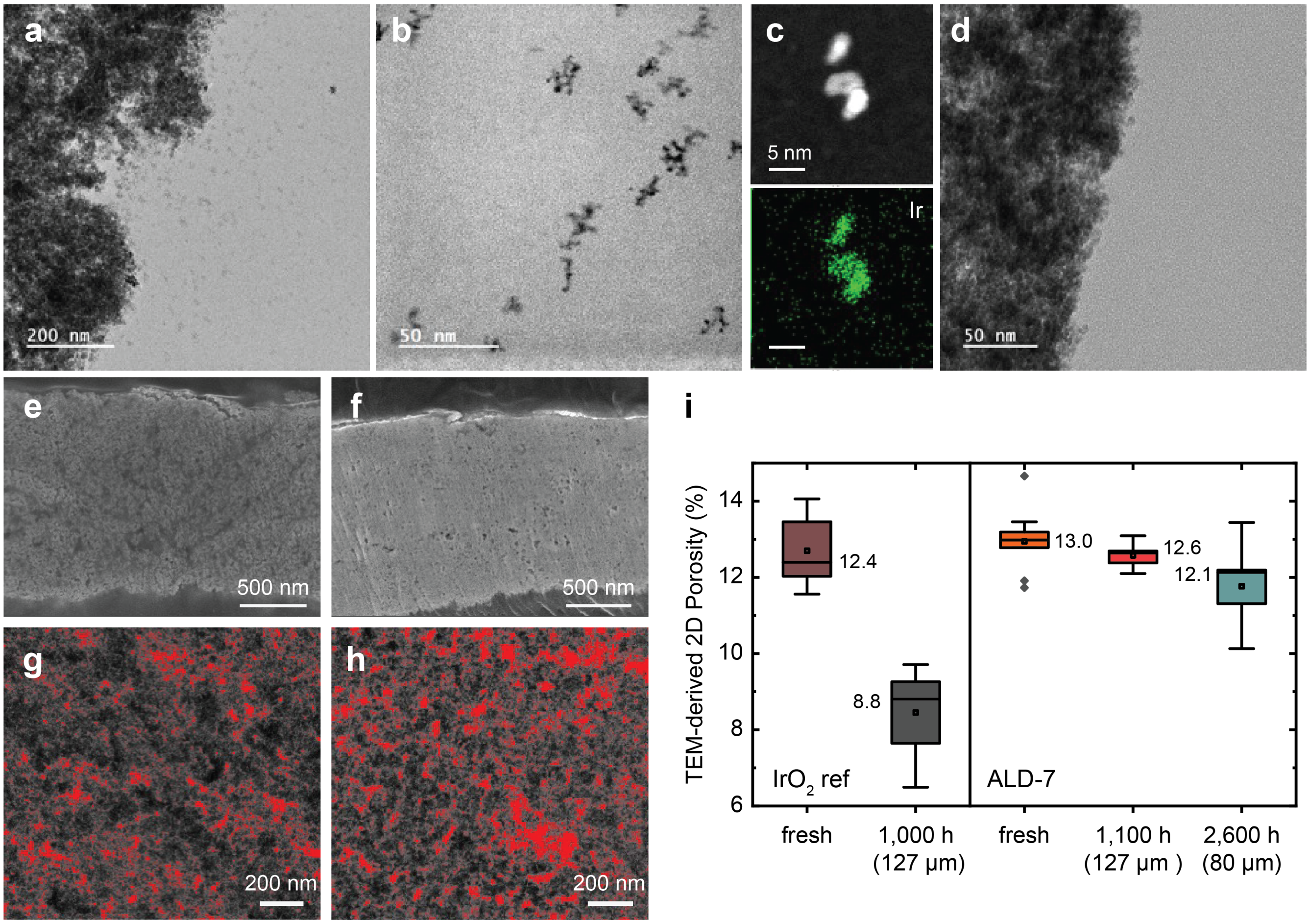

Fig. 3 | Post-mortem structural analysis of reference and TiO_2-coated IrO_2 anodes after long-term

PEMWE operation. Samples were assembled with a 127 µm membrane unless otherwise indicated. ... **i** Statistical quantification of catalyst-layer porosity (%) derived from TEM-based 2D digital image analysis for reference IrO_2 (127 µm membrane) and ALD-7 anodes (127 and 80 µm membranes) before and after operation, showing pronounced porosity loss for the reference anode and preservation of pore volume for the TiO_2-modified anode even after 2,600 h of operation with the 80 µm membrane.

The original Supplementary Fig. 18 has likewise been expanded into Supplementary Fig. 21 by adding panel *e*, which shows representative TEM images of the ALD-7 anode after 2,600 h of operation with the 80 µm membrane. The revised figure is shown below with changes in the caption underlined:

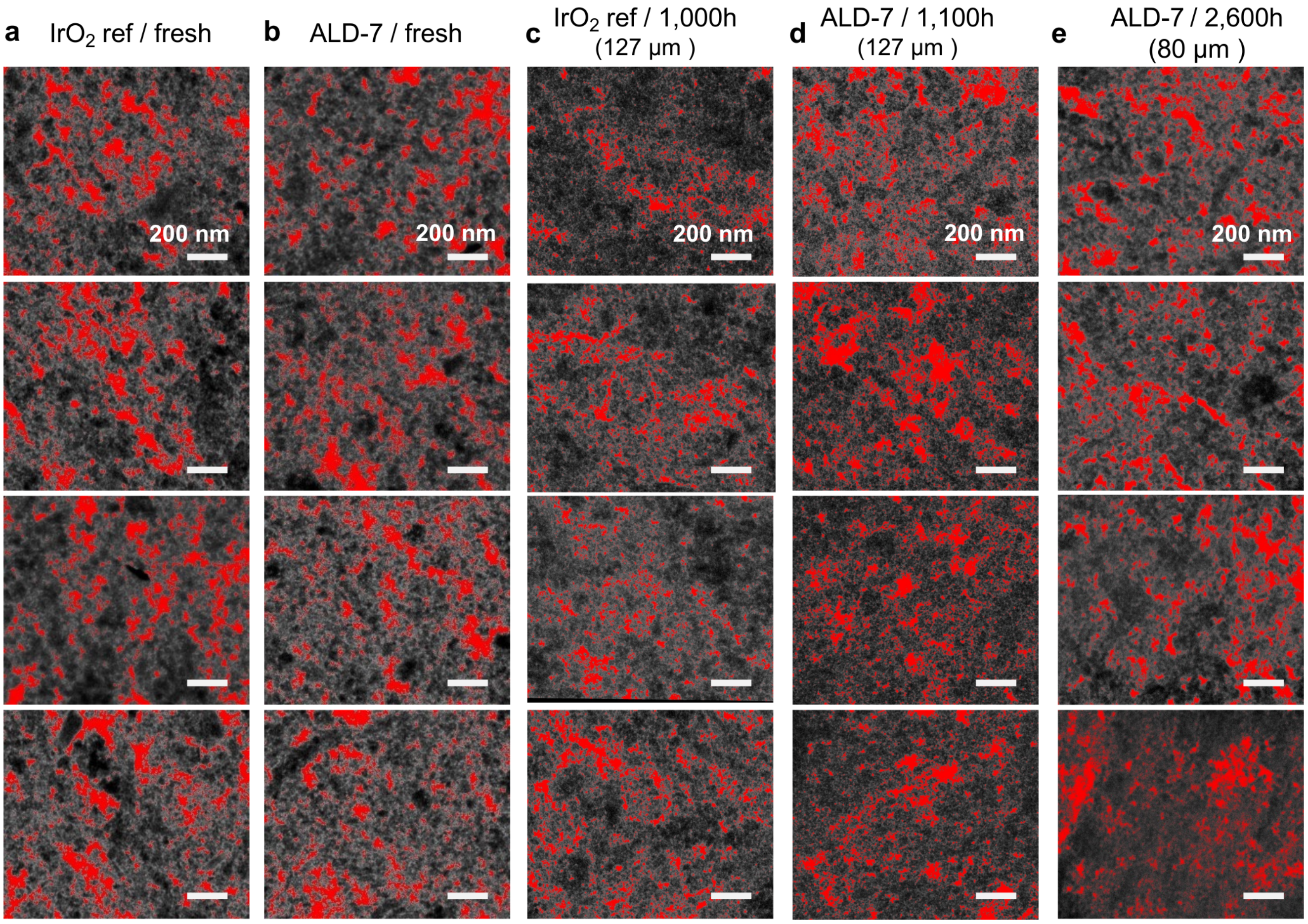

Supplementary Fig. 21 | Post-mortem cross-sectional TEM images used for quantitative porosity analysis. a, b, Representative cross-sectional TEM images of the fresh IrO_2 reference anode **(a)** and the ALD-7 anode **(b)**, prior to electrochemical testing. The red overlays indicate pore regions within the catalyst layers identified for quantitative porosity analysis. **c,d,** Representative cross-sectional TEM images acquired after operation at 3.0 A cm^{-2} for the IrO_2 reference anode (1,000 h) **(c)** and the ALD-7 anode (1,100 h) **(d)**, both assembled with a 127 µm membrane. The IrO_2 reference anode exhibits a pronounced reduction in pore area after 1,000 h of operation, whereas the ALD-7 anode largely preserves its pore area after 1,100 h. **e,** Representative cross-sectional TEM image of the ALD-7 anode after extended operation at 3.0 A cm^{-2} for 2,600 h with an 80 µm membrane, showing sustained preservation of the porous structure. Scale bars represent 200 nm for all panels.

The relevant text was also added to page 11 of the revised manuscript as follows with changes underlined:

“...Quantitative porosity analysis derived from TEM images also shows a decrease in porosity of the reference IrO_2 anode from 12.4% to 8.8% after operation with a 127 μm membrane, while the porosity of the ALD-7 anode changed marginally from 13.0% to 12.6% under the same conditions, and remained largely preserved at 12.1% even after extended operation for 2,600 h with an 80 μm membrane (Fig. 3i)...”

(2) ICP-MS analysis of the electrolyte: We additionally performed ICP-MS analysis of the anode water to quantify dissolved Ir species during PEMWE operation and to further support the long-term stability of the TiO_2-modified anode, as shown in Supplementary Fig. 15 below. The measured Ir concentrations for the reference IrO_2 anode increased from 0.04 ppb at the beginning of operation to 0.12 ppb after 24 h and 0.15 ppb after 138 h. In contrast, the ALD-7 anode exhibited substantially lower Ir concentrations of 0.02 ppb at 0 h, 0.04 ppb after 24 h, and 0.03 ppb after 138 h. These values remain close to the practical detection limit (approximately 0.1 ppb) of conventional ICP-MS measurements. These results indicate that the TiO_2-modified catalyst strongly suppresses Ir dissolution under PEMWE operating conditions.

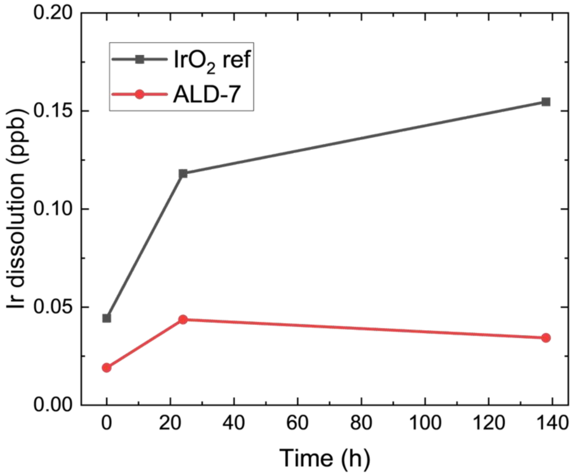

Supplementary Fig. 15 | Ir dissolution during PEMWE operation measured by ICP-MS. Ir concentrations detected in the anode outlet water for the IrO_2 reference and ALD-7 anodes as a function of operation time. The reference IrO_2 anode shows a progressive increase in dissolved Ir concentration during operation, whereas the ALD-7 anode maintains substantially lower Ir concentrations close to the practical detection limit of ICP-MS, indicating suppressed Ir dissolution.

The extremely low Ir concentrations observed for the ALD-7 anode are consistent with a recent report (*ACS Energy Lett.* **8**, 2682–2688 (2023)) showing that dissolved Ir species in PEMWE systems are often present only at sub-ppb to low-ppb levels because a significant fraction of dissolved Ir remains trapped within the membrane or redeposits at the cathode rather than exiting through the outlet water stream. Accordingly, the ICP-MS results, together with the absence of detectable Ir migration into the membrane region in cross-sectional TEM analysis (Fig. 3d), support the superior electrochemical stability of the TiO_2-modified anode. The relevant clarifications were added to pages 10–11 of the revised manuscript as follows, with changes underlined:

“...In contrast, the ALD-7 anode shows no detectable Ir migration after 1,100 h of operation (Fig. 3d). Inductively coupled plasma mass spectrometry (ICP-MS) analysis of the anode outlet water revealed substantially lower Ir dissolution for the ALD-7 anode than for the reference IrO_2 anode, with the dissolved Ir concentration remaining near the practical detection limit throughout operation (Supplemen-

tary Fig. 15). Independent ICP-OES analysis from three-electrode stability tests further confirms that the TiO_2 coating effectively suppressed Ir dissolution (Supplementary Note 4, Supplementary Figs. 16–19)...”

10. The manuscript shows that the 80 μm Nafion membrane performs better than the 127 μm membrane. Please explain why the 127 μm membrane was still selected for the performance evaluation of the 100 cm^2 MEA.

We thank the reviewer for this practical comment. We would like to clarify that the 100 cm^2 MEA shown in Fig. 6d and e (formerly presented as Supplementary Fig. 42 in the original SI) was in fact fabricated and tested using an 80 μm membrane, rather than a 127 μm membrane. The misunderstanding arose from an error in the original figure, where the membrane thickness was incorrectly labeled as 127 μm. This error has now been corrected in the revised manuscript, and the membrane thickness used in each case is explicitly specified. As correctly noted by the reviewer, thinner membranes are more representative of practical PEMWE operation due to their lower ohmic resistance, and the use of an 80 μm membrane in the large-area MEA further supports the industrial relevance of our results. We apologize for the confusion in the original SI.

In the course of revision, the original Supplementary Fig. 42 has been promoted to the main Fig. 6 alongside the roll-to-roll process panels. The corrected polarization curves (Fig. 6e) now display the proper “ALD-7 (80 μm) at 50 °C” annotation as shown below, with the additions underlined:

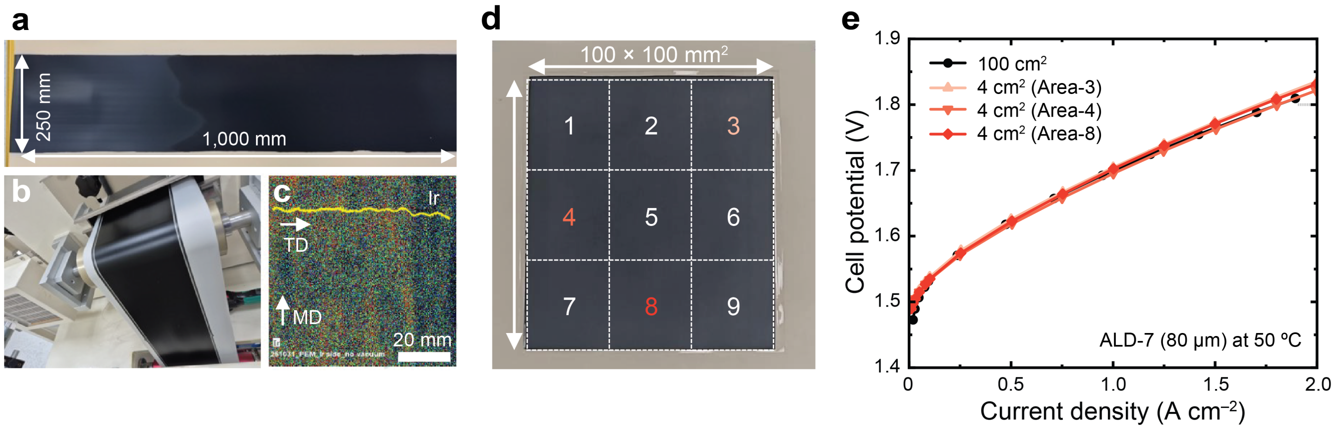

Fig. 6 | Meter-scale roll-to-roll manufacturing and large-area processability of TiO_2 coating-based PEMWE systems. a,b Photographs of the continuous roll-to-roll coating process validating the scalability of the TiO_2-coated catalyst architecture toward industrial manufacturing. **c** XRF spectral mapping of the Ir-Lα line across a 100 cm^2 MEA fabricated with ALD-7 by roll-to-roll processing. The map illustrates the spatial distribution of Ir loading along the MD and TD. **d** Photographic image of a 100 × 100 mm^2 (100 cm^2) MEA with the ALD-7 anode. The overlaid grid defines nine sub-regions (Areas 1–9) used for large-area spatial evaluation of catalyst loading and performance uniformity. **e** Polarization curves of PEMWE cells assembled with the full 100 cm^2 MEA and representative 4 cm^2 MEAs extracted from Areas 3, 4, and 8. The nearly identical electrochemical responses confirm robust large-area scalability of the TiO_2-ALD-based anode architecture.

We have also added clarifying texts at two locations in the revised manuscript as follows, with changes underlined:

Page 6–7 in the electrochemical results section:
“PEMWE cells were subsequently evaluated for long-term stability under galvanostatic operation at

3.0 A cm^{-2} that represents a stringent durability condition. When paired with a 127 μm membrane, a thickness commonly used in PEMWE research, the reference IrO_2 anode exhibits...”

“...When paired with an 80 μm membrane, representative of low-resistance industrial operation, the ALD-7 anode operates stably for 2,600 h, further underscoring the durability enhancement under harsh operating conditions.”

Page 17 in the roll-to-roll section:
“A continuous web with a width of 250 mm and a length of ∼5 m was successfully produced (Fig. 6a,b), and used to fabricate a 100 cm^2 MEA (100 mm × 100 mm) paired with an 80 μm membrane...”

Response to the Reviewer #3:

This study addresses durability/cost challenges of iridium-based anodes in high-current PEM water electrolyzers via a dual-interface strategy using TiO_2 ALD on IrO_2. It enables 2600 h stable operation at 3.0 $A\,cm^{-2}$ with near-zero voltage decay at 0.4 $mg\,cm^{-2}$ Ir loading—vastly outperforming bare IrO_2. Mechanistically, Ti−O−Ir coupling inhibits Ir dissolution; TiO_2 coating boosts ionomer interaction/mass transport. Roll-to-roll compatible, it cuts capital costs by 22% and projects \$3.2/kg H_2 at 1000 $MW\,yr^{-1}$. Critically, it offers a generalizable principle to extend electrolyzer lifetime and reduce noble-metal loading in electrochemical technology. Therefore, I would like to recommend the publication of this work, with the following revisions suggested.

We thank Reviewer #3 for the favorable assessment of our work and for recommending publication. We appreciate the recognition that the dual-interface strategy offers a generalizable design principle for extending electrolyzer lifetime and reducing noble-metal loading. The detailed suggestions regarding additional characterization, DFT model validation, and ionomer interaction analysis have been invaluable in refining our mechanistic arguments. We have addressed all comments with new experimental data, revised computational models, and expanded discussions as described below.

1. TEM-EDS only detects Ir deposited in the membrane, not dissolved Ir lost in the anode effluent. Moreover, Ir dissolution in a three-electrode system does not necessarily reflect that in a real membrane electrode assembly. Direct ICP analysis of the outlet water from PEMWE operation is needed to confirm that the TiO_2 coating truly suppresses total Ir dissolution rather than merely altering its transport and deposition behavior.

We thank the reviewer for this important comment. We have addressed this point in our response to *Point 9 from Reviewer #2* by performing additional ICP-MS analysis of the anode outlet water during PEMWE operation (Supplementary Fig. 15). The reference IrO_2 anode showed a progressive increase in dissolved Ir concentration over time, whereas the ALD-7 anode maintained Ir concentrations near the practical detection limit throughout operation. These results directly demonstrate that the TiO_2 coating suppresses outlet-detectable Ir dissolution from the catalyst layer, rather than merely altering its transport or deposition behavior. The corresponding manuscript revisions are shown in that response.

2. In the Ir 4f XPS fitting in Supplementary Fig. 19a, the spin–orbit splitting between the Ir $4f_{7/2}$ and $4f_{5/2}$ doublets is not held constant, violating a fundamental physical constraint. This inconsistency undermines the reliability of the derived Ir oxidation states and the conclusions drawn from the XPS analysis.

We thank the reviewer for this important comment and agree that, for physically meaningful peak deconvolution, the spin–orbit splitting between the Ir $4f_{7/2}$ and $4f_{5/2}$ components should be constrained to a constant value. We would like to clarify that Supplementary Fig. 19a was not intended to present a fitted XPS deconvolution. Instead, it was a simplified visualization of the apparent peak positions derived from the Ir 4f spectra shown in Fig. 4a, without applying constrained fitting procedures. We acknowledge that the presentation may have led to the impression that these peaks correspond to fitted doublets, which could cause confusion regarding the physical validity of the analysis. To avoid this ambiguity, we have removed Supplementary Fig. 19a in the revised SI. The remaining XPS panels are now consolidated as Supplementary Fig. 2 in the revised SI to align with the manuscript flow. Importantly, the conclusions regarding the electronic modulation of Ir are not based on this supplementary visualization but are supported by the systematic shift of the Ir 4f envelope in Fig. 4a, together with consistent trends observed in Ir L_3-edge XANES (Fig. 4b) and EXAFS (Fig. 4c), as well as DFT analysis (Fig. 4d–f).

3. The main text mentioned the Ti-induced electronic modulation suppresses Ir over-oxidation, however post characterization of the Ir oxidation state after long-term operation is missing.

We thank the reviewer for this important comment. This point has been addressed through the addition of post-operation Ir 4f XPS characterization of the ALD-7 anode after long-term operation (Supplementary Fig. 27), as discussed in our responses to *Points 2 to 4 from Reviewer #2*. The results show that the Ir oxidation state remains close to its initial state, supporting the suppression of over-oxidation during operation.

4. The authors attribute the initial voltage reduction of TiO_2 coated anodes primarily to improved mass transport, while noting that partial coverage of Ir active sites by TiO_2 would otherwise be detrimental. However, the manuscript does not provide electrochemically active surface area measurements or specific activity for the different catalysts.

We appreciate the reviewer for this important comment. This point has been addressed by incorporating RDE-based ECSA measurements and ECSA-normalized activity analysis (Supplementary Fig. 12, Supplementary Tables 4 and 5, and Supplementary Note 2) as detailed in our responses to *Point 1 from Reviewer #1* and *Point 5 from Reviewer #2*. These results show that while the ECSA decreases with increasing ALD cycles due to partial surface coverage, the intrinsic activity (j_{ECSA}) of ALD-3 to ALD-7 remains comparable to that of state-of-the-art Ir-based catalysts, confirming that the TiO_2 modification does not significantly compromise intrinsic catalytic activity.

5. Static contact angle measurements alone are insufficient to prove enhanced bubble detachment under operating conditions. Operando optical or high-speed imaging is needed to directly verify that the TiO_2 coating reduces bubble accumulation and thereby lowers mass transport overpotential at 3.0 A cm^{-2}.

We thank the reviewer for this comment. We agree that operando bubble visualization would provide the most direct evidence. However, direct optical imaging of the anode catalyst layer during PEMWE operation at 3.0 A cm^{-2} is not feasible in the present cell configuration, as the catalyst layer is confined between the proton-exchange membrane and the opaque Ti porous transport layer. Modified cell designs that enable optical access would substantially alter the flow-field geometry, compression, and thermal conditions, making direct comparison with the performance data presented in this work difficult.

Nevertheless, the static contact angle and aerophobic angle measurement (Supplementary Fig. 11) provide ex situ evidence that the TiO_2-modified surface exhibits modestly higher hydrophilicity and greater aerophobicity, properties that are thermodynamically favorable for bubble detachment. More importantly, several quantitative, operationally derived metrics captured during the actual 3.0 A cm^{-2} tests provide functional evidence consistent with reduced bubble accumulation:

(1) Mass-transport overpotential ($\Delta\eta_{mt}$) evolution: The overpotential decomposition (Fig. 2e,f) shows that $\Delta\eta_{mt}$ increases by +8.8 mV for the reference IrO_2 anode with the 127 μm membrane after 1,000 h, whereas $\Delta\eta_{mt}$ decreases for the ALD-7 anodes by –14.6 mV with the 127 μm membrane and –3.1 mV with the 80 μm membrane. This negative $\Delta\eta_{mt}$ is a direct, quantitative indicator of improved mass transport during operation, not an ex situ inference.

(2) Suppressed voltage oscillation amplitude: During the galvanostatic operation at 3.0 A cm^{-2}, the reference IrO_2 anode exhibits reversible voltage fluctuations of ~100 mV, whereas the ALD-7 anode shows substantially suppressed oscillations of ~30 mV (Fig. 2b). These fluctuations are widely attributed to

periodic oxygen bubble accumulation and detachment within the catalyst layer and porous transport layer (*Fuel* **386,** 134344 (2025)). The threefold reduction in oscillation amplitude during actual high-current operation constitutes in situ functional evidence of mitigated bubble-induced transport limitations.

(3) Preserved pore architecture: Post-mortem TEM porosity analysis (Fig. 3i) confirms that the ALD-7 anode retains its pore network (13.0% → 12.6%) while the reference suffers substantial pore collapse (12.4% → 8.8%), consistent with maintained pathways for gas evacuation.

While operando imaging remains a valuable direction for future work, the convergence of ex situ wettability measurements, in situ voltage oscillation behavior, quantitative overpotential decomposition, and post-mortem structural analysis collectively provides strong evidence that the TiO_2 coating mitigates bubble-induced transport limitations under operating conditions. To emphasize these operationally derived metrics alongside the ex situ contact angle data, we have added the relevant text to page 7 of the revised manuscript as follows:

"...The threefold reduction in voltage oscillation amplitude provides in situ functional evidence of suppressed bubble-induced transport limitations during 3.0 A cm^{-2} operation, complementing the enhanced aerophobicity observed in ex situ wettability measurements (Supplementary Fig. 11)..."

6. The pore size distributions derived from BET shows a dV_p/dd_p at ∼12 nm, while the pore size distributions derived from mercury intrusion porosimetry reveal that ALD-7 have an obvious higher pore area. Why the pore size distributions from these two methods show different results?

We thank the reviewer for this observation. The apparent discrepancy arises because the two methods probe different pore size regimes.

BJH analysis from N_2 physisorption (Supplementary Fig. 3b) reflects mesopores of ∼2–50 nm that are intrinsic to the catalyst powder in the absence of an ionomer. The peak at ∼12 nm is characteristic of IrO_2 nanoparticle morphology, and because TiO_2 ALD at 7 cycles deposits a sub-nanometer-scale surface modification, these intrinsic mesopores remain largely unchanged.

Mercury intrusion porosimetry (MIP, Fig. 1f) probes larger inter-aggregate pores of approximately 10 nm to 1 μm within the fabricated electrode, which are governed by particle packing and ionomer distribution. In this context, the aggregate size and colloidal behavior of the catalyst ink play a critical role in determining the resulting electrode microstructure, as they directly influence particle packing and pore formation during electrode fabrication. TiO_2 modification reduces aggregate size both in the absence of ionomer (from 1,697 to 908 nm) and in the presence of ionomer (from 1,014 to 270 nm) as shown in Fig. 5a. The size reduction without ionomer originates from TiO_2-induced surface hydrophilicity that improves dispersion, while the further reduction with ionomer reflects enhanced ionomer adsorption onto the TiO_2-modified surface, as confirmed by ITC (Fig. 5c), ^{19}F NMR (Supplementary Fig. 30), and DFT binding energy calculations (Fig. 5f). Improved colloidal dispersion and stronger ionomer–catalyst interactions together produce the more uniform and open electrode-level pore architecture observed by MIP.

The two techniques are therefore complementary. BJH confirms that intrinsic catalyst mesoporosity is preserved, while MIP reveals that electrode-level macropore structure is improved through enhanced colloidal stability and ionomer–catalyst interactions.

7. Page 5, Line 105, "the TiO_2 coating modulates catalyst surface characteristics and thereby establishes a uniform pore architecture and favorable wettability across the electrodes." However, it's hard to say that there is a big difference between the contact angle of 119.7° and 112.8°, which may have minor influence on water spreading and gas bubble detachment.

This point was addressed in our response to *Point 1 from Reviewer #2*. We agree that the electrode-level contact angle difference (∼7º) is modest, and the revised manuscript now frames the catalyst surface modification as exerting its primary influence by governing adsorption and organization of ionomer sulfonate groups at the catalyst–ionomer interface, rather than through macroscopic wettability alone. The revised text is reproduced in that response.

8. The authors claimed that the wettability contrast facilitates water spreading and gas bubble detachment. However, there seems to be no experiment evidence to support this viewpoint. More experiments like bubble visualization of catalytic layer are suggested to be performed to support this viewpoint.

We thank the reviewer for this valuable suggestion. As discussed in our response to *Point 5 from Reviewer #3*, direct operando bubble visualization within the catalyst layer is not feasible in the present PEMWE cell configuration. Instead, we provided multiple complementary pieces of indirect but operationally relevant evidence supporting mitigated bubble-induced transport limitations in the TiO_2-modified anodes, including: (i) decreased mass-transport overpotential, (ii) suppressed voltage oscillation amplitudes, (iii) preserved pore architecture. Taken together, these results collectively support the interpretation that the TiO_2 modification facilitates improved gas evacuation and mitigates bubble-induced transport limitations under practical operating conditions.

9. ALD-7 shows exhibits a uniform Ti distribution at IrO_2 surface, but Fig. 2a shows the catalyst with best activity is ALD-3. Why chooses ALD-7 as the target sample? And as the cycle numbers changes, what dominates the activity change?

We thank the reviewer for this insightful comment. As clarified in our response to *Point 5 from Reviewer #2*, the activity trend with ALD cycle number can be understood by considering both ECSA-normalized intrinsic activity and mass-transport effects. RDE measurements show that the intrinsic activity (j_{ECSA}) of ALD-3, ALD-5, and ALD-7 remains comparable, indicating that TiO_2 coating does not significantly alter the intrinsic catalytic activity in this regime (Supplementary Fig. 12, Supplementary Tables 4 and 5, and Supplementary Note 2). In contrast, ALD-10 exhibits a clear decrease in j_{ECSA} due to excessive surface coverage. Despite the similar intrinsic activity, the iR-free cell voltage at 3.0 $A\,cm^{-2}$ is higher for ALD-3 than for ALD-5 and ALD-7. This difference arises from insufficient mitigation of mass-transport limitations at low Ti coverage, where improvements in wettability and pore structure are not fully developed. Therefore, ALD-7 was selected as the representative sample because it provides an optimal balance: it maintains intrinsic activity comparable to lower cycle numbers while more effectively alleviating mass-transport limitations, resulting in superior full-cell performance under high current density.

10. Standard tests with RDE should be performed to fully evaluate the performance and mechanism of the catalysts. After ALD cycles, the ECSA may decrease and the inherent activity may change. These elements should be considered.

We thank the reviewer for this valuable suggestion. This point has been addressed through the inclusion of RDE measurements, as detailed in our responses to *Point 1 from Reviewer #1, Point 5 from Reviewer #2*, and *Point 9 from Reviewer #3* (Supplementary Fig. 12, Supplementary Tables 4 and 5, and Supplementary Note 2). Specifically, we evaluated the ECSA and ECSA-normalized activity (j_{ECSA}) for catalysts with different ALD cycles. The results show that while the ECSA decreases with increasing ALD cycles, the intrinsic activity remains comparable for ALD-3 to ALD-7 and decreases only at the highest cycle number (ALD-10). These findings have been incorporated into the revised manuscript.

11. From TEM images, Ti atoms are located onto IrO_2 surface and form heterogeneous interfaces. However, the DFT-calculated atomic model uses a Ti doped IrO_2 model, which may be not match with reality.

We sincerely thank the reviewer for this insightful comment regarding the apparent discrepancy between the TEM observations and the DFT models. We agree that the TEM images indicate that Ti species are primarily located on the IrO_2 surface and form heterogeneous Ti−O−Ir interfaces rather than a bulk-doped structure. We would like to clarify that our DFT models are not intended to reproduce the exact atomic structure observed experimentally, but rather to capture the local electronic interactions between Ti species and neighboring Ir sites. To this end, we considered two representative configurations: (i) a Ti-attached model, where Ti is bonded to the IrO_2 surface, and (ii) a Ti-doped surface model, where Ti is incorporated into the topmost IrO_2 lattice (Supplementary Fig. 25). These configurations serve as limiting cases that bracket the possible local coordination environments at the Ti−O−Ir interface.

In particular, the Ti-substituted IrO_2 model was employed in the analysis of the Pourbaix diagram and oxygen vacancy formation energy (Supplementary Fig. 26), as a simplified framework to systematically evaluate how Ti incorporation modulates the electronic structure of adjacent Ir atoms through Ti−O−Ir linkages. The inclusion of the Ti-doped model is further supported by several considerations. The ALD process followed by annealing at 400 °C may induce partial interfacial mixing within the near-surface region, which is not directly resolved by top-view TEM (Fig. 1b–d). In addition, TiO_2 and IrO_2 share the same rutile structure with similar lattice parameters, indicating high structural compatibility. Consistently, our DFT calculations show that Ti incorporation into the IrO_2 surface is thermodynamically favorable, in agreement with recent reports (e.g., *Small Struct.* **2024**, 5, 2300276). The consistent trends obtained from both Ti-doped and Ti-attached models support our key conclusion that Ti−O−Ir interfacial interactions reduce the effective oxidation state of Ir and contribute to catalyst stabilization.

We have revised the manuscript to avoid potential confusion and to explicitly clarify the role and limitations of the DFT models, which represent simplified local motifs rather than exact structural replicas of the experimental system. We have clarified this point on page 12 of the revised manuscript as follows:

"To model the Ti−O−Ir interfacial interaction, we employed both Ti-attached and Ti-substituted IrO_2(110) surface models in density functional theory (DFT) calculations. These configurations represent limiting cases of local coordination environments at the interface, enabling systematic evaluation of the electronic modulation of Ir sites induced by Ti species. While the "Ti-attached" model reflects surface-bound Ti species observed by TEM, the Ti-substituted model ("Ti-doped") represents a more strongly coupled configuration that may arise from interfacial mixing during post-annealing. Accordingly, these models are intended to capture local electronic interactions rather than to reproduce the exact atomic structure of the

catalyst (Supplementary Fig. 25). Based on these models, we first examined the electronic perturbation induced by Ti at the IrO_2 surface..."

12. The authors claims that Ti induced electronic stabilization of Ir mitigates over-oxidation-driven dissolution. However, there seems to be no evidence that IrO_2 dissolve because over-oxidation and Ti doping suppress this process. XPS or XAFS after OER should be provided and analyzed.

We thank the reviewer for this comment. This point was addressed in our response to *Point 2 from Reviewer #2*. XPS, XANES, and DFT Bader charge analyses (Fig. 4a-f) consistently show that interfacial Ti−O−Ir coupling lowers the Ir valence state through electronic modulation of neighboring Ir sites. Under oxidative OER conditions, this Ti-induced electronic downshift is expected to suppress the formation of unstable over-oxidized Ir species (Ir^{5+}/Ir^{6+}), thereby stabilizing the oxide framework. Consistently, DFT calculations show that Ti incorporation widens the thermodynamically stable potential–pH window and increases the oxygen-vacancy formation energy (Supplementary Fig. 26), indicating reduced susceptibility to lattice-oxygen-mediated degradation.

We have performed post-operation XPS on the ALD-7 anode after long-term testing at 3.0 $A\,cm^{-2}$ (Supplementary Fig. 27). The Ir 4f binding energy remains close to the pre-operation state, suggesting that the Ir oxidation state does not evolve toward over-oxidized states during operation. Combined with the suppressed Ir dissolution confirmed by post-mortem TEM (Fig. 3a–d), ICP-MS, and ICP-OES (Supplementary Figs. 15–19), these results consistently support the interpretation that Ti-induced electronic stabilization mitigates over-oxidation-driven dissolution.

13. TiO_2 coating enhances ionomer adsorption capacity and binding strength, therefore stabilizing the catalyst surface and catalyst-layer architecture. However, several literatures have pointed out that strong adsorption of anionic sulfonate groups can lead to the sedimentation of catalysts in catalyst inks, and block active sites (*Nat. Catal.* **2023**, *6* (5), 392–401.). Why in this work, the binding of catalysts and anionic sulfonate groups have the opposite effect?

We thank the reviewer for this important comment and for highlighting the relevant literature on ionomer–catalyst interactions in fuel-cell systems. We agree that strong adsorption of sulfonate groups can be detrimental in certain contexts, particularly for metallic Pt catalysts used in the oxygen reduction reaction (ORR), where direct adsorption of anionic species on Pt surfaces can block active sites and reduce intrinsic activity, as demonstrated in *Nat. Catal.* **2023**, *6* (5), 392–401. However, the present system differs fundamentally in both catalyst chemistry and reaction environment.

First, the catalyst in this work is an oxide (IrO_2/TiO_2) rather than a metallic Pt surface. In Pt-based systems, sulfonate adsorption occurs directly on metallic active sites, leading to site blocking. In contrast, in the present system, ionomer adsorption predominantly occurs on TiO_2-modified oxide surfaces, which are not equivalent to discrete metallic active sites. As a result, the direct poisoning effect is significantly mitigated, and additional blocking of catalytically relevant Ir sites is expected to be limited.

Second, the functional role of ionomer–catalyst interaction differs between PEM fuel cells and PEM water electrolysis. In ORR systems, maximizing access to Pt active sites is critical, and strong ionomer adsorption is therefore detrimental. In contrast, for the oxygen evolution reaction (OER) under high-current-density PEMWE conditions, efficient water supply and rapid removal of evolved O_2 are key performance-limiting factors. In this context, the ionomer plays an essential role in maintaining proton conduction pathways and stabilizing the catalyst-layer architecture.

In the present work, the enhanced ionomer interaction induced by TiO_2 modification contributes primarily to improved catalyst-layer integrity and transport properties, rather than to increased site blocking. Specifically, stronger ionomer–catalyst interaction helps maintain a uniform pore structure, prevents structural collapse during prolonged operation, and facilitates more stable water/gas distribution, thereby mitigating mass-transport limitations. These effects are directly reflected in the reduced mass transport overpotential ($\Delta\eta_{mt}$) and suppressed reversible voltage increase observed for the TiO_2-modified anodes (Fig. 2b,e). Taken together, while strong ionomer adsorption can be detrimental in Pt-based ORR systems, in the present oxide-based OER system, controlled enhancement of ionomer–catalyst interaction plays a structurally beneficial role, improving catalyst-layer stability and mass transport under practical high-current operation. In the revised manuscript, we have incorporated a brief discussion of the relevant literature (*Nat. Catal.* **6,** 392-401 (2023)) on pages 16-17 as follows, with changes underlined:

"Collectively, the Ti-induced electronic modulation via interfacial Ti−O−Ir coupling not only suppresses Ir over-oxidation but also strengthens the electrostatic interaction with the ionomer, thereby stabilizing both the catalyst surface and catalyst-layer architecture and improving PEMWE durability. Previous studies have shown that strong sulfonate adsorption can block active sites on metallic Pt catalysts.[48] In contrast, in the present oxide-based system, ionomer interaction occurs primarily at the TiO_2-modified interface and contributes to catalyst-layer stabilization, rather than direct active-site blocking..."

48. Chen, F. *et al.* Blocking the sulfonate group in Nafion to unlock platinum's activity in membrane electrode assemblies. *Nat. Catal.* **6,** 392-401 (2023)."

14. Supplementary Fig. 19a, the fitting parameters should be provided. The X-axis of XPS results should be uniform.

We thank the reviewer for this comment. This point has been addressed in our response to *Point 2 from Reviewer #3*. As clarified there, Supplementary Fig. 19a did not represent a physically constrained XPS fitting, which may have led to confusion regarding fitting parameters and peak alignment. We have removed Supplementary Fig. 19a in the revised SI to avoid this ambiguity. In the course of the revision, the remaining XPS panels of the original Supplementary Fig. 19 have been relocated to Supplementary Fig. 2 to align with the manuscript flow.

Response to the Reviewer #1:

I have reviewed the authors' revised manuscript and their detailed point-by-point response to my previous comments. I am pleased to conclude that all of my concerns have been addressed.

Recommendation: Accept

We sincerely thank Reviewer #1 for the careful evaluation of our manuscript and for the positive recommendation. We greatly appreciate the constructive comments provided throughout the review process which have substantially strengthened the manuscript.

Response to the Reviewer #2:

The authors have made substantial revisions and provided additional experimental results and analyses that significantly improve the overall quality and comprehensiveness of the manuscript. The work is now suitable for publication in its current form. Nevertheless, a few issues remain that should be further clarified or improved:

We sincerely thank Reviewer #2 for the positive assessment of our revised manuscript and for recognizing the improvements made during the revision. We appreciate the additional comments, which we have carefully addressed point by point below.

1. The XPS results and the increased oxygen-vacancy formation energy primarily indicate enhanced lattice stability. However, they do not directly demonstrate the suppression of lattice oxygen participation during the OER process. The authors are encouraged to provide more direct evidence, such as isotope-labeling experiments, to substantiate this claim, or alternatively, to moderate the corresponding conclusions in the manuscript.

We thank the reviewer for this constructive comment. We agree that the XPS results and the increased oxygen-vacancy formation energy primarily demonstrate enhanced lattice stability and do not directly confirm the suppression of lattice oxygen participation during the OER. Direct verification of lattice oxygen participation would require isotope-labeling experiments, such as operando differential electrochemical mass spectrometry (DEMS) with ^{18}O-labeled catalysts. Such measurements demand a specialized operando configuration that is not compatible with our membrane electrode assembly setup, and they fall outside the scope of the present study. In accordance with the reviewer's suggestion, we have moderated the corresponding conclusion in the "Electronic modulation of IrO_2 by TiO_2 coatings" section. The revised text now frames the evidence as supporting enhanced oxygen-sublattice stability rather than as a direct demonstration of suppressed lattice oxygen participation. The revised text on pages 12–13 is as follows, with changes underlined:

"...These findings indicate that interfacial Ti−O−Ir coupling stabilizes the oxygen sublattice<u>, consistent with a reduced tendency toward</u> lattice-oxygen participation during OER.[26,28,29,44] <u>We note that the present XPS and oxygen-vacancy formation energy analyses primarily reflect enhanced lattice stability, and that a direct confirmation of suppressed lattice-oxygen participation would require isotope-labeling studies. Beyond this lattice stabilization,</u> the lower baseline Ir valence state increases the potential required to access highly oxidized Ir species (Ir^{5+}/Ir^{6+}) which are associated with transient dissolution under strongly anodic conditions.[13,14,22]..."

2. Supplementary Table 5 should be expanded to include more representative studies on Ir-based catalysts. In addition, important benchmarking parameters, such as Ir loading and overpotential, should be incorporated to enable a more comprehensive comparison.

We thank the reviewer for this helpful suggestion. In response, we substantially expanded Supplementary Table 5 by incorporating four additional representative studies on Ir-based OER catalysts for which ECSA-normalized activity was reported or could be extracted from the literature. We also added additional benchmarking parameters, including the RDE catalyst loading, overpotential at 10 mA cm^{-2}, PEMWE Ir loading, and PEMWE cell potential at the reported operating current density, to provide a more comprehensive comparison.

The revised comparison shows that the RDE catalyst loading used in this work is comparable to those reported in representative literature studies. Although the geometric overpotential at 10 mA cm^{-2} is somewhat higher than several benchmark catalysts, the ECSA-normalized activity remains within a similar range, indicating that the intrinsic surface activity is not substantially compromised by the TiO_2-ALD surface modification. In addition, the PEMWE cell potential of this work remains comparable to representative literature reports under practical operating conditions, while the degradation rate and Ir-mass-normalized charge density demonstrate substantially improved durability under high-current-density PEMWE operation.

We would also like to clarify that the primary purpose of Supplementary Table 5 is to demonstrate that the TiO_2-modified catalyst improves durability without significantly sacrificing the intrinsic catalytic activity of Ir-based catalysts. Accordingly, the central claim of this work is not the enhancement of intrinsic OER activity itself, but rather the simultaneous realization of comparable intrinsic activity and substantially improved PEMWE durability at low Ir loading and high current density. The revised Supplementary Table 5 is shown below, with changes highlighted in blue:

Supplementary Table 5 | Benchmarking ECSA-normalized activity of Ir-based catalysts.

Catalyst	RDE measurements			PEMWE measurements				Reference
	**Loading** mg cm^{-2}	**Overpotential** mV at 10 mA cm^{-2}	j_{ECSA} mA cm^{-2}	**Ir loading** mg cm^{-2}	**Cell voltage** V	**Deg. Rate** mV kh^{-1}	**Mass-norm. charge,** kAh mg^{-1}	
**TiO_2-coated IrO_2**	0.20	378	0.022 (1.55 V)	0.40	1.94 (3 A cm^{-2}, 80 °C)	0	19.5	This work
**Ir/Nb_2O_{5-x}**	1.6	218	0.0275 (1.55 V)	3.0	1.84 (3 A cm^{-2}, 80 °C)	0	1.33	*Angew. Chem. Int. Ed.* **61**, e202212341 (2022).
**Ru_6IrO_x**	0.40	240	0.021 (1.55 V)	0.38	1.94 (2 A cm^{-2}, 60 °C)	2.3	7.89	*Nat. Nanotechnol.* **20**, 1787–1795 (2025).
**IrO_x/t-ZrO_2**	1.0	288	0.0325 (1.55 V)	0.10	1.89 (3 A cm^{-2}, 80 °C)	6.25	16.0	*Energy Environ. Sci.* **18**, 5470–5481 (2025).
**IrO_x**	0.28	263	0.035 (1.54 V)	0.28	1.90 (3 A cm^{-2}, 80 °C)	7.0	12.9	*Nat. Commun.* **16**, 7608 (2025).
**Ti@Ir**	0.35	272	0.023 (1.53 V)	0.50	1.86 (2 A cm^{-2}, 80 °C)	1.25	7.20	*Chem. Eng. J.* **506**, 160118 (2025).
**$Sr_{0.9}Co_{0.9}Ir_{0.1}O_{3-\delta}$**	0.51	240	0.029 (1.50 V)	-	-	-	-	*ACS Catal.* **13**, 5007 (2023).
**$Ir_{0.063}Co_{2.937}O_4$**	0.24	266	0.099 (1.53 V)	-	-	-	-	*ACS Appl. Nano Mater.* **7**, 487–497 (2024).

3. The white labels in Fig. 6b and 6c are difficult to distinguish from the background and can be easily overlooked. The authors are advised to replace them with black or other higher-contrast colors to improve readability.

We thank the reviewer for this helpful suggestion. We have improved the contrast of the labels in both panels. In Fig. 6b, the label has been changed to black for clear contrast against the background. In Fig. 6c, the multicolored background makes black labels difficult to read across all regions, so we have retained white labels and added a dark gray shadow to enhance their visibility and legibility throughout the panel. The revised figure is shown below:

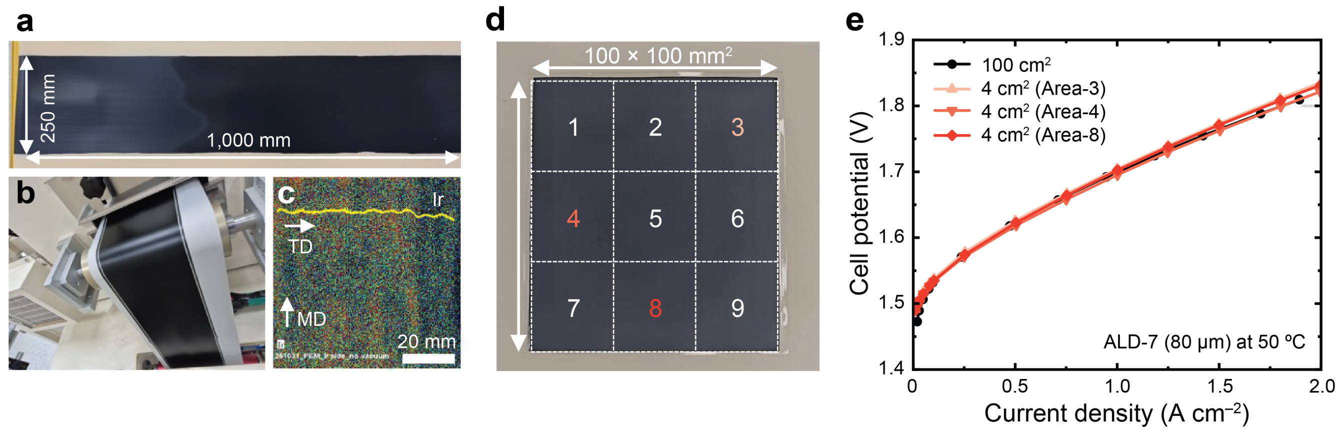

Fig. 6 | Meter-scale roll-to-roll manufacturing and large-area processability of TiO_2 coating-based PEMWE systems. a,b Photographs of the continuous roll-to-roll coating process validating the scalability of the TiO_2-coated catalyst architecture toward industrial manufacturing. **c** XRF spectral mapping of the Ir-Lα line across a 100 cm^2 MEA fabricated with ALD-7 by roll-to-roll processing. The map illustrates the spatial distribution of Ir loading along the MD and TD. **d** Photographic image of a 100 $\times$ 100 mm^2 (100 cm^2) MEA with the ALD-7 anode. The overlaid grid defines nine sub-regions (Areas 1–9) used for large-area spatial evaluation of catalyst loading and performance uniformity. **e** Polarization curves of PEMWE cells assembled with the full 100 cm^2 MEA and representative 4 cm^2 MEAs extracted from Areas 3, 4, and 8. The nearly identical electrochemical responses confirm robust large-area scalability of the TiO_2-ALD-based anode architecture.

Response to the Reviewer #3:

The authors have addressed my questions.

We sincerely thank Reviewer #3 for the careful evaluation of our manuscript and for the constructive comments throughout the review process, which have substantially improved the quality and clarity of our work.
